# Novel Multiple Time-grid Continuous-time Mathematical Formulation for Short-term Scheduling of Multipurpose Batch Plants

**DOI:** 10.1021/acs.iecr.2c01363

**Published:** 2022-10-19

**Authors:** Dan Li, Nikolaos Rakovitis, Taicheng Zheng, Yueting Pan, Jie Li, Giorgos Kopanos

**Affiliations:** † Centre for Process Integration, Department of Chemical Engineering, The University of Manchester, Manchester M13 9PL, U.K.; ‡ Department of Chemical Engineering, The University of Manchester, Manchester M13 9PL, U.K.; § Flexciton Limited, 145 City Rd, Hoxton, London EC1V 1AZ, U.K.

## Abstract

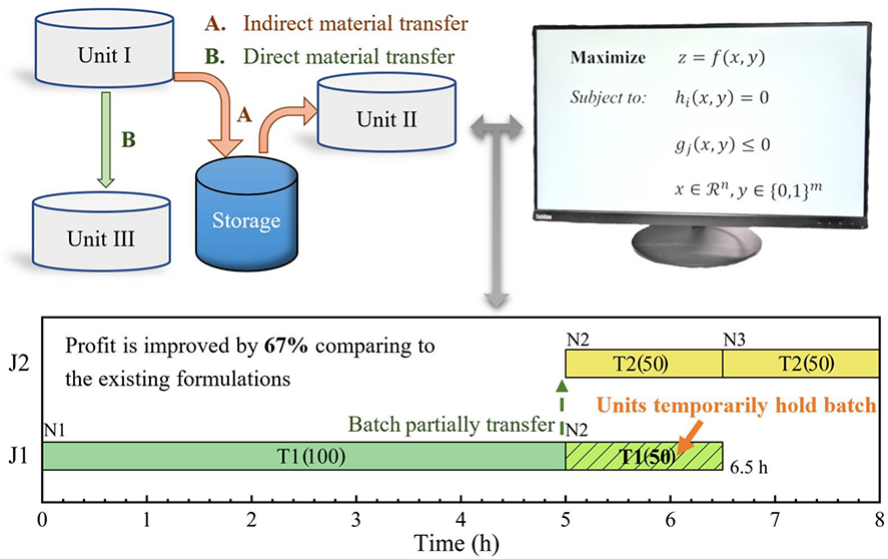

In this work, we have developed two novel unit-specific event-based mixed-integer linear programing models for scheduling multipurpose batch plants. The concept of indirect and direct material transfer is introduced to rigorously sequence and align tasks in different units. A batch after production is allowed to be partially transferred to storage and downstream processing units or held in processing units over multiple event points. The computational results demonstrate that the proposed models require a smaller number of event points in many cases to achieve optimality than existing unit-specific event-based models. It is interesting to find that no task is required to span over multiple event points to reach optimality for all addressed examples. The best variant developed is superior to existing unit-specific event-based models with the same or better objective values by a maximum improvement of 67%. The computational effort is significantly reduced by at least 1 order of magnitude in some cases.

## 1 Introduction

Multipurpose batch plants widely exist in the chemical industry for production of a large number of low-volume, high-value products. To achieve higher utilization of resources, lower inventory costs, and better responsiveness to a fluctuating manufacturing environment, optimal scheduling of multipurpose facilities is desirable and has attracted much interest of both academia and industry in the past decades. Many mathematical models have been developed based on either a state task network (STN)^1^ or a resource task network (RTN).^2^ These models can be classified into discrete- and continuous-time models.^3,4^ The continuous-time models can be further classified into global event-based,^5−7^ slot-based including process-slot based^8,9^ and unit-slot based,^10,11^ unit-specific event-based,^12−19^ and sequence-based^20−22^ models. These models can also be classified into single- and multiple-time grid models.^23^ For more details about these models, the readers are referred to the excellent review papers.^3,23,24^

All existing time-grid mathematical models divide the scheduling horizon using time points/slots/event points on which a task, operation, or activity can start, finish, or both. Therefore, the number of time points/slots/event points directly affects the efficiency of these mathematical models. More specifically, an increment in the number of time points/slots/event points can lead to an exponential increase in the model size (i.e., the number of binary variables, continuous variables, and constraints), which can potentially increase the computational time by at least 1 order of magnitude to generate the optimal solution. To reduce the required number of time points/slots/event points, Seid and Majozi^16^ observed that if there are enough materials in their dedicated storage for consumption, some sequence constraints related to different tasks in different units could be relaxed. They also implied that if there is sufficient storage space to hold materials, all related production and consumption tasks are not necessary to be rigorously aligned. Although their formulation did require a smaller number of event points in some cases, it could generate a schedule with real-time violation.^17^ Vooradi and Shaik^17^ noticed that if a consumption task consumes a state from a specifically related production task, this consumption task should start only after the specifically related production task instead of all related production tasks like Seid and Majozi.^16^ They also required related production and consumption tasks not to be rigorously aligned for sufficient storage space. They improved their previous formulation of Shaik and Floudas,^13^ which indeed further reduced the number of event points required without real-time violation in comparison to the model of Seid and Majozi.^16^ However, they introduced an increased number of binary variables, leading to computational inefficiency. In addition, their model can lead to a greater number of event points in many cases.

Besides the abovementioned two attempts, Vooradi and Shaik^25^ and Mostafaei and Harjunkoski^26^ attempted to eliminate some big-M constraints in their developed models, which could lead to much more slots/event points required and sub-optimality. More importantly, the multi-grid model of Mostafaei and Harjunkoski^26^ is applicable only when the storage capacity is unlimited. With limited or finite storage capacities, the schedules obtained from their models are often infeasible due to violation of storage capacities. Shaik and Vooradi^27^ and Rakovitis et al.^19^ imposed related production and consumption tasks to start at the same events. To improve the schedule accuracy for varying processing times from the discrete-time models, the combination of discrete- and continuous-time formulations was also investigated.^28,29^ In addition, machine learning techniques were also applied for online production scheduling.^30,31^

In this work, we mainly focus on the existing multiple time-grid continuous-time models, especially the unit-specific event-based models for short-term scheduling as their capabilities are well established in the literature.^13,14,19^ They are also the basis for the development of decomposition algorithms to solve industrial-scale scheduling problems. It is found that almost all existing unit-specific event-based models^16,17^ for scheduling of multipurpose batch plants still require a great number of event points in many cases, leading to computational inefficiency. More importantly, almost all existing models did not allow a batch of materials to be temporarily stored in processing units or be partially transferred to downstream units after production, leading to sub-optimality and inefficient utilization of processing units. Therefore, in this work, we have developed two novel multiple time-grid continuous-time formulations using the unit-specific event-based modeling approach to address the abovementioned limitations. The concept of indirect and direct material transfer is explicitly introduced to sequence different tasks in different units, which allow material transfer to be monitored between units rather than specific tasks to reduce the number of discrete decision variables and improve the computational efficiency. A batch of materials after production is allowed to be partially transferred to storage and downstream processing units or temporarily held in suitable processing units over multiple event points. A new continuous variable denoting the time when a state produced in a processing unit is available at an event point is introduced to assist the sequencing of different tasks related to the same states in different units, which overcomes the limitations in the existing unit-specific event-based models.^17^ To further reduce the computational expense, some new tightening constraints are proposed. A number of benchmark examples are solved to evaluate the performance of the proposed formulations. The computational results demonstrate that the best variant developed is superior to the existing unit-specific event-based models.

## 2 Problem Statement

**Figure 1:**
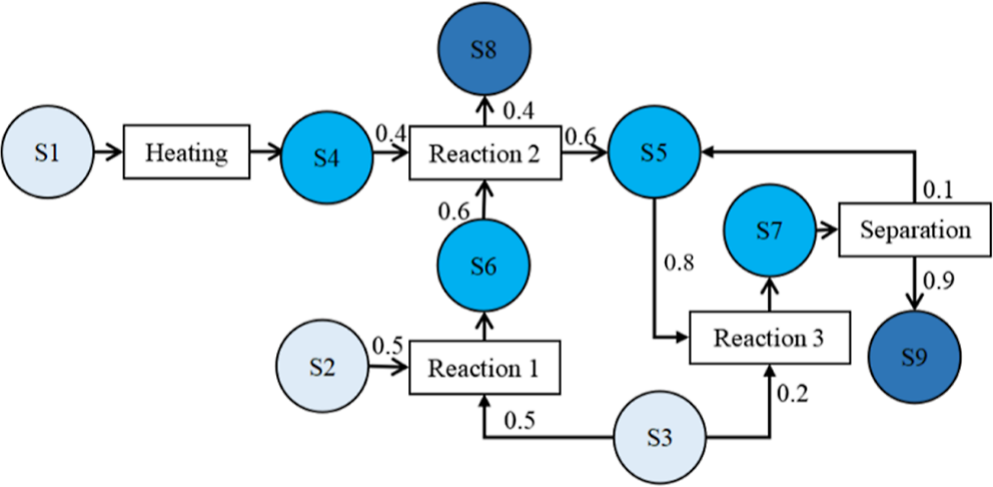
STN representation of a multipurpose batch plant where circles stand for states, rectangles represent tasks, and numbers are proportions of states consumed or produced (ρ_*si*_).

Figure 1 illustrates an STN representation of a typical multipurpose batch plant, where batch splitting, mixing, and recycling are permitted. There are *I* (*i* = 1, 2, 3, ..., I) tasks such as heating, reaction, and separation processed in *J* (*j* = 1, 2, 3, ..., *J*) units. The unit *j* can process **I**_*j*_ tasks. However, it can process at most one task at a time. All materials including raw materials **S**^*R*^, intermediate products **S**^*in*^, and final products **S**^*P*^ are denoted as states. There are *S* (*s* = 1, 2, 3, ..., *S*) states, subject to different storage policies such as finite intermediate storage (FIS), unlimited intermediate storage (UIS), no intermediate storage (NIS), and zero-wait (ZW) policies. The proportion of a state *s* that a task *i* consumes or produces is known and denoted by a parameter ρ_*si*_.

The scheduling problem is stated as follows: Given (a) *J* units, suitable **I**_*j*_ tasks, minimum (*B*_*ij*_^min^) and maximum (*B*_*ij*_^max^) capacities; (b) *S* states, related **I**_***s***_ tasks, initial amount, and storage capacities; (c) production recipes (i.e., the coefficients of processing time for each task, and the consuming or producing fractions of tasks); (d) economic data (e.g., product price, raw material cost, and unit operating cost), scheduling horizon and product demands. It is to determine (a) optimal production schedule including allocation, sequence, timings of tasks in a unit; (b) amount of materials being processed in each unit at each time; (c) inventory profiles of all material states. Assumptions include (i) all parameters are deterministic without batch/unit failures or operational interruptions; (ii) the processing time of a task depends on the batch size (*b*_*ij*_), which is represented by α_*ij*_ + β_*ij*_·*b*_*ij*_; (iii) unlimited feed materials and other resources like utility and manpower; (iv) unlimited storage for raw materials and final products; (v) negligible transfer time between facilities (e.g., units and storage tanks); (vi) setup or changeover time is lumped into processing time; (vii) each state has its dedicated storage.

Two optimization objectives are considered, including maximization of profit over a given scheduling horizon and minimization of makespan to fulfill consumer demands. The detailed problem description can be referred to Li and Floudas.^14^

## 3 Motivating Examples

We create three examples to illustrate the limitations of existing time-grid continuous-time models for scheduling multipurpose batch plants. The STN representations and relevant data are provided in Figures S2, S3, and Tables S1–S5 in the Supporting Information.

### 3.1 Motivating Example 1

**Figure 2:**
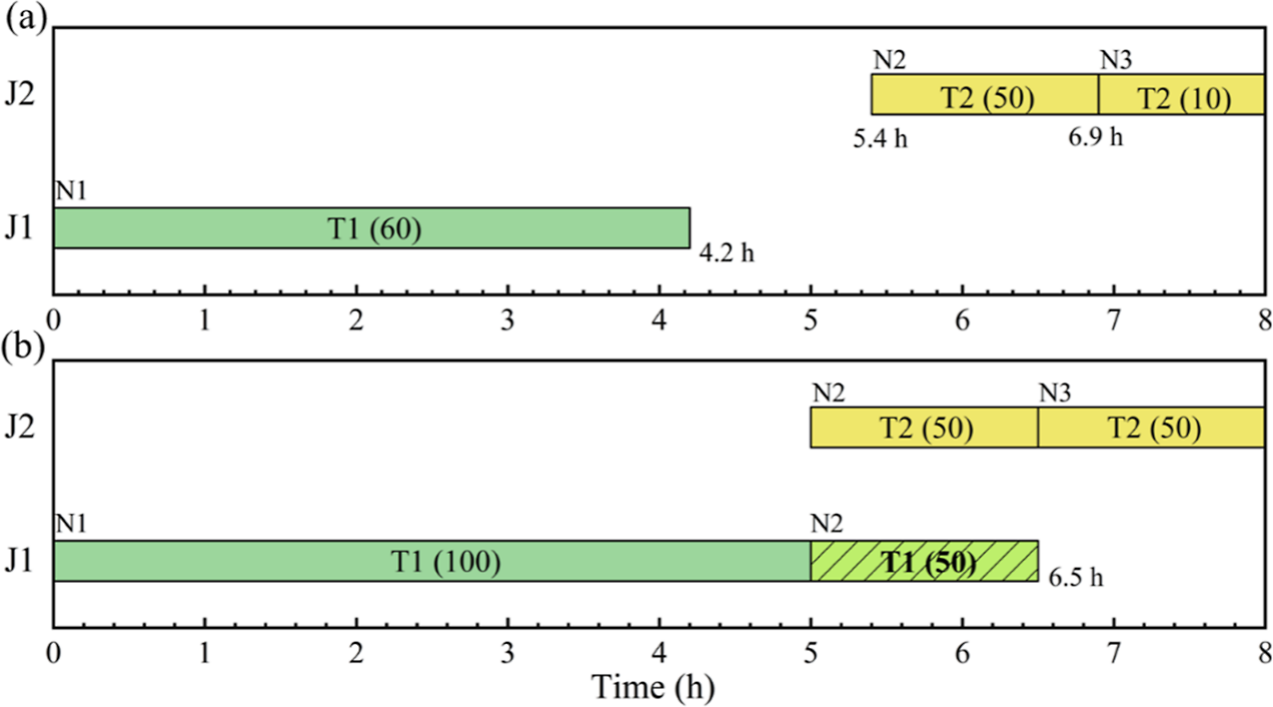
Schedules for motivating example 1. (a) Best schedule with a profit of $300 from models;^10,14,17^ (b)a better schedule with a profit of $500 obtained manually (
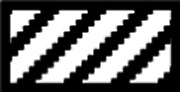
 means amount held in a unit).

In this example, state S2 is produced by task T1 in unit J1 and consumed by task T2 in unit J2. It has a dedicated storage with a maximum capacity of 10 mu (mass units) and no initial inventory. S3 is the final product priced at $5 mu^–1^. The objective is to maximize profit over a given scheduling horizon of 8 h. We use the models of Susarla, Li, and Karimi,^10^ Li and Floudas,^14^ and Vooradi and Shaik^17^ (denoted as **SLK2**, **L&F**, and **V&S**, respectively) to solve this example. These models are excellent representations of existing multiple time-grid continuous-time models. Note that the multiple time-grid model of Mostafaei and Harjunkoski^26^ is not used as it is not suitable for limited storage capacities. The schedule with a maximum profit of $300 obtained from these models is illustrated in Figure 2a, where colors are used to represent different tasks and numbers in parentheses indicate the batch size of tasks (the same for all figures in this paper). However, we can generate another schedule (see Figure 2b) with a maximum profit of $500. It indicates that a better objective value can be obtained than that from all the abovementioned models^10,14,17^ (hence all existing multiple time-grid continuous-time models) due to the fact that in Figure 2b 50 mu of S2 produced in unit J1 is held in J1 at event point N2 from 5 to 6.5 h, while the rest 50 mu of S2 is transferred into unit J2 for further processing. In other words, a batch of S2 is partially transferred to storage or downstream processing units instead of being fully transferred.

### 3.2 Motivating Example 2

**Figure 3:**
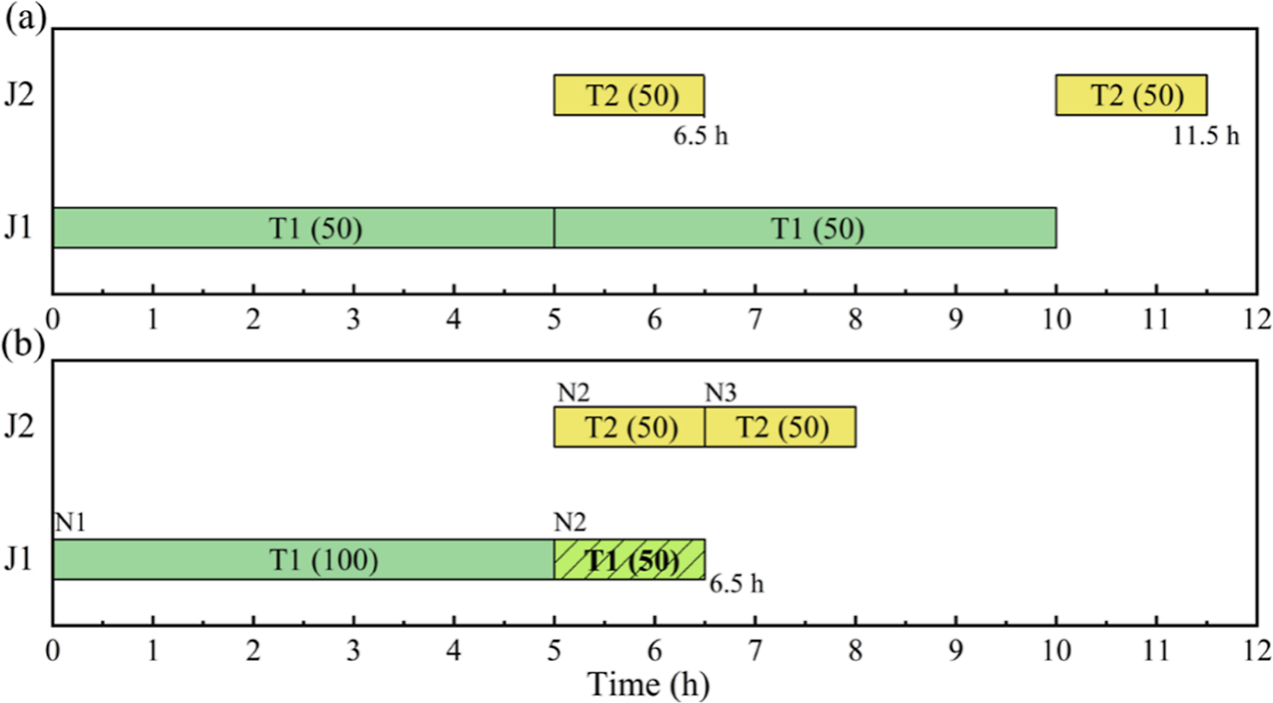
Schedules for motivating example 2. (a) Best schedule with a makespan of 11.5 h from representative models;^10,14,17,32^ (b) a better schedule with a makespan of 8 h obtained manually.

This example is almost the same as the motivating example 1 except with the fixed processing times for tasks, as listed in Table S2. The objective is to minimize makespan with the demand of S3 being 100 mu. Besides the abovementioned representative multiple time-grid continuous-time models,^10,14,17^ a representative discrete-time model^32^ proposed by Velez, Merchan, and Maravelias (referred as **VMM**) is used to solve this example. All these representative models generate a best schedule with a makespan of 11.5 h (see Figure 3a). A better makespan of 8 h can be generated if the batch of S2 produced by task T1 is allowed to be partially transferred to unit J2 and partially held in unit J1 from 5 to 6.5 h (Figure 3b).

### 3.3 Motivating Example 3

**Figure 4:**
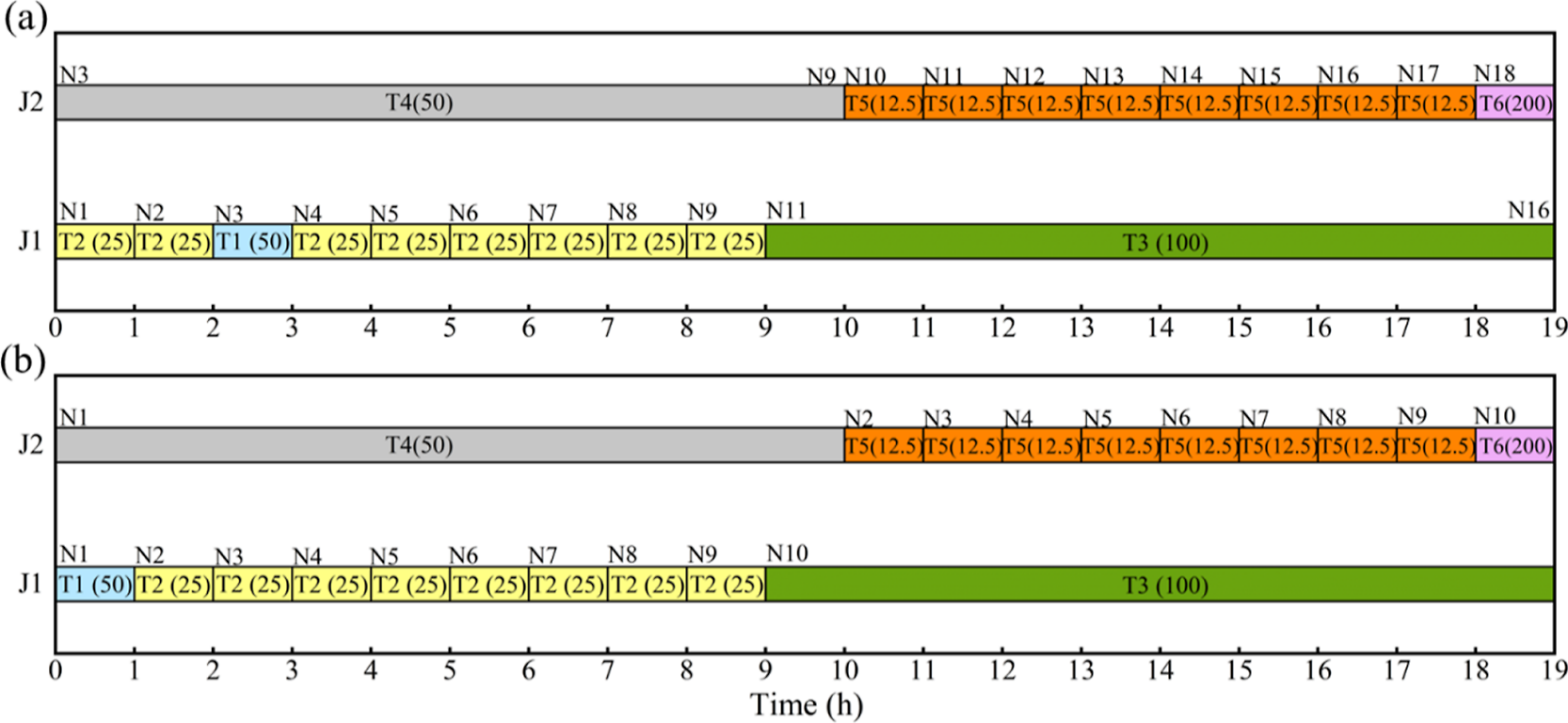
Schedules for motivating example 3. (a) Optimal schedule from model **V&S**(17) using 18 event points and Δ*n* = 6; (b) optimal schedule obtained manually using 10 event points.

In this example, five intermediate states (S1–S5) are subject to FIS with a maximum capacity of 1000 mu and no initial inventory. Demands for products P1 and P2 are 100 and 200 mu, respectively. The objective is to minimize makespan. The optimal makespan of 19 h is generated from **V&S** using 18 event points with Δ*n* = 6, **L&F** using 18 event points with Δ*n* = 0, **SLK2** with 11 slots (12 slot points) and **VMM**. Note that Δ*n* is a parameter denoting the maximum number of event points over which a task is allowed to span. The optimal schedule from **V&S** is illustrated in Figure 4a. However, the same makespan can be generated manually only using 10 event points without allowing a task to span across multiple event points (see Figure 4b). The number of event points required is reduced by 44%, compared to **V&S** and **L&F**.

All these examples motivate us to develop a new mathematical model to address the abovementioned limitations of existing multiple time-grid continuous-time models especially the unit-specific event-based models, which may lead to a significant increase in solution quality.

## 4 Mathematical Formulations

It is of great importance to represent the time horizon before developing a mathematical formulation. Although there are several existing time representations,^3,23,24^ the well-established unit-specific event-based time representation^17−19^ is adopted in this work (see Figure S1 in the Supporting Information) as it is more flexible and efficient (e.g., smaller model size and less computational efforts), compared to other continuous-time representations. It is often more efficient and accurate than the discrete-time variant in handling variable processing times.^26^ For more details about this time representation, the readers can refer to Ierapetritou and Floudas.^12^

### 4.1 Model **M1**

We introduce four-index binary variables  to denote the allocation of tasks to units
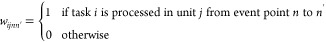
where *n* and *n*′ are event points with *n* ≤ *n*′ ≤ *n* + Δ*n*. The parameter Δ*n* denotes the maximum number of event points that a task is allowed to span over. As discussed before, a state is allowed to be held in a processing unit over consecutive event points after production, if it is neither unstable nor subject to ZW policy. We introduce new binary variables *ys*_*ijn*_ to denote holding operations as follows
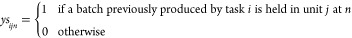


#### 4.1.1 Allocation Constraints

At most, one task is allowed to be processed or temporarily held in a processing unit *j* at a time.
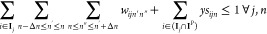
1where set **I**^*P*^ denotes tasks producing states that can be temporarily held in processing units.

#### 4.1.2 Capacity Constraints

The batch size () should be within the minimum (*B*_*ij*_^min^) and maximum (*B*_*ij*_^max^) capacities.
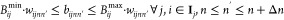
2

#### 4.1.3 Material Balance Constraints

We define positive variables *bs*_*ijn*_ to indicate the amount of a batch from task *i* that is held in unit *j* at event point *n*. If all states produced by task *i* are subject to UIS, it is unnecessary to hold some amount of the batch after production. In other words, *ys*_*ijn*_ = *bs*_*ijn*_ = 0 for UIS. Positive variables *ST*_*sn*_^*M*1^ are defined to denote the total amount of a state *s* stored at event point *n* including the amount stored in storage and processing units. The amount of state *s* stored at the beginning of event point *n* should be equal to the amount stored at the beginning of event point (*n* – 1), plus the amount produced at the end of event point (*n* – 1), minus the amount consumed at the beginning of event point *n* (see eqs 3 and 4).
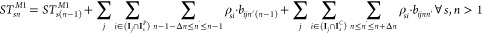
3
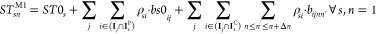
4where *bs*0_*ij*_ is the initial amount of a batch held in unit *j* produced by task *i*, *ST*0_*s*_ is the initial inventory level of state *s*. When ρ_*si*_ > 0, task *i* produces state *s* (called production tasks of *s*, included in set **I**_*s*_^P^), while ρ_*si*_ < 0, task *i* consumes state *s* (called consumption tasks of *s*, included in set **I**_*s*_^C^).

The amount of state *s* held in processing units should not exceed its total amount stored.
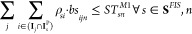
5

#### 4.1.4 Duration Constraints

We define *T*_*jn*_^s^ and *T*_*jn*_^f^ as the start and end times of unit *j* at event point *n*, respectively. Then, the duration of event point *n* on unit *j* must exceed the processing time, as indicated by eq 6.
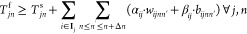
6

#### 4.1.5 Sequencing Constraints for Different Tasks in the Same Unit

Event point (*n* + 1) on unit *j* must start after event point *n* on this unit ends, as given by eq 7.
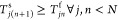
7

To correctly sequence different tasks in different units, we define new continuous variables *T*_*sjn*_ to denote the time when state *s* produced in unit *j* is available at event point *n*. The time when state *s* is available at (*n* + 1) is always after the time when it is available at *n*.
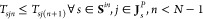
8where set **J**_*s*_^*P*^ denotes the processing units that can process tasks to produce state *s*.

If state *s* is produced in unit *j* at event point *n*, then the time when this state *s* is available at event point *n* should always be after the end time of this event point *n* on unit *j*. In other words, this state *s* is available for storage or consumption only after it is produced.
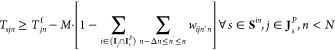
9

If state *s* is produced or temporarily held in unit *j* at event point *n*, then the time when this state *s* is available at *n*, must be before the start time of event point (*n* + 1) on this unit *j*.
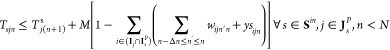
10

#### 4.1.6 Material Transfer

**Figure 5:**
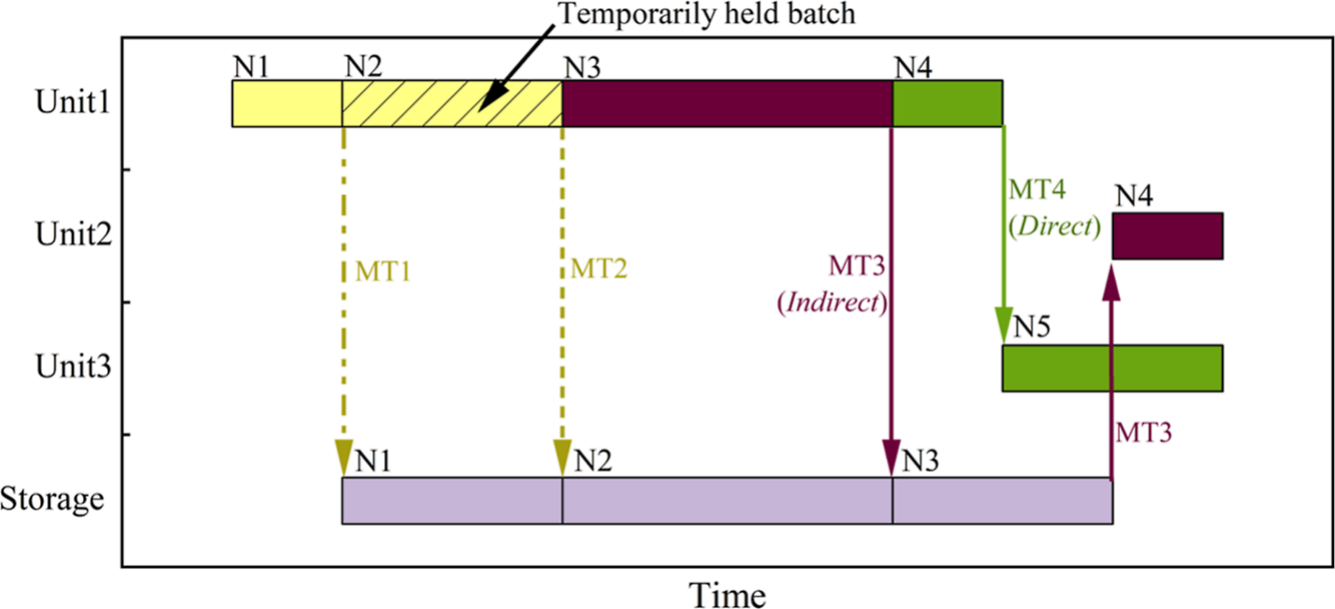
Indirect and direct material transfer.

Material transfer between processing units and storage in batch scheduling has been studied in the literature.^10,16,17,33−39^ Non-instantaneous material transfer,^10,16,17,35^ zero-wait material transfer,^36,37^ and non-zero transfer times^38,39^ are also implicitly or explicitly taken into account. However, the differentiation of these material transfers based on the availability of the storage is not explicitly discussed. In this work, we explicitly introduce the concept of indirect and direct material transfer, which allows us to differentiate material transfer between processing units and storage based on the availability of storage capacity and rigorously sequence and align tasks in different units. As shown in Figure 5, there are four scenarios of material transfer. First, materials can be transferred to storage immediately after production (e.g., material transfer MT1). In scenario 2, materials can be held in the processing units for some duration after production and then transferred to storage (e.g., MT2). If the storage capacity is large enough, materials produced or temporarily held in a unit can be first transferred to storage and then transferred to downstream units for further processing (e.g., MT3). This scenario is called indirect material transfer. If the storage capacity is not large enough, then some materials must be transferred directly to downstream processing units (e.g., MT4), which is direct material transfer. We generally classify material transfer as indirect and direct material transfer. Note that indirect and direct material transfer can take place simultaneously.

##### 4.1.6.1 Indirect Material Transfer

In this scheme, the storage capacity is usually large enough. A state subject to UIS policy is always indirectly transferred to downstream processing units. For a state with FIS policy, indirect material transfer of the state works only when there is enough storage capacity. To model this indirect material transfer scheme, we define new binary variables 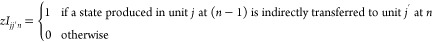


If indirect material transfer happens from unit *j* to unit *j*′ at event point (*n* + 1), the start time of event point (*n* + 1) on unit *j*′ should exceed the end time of event point *n* on unit *j*.
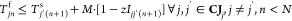
11where set **CJ**_*j*_ denotes units consuming a state produced in unit *j*, that is, , in which set **J**_*s*_^*C*^ denotes the processing units that can process tasks to consume state *s*.

We also define continuous variables  to denote the amount of a state through indirect transfer between unit *j* and unit *j*′. Specifically, the state is produced by a task *i* in unit *j* at event point (*n* – 1) and then indirectly transferred to a downstream unit *j*^′^ in which task *i*′ takes place and consumes the transferred state at event point *n*. The total amount of state *s* required by downstream tasks at event point *n* should not exceed the amount stored at the previous event point (*n* – 1) plus the amount through indirect transfer.
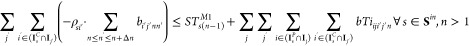
12
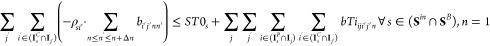
13where set **S**^*B*^ denotes the states that are initially held in the processing units performing production tasks of the state, that is, .

The amount of state *s* produced by task *i* in unit *j* through indirect material transfer at event point *n* should not exceed the batch size produced by this task at the previous event point (*n* – 1). When there is indirect material transfer from a unit *j* to a downstream unit *j*′ at the first event point, the transferred material comes from the batch of the state that is initially held in unit *j* (i.e., *bs*0_*ij*_ > 0).
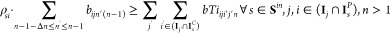
14
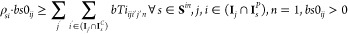
15

Similarly, the amount of state *s* indirectly transferred to a downstream unit *j*^′^ where task *i*′ taking place at event point *n* should not exceed the amount required by this task *i*′.
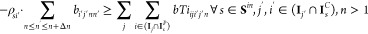
16
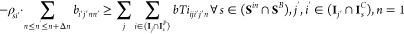
17

When there is no indirect material transfer between two processing units, the amount through the indirect material transfer should be zero, which is ensured by eqs 18 and 19.
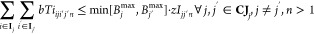
18
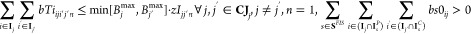
19where  and .

The abovementioned constraints are used to monitor indirect material transfer as well as the correct timing sequence between unit *j* and unit *j*^′^ at event point (*n* + 1). The indirect material transfer is not monitored from unit *j* to unit *j*^′^ at event point (*n* + 2) or higher event points. If a state produced in unit *j* at event point *n* is consumed by a task in unit *j*′ taking place at event point (*n* + 2) or higher event points, then the unit *j*^′^ that consumes state *s* at event point (*n* + 2) or higher event points should always start after the state is available on unit *j* at event point *n* to avoid storage violation. This logic condition is ensured by eqs 20 and 8.
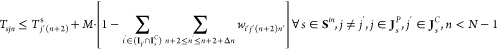
20

##### 4.1.6.2 Direct Material Transfer

For a state subject to FIS policy, if there is no storage space, this state cannot be transferred to a dedicated storage tank. Instead, it must be transferred directly from unit *j* to unit *j*^′^ where it is consumed. To model direct material transfer, binary variables  are introduced as follows
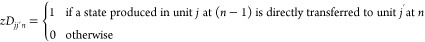


If direct material transfer takes place from unit *j* to *j*^′^ at (*n* + 1), unit *j*^′^ at event point *n* must finish before unit *j* finishes at the same *n* to avoid overlapping on *j*^′^, as indicated in eq 21.
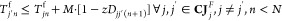
21where .

We define continuous variables  to denote the amount of a state directly transferred from unit *j* where it is produced by a task *i* or temporarily held at event point (*n* – 1) to a downstream unit *j*^′^ where it is consumed by a task *i*^′^ at event point *n*. Direct material transfer of a state from its producing task *i* on unit *j* at event (*n* – 1) to the consuming task *i*′ on unit *j*′ at event point *n* should be activated when there is no sufficient storage space to settle the amount of the state provided (produced or temporarily held in unit *j*) at event point (*n* – 1). Equation 22 indicates when the provided amount of one state plus the excess amount of the state at event point (*n* – 1) exceeds the maximum storage capacity, direct material transfer should be non-zero. Equation 23 enforces the inequality at the first event point.
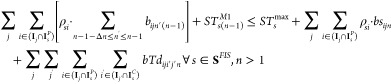
22
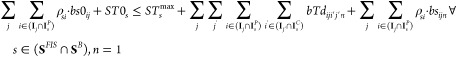
23

Similar to indirect material transfer, eqs 24 and 25 enforce the amount of a state through direct material transfer and its remaining amount held in the processing unit at event point *n* must not exceed the amount of this state provided by task *i* on unit *j* at the previous event point (*n* – 1).
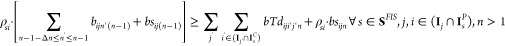
24
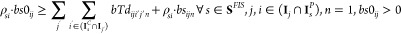
25

The amount of state *s* through direct material transfer to unit *j*^′^ where task *i*^′^ takes place at event point *n* should not exceed the amount of this state consumed by task *i*^′^ at event point *n*.
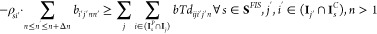
26
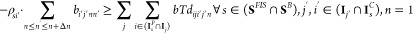
27

When there is no direct material transfer between two related processing units, the amount through this direct material transfer should be zero.
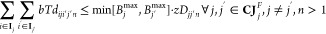
28
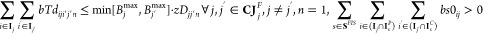
29

#### 4.1.7 Additional Sequence Constraints for FIS Policy

**Figure 6:**
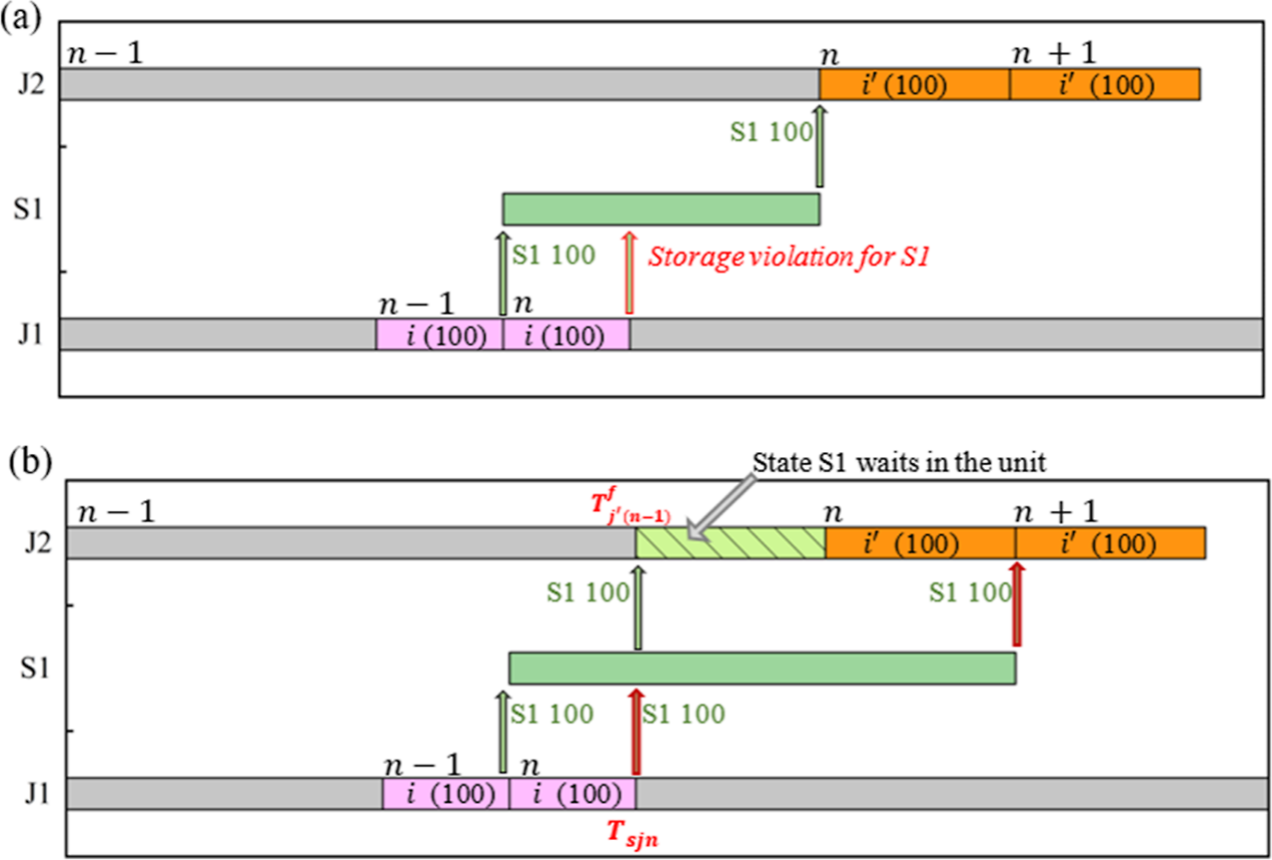
Illustration of the meaning of eq 30 where the maximum storage capacity of state S1 is 100 mu. (a) An infeasible schedule without eq 30 due to real-time storage violation; (b) a feasible solution generated with eq 30 (gray shading means the unit is processing a task not related to state S1).

To avoid storage violation in real time, constraint 30 is also imposed to ensure that the time when state *s* produced in unit *j* is available at event point *n* must be after the finish time of event point (*n* – 1) on unit *j*^′^ where a task consumes state *s* at event point *n*. This is to ensure that when state *s* is available, there is enough space to hold this state, as illustrated in Figure 6. In Figure 6a, when *S*1 is available at the end of event point *n*, there is no enough storage space for *S*1 because eq 30 is not imposed. However, if eq 30 is imposed, when *S*1 is available at the end of event point *n*, it can be stored in the dedicated storage as *S*1 stored in the storage tank is transferred to unit *J*2 for processing, as shown in Figure 6b.
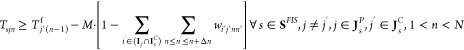
30

An upper bound for the excess amount of state *s* stored is enforced by eq 31, which means the excess amount of state *s* stored at a time must not exceed the maximum storage capacity plus the maximum allowable holding amount in all processing units.
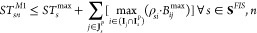
31

#### 4.1.8 Allowing Processing Units to Store Materials

The amount of a state produced by task *i* that is temporarily held in unit *j* at event point *n* must be zero if the binary variable *ys*_*ijn*_ = 0.
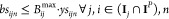
32

The amount of a state held in a processing unit at the first event point must be smaller than the initial holding amount, as ascertained by eq 33.
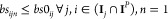
33

#### 4.1.9 Tightening Constraints

Some new tightening constraints are introduced to improve the performance of the proposed model. Constraints (34)–(37) relate  and *ys*_*ijn*_ with . Equation 34 ensures if unit *j*^′^ processes a task consuming intermediate state *s*, and indirect material transfer happens between units *j* and *j*^′^ at event point (*n* + 1), unit *j* must process a task producing *s* or temporarily hold *s* at event point *n*.
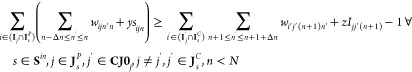
34where set **CJ**0_*j*_ includes units *j*^′^∈**CJ**_*j*_ except those in which multiple states produced in *j* are consumed by a task in *j*^′^ or multiple states consumed by tasks in *j*^′^ are produced by a task in *j*.

Similarly, if unit *j* processes a task producing state *s* at event point *n*, and there is indirect material transfer between units *j* and *j*^′^ at event point (*n* + 1), then unit *j*′ must process a related consumption task starting at event point (*n* + 1) according to constraint (35).
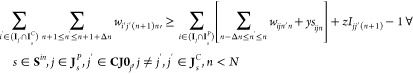
35

When direct material transfer between two units takes place (i.e., ), indirect material transfer should also take place between these two units without loss of generality.
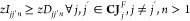
36

When indirect material transfer takes place between units *j* and *j*^′^ in set **CJ**_*j*_ at event point *n*, unit *j* must process or hold a state at event point (*n* – 1) and *j*^′^ must process a task at event point *n*.
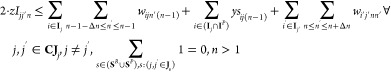
37

#### 4.1.10 Bounds and Fixing

The start and end times of unit *j* at event point *n* must be smaller than the time horizon (*H*), as ascertained by eqs 38 and 39. The parameter *H* would be replaced by *M* while addressing minimization of makespan, as the scheduling horizon (*H*) may be unavailable.
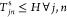
38
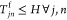
39

If all states produced by task *i* are subject to UIS policy, *ys*_*ijn*_ and *bs*_*ijn*_ should be zero.
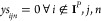
40
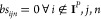
41

While eq 42 imposes task *i* in unit *j* starting at event point *n* cannot finish at event point *n*^′^ when *n*′ < *n*, eq 43 ensures that a task must be processed in its suitable unit.
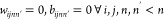
42
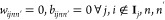
43

If there is no initial amount of a state held in a processing unit, the variables related to temporarily holding materials and material transfer are fixed as zero at the first event point.
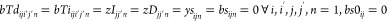
44

#### 4.1.11 Objective Functions

As already discussed, two objective functions are considered. While constraint (45) is used for maximization of profit, constraint (46) is used for minimization of makespan.
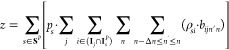
45
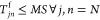
46

In minimization of makespan, it should be also ensured that the total demand is satisfied.
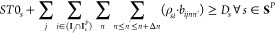
47

The tightening constraint 48 is added for minimization of makespan only.
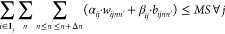
48

Finally, (49) and (50) denote all the continuous and binary variables of the model.
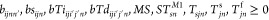
49
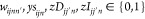
50

We complete the mathematical model **M1**, which consists of constraints (1–45) and (49, 50) for maximization of profit, and (1–44) and (46–50) for minimization of makespan.

### 4.2 Model **M2**

In this model **M2**, the amount stored in dedicated storage and processing units is explicitly separated, which is different from model **M1**. As many constraints in this model are the same as those in model **M1**, the constraints that are different from model **M1** are discussed in detail below.

We define new variables *ST*_*sn*_^*M*2^ to denote the amount of state *s* stored in its dedicated storage tank at event point *n*. This amount denoted by *ST*_*sn*_^*M*2^ is the amount denoted by *ST*_*sn*_^*M*1^ in model **M1** minus the amount stored in suitable processing units. Then, the material balance changes to the following eqs 51 and 52 using *ST*_*sn*_^*M*2^.
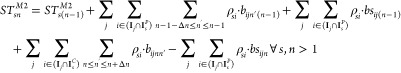
51
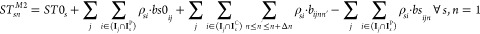
52

Similar to eq 12, the total amount of states required by downstream tasks at event point *n* should not exceed the amount stored at the previous event point (i.e., *ST*_*s*(*n*–__1)_^*M*2^) plus the amount through indirect transfer.
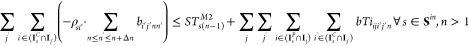
53

The amount of state *s* through indirect material transfer at event point *n* should not exceed its available amount produced from task *i* or held in the processing unit at event point (*n* – 1).
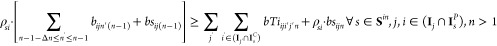
54
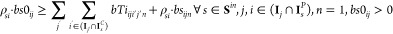
55

Equation 56 indicates when the provided amount of one state plus the inventory level of the state at event point (*n* – 1) exceeds the maximum storage capacity, direct material transfer should be non-zero.
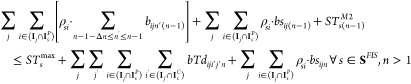
56
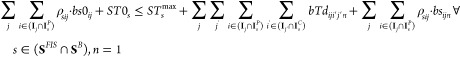
57

The inventory level of state *s* with FIS must not exceed the maximum storage capacity.
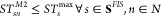
58

We define *T*_*sn*_^*M*2^ to denote the time when a state *s* is available at event point *n* instead of using *T*_*sjn*_. This is because the computational performance becomes worse when *T*_*sjn*_ is used, as demonstrated in the Supporting Information through comparing the performance of this model **M2** with model **M3** using *T*_*sjn*_ presented in Section S8 of Supporting Information. The difference between *T*_*sn*_^*M*2^ and *T*_*sjn*_ is depicted in Figure S16 of the Supporting Information.

We require that the time when a state *s* is available at event point (*n* + 1) should be always after its available time at event point *n*, as indicated by eq 59.
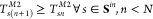
59

A state *s* is available for transfer only after it is produced, which is enforced by eq 60.
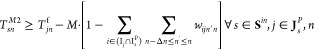
60

Similar to constraint (20), if a state produced in unit *j* at event point *n* is consumed by a task in unit *j*′ taking place at event point (*n* + 2) or higher event points, the unit *j*^′^ that consumes state *s* at (*n*+ 2) or higher event points should always start after the state is available on unit *j* at *n* to avoid storage violation. This logic condition is ensured by eqs 61 and 59.
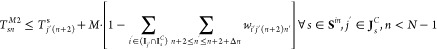
61

Equations 62–64 are used to ensure that a unit *j* that processes or temporarily holds one batch of a production task for state *s* at event point *n* should finish after the end time of unit *j*^′^ at the previous event point (*n* – 1), where a task consumes the state *s* at event point *n*, to avoid real-time storage violation. Here, *tt*_*sn*_^*M*2^ are intermediate variables. Handled by eqs 62 and 63, the unit *j*′ consumes or pre-processes a state *s* before its production in unit *j* at the same event point to avoid the inventory level of state *s* after production in units at *n* beyond the maximum storage capacity.
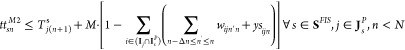
62
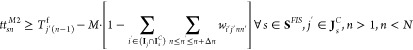
63
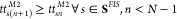
64

The available time of any state at the last event point should be smaller than makespan while addressing the minimization of makespan.
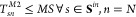
65

Some upper bounds are also imposed to this model **M2** as better computational performance is obtained based on our computational experience. Batch size of tasks and amount for one batch of materials held in a suitable processing unit should be always smaller than the maximum unit capacity, as handled by eqs 66 and 67.
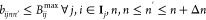
66
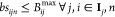
67

Equations 68 and 69 express that the amount of state *s* through indirect or direct material transfer cannot exceed its maximum production amount from a task *i* at unit *j* and the maximum consumption amount by a task *i*′ at unit *j*′.
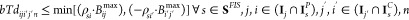
68
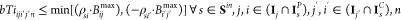
69

All continuous variables of model **M2** are denoted by eq 70.
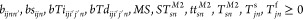
70

The mathematical model **M2** consists of constraints (1, 2), (6, 7), (11), (13), (16–19), (21), (24–29), (32–45), (50–68), and (70) for maximization of profit, and (1, 2), (6, 7), (11), (13), (16–19), (21), (32–45), (32–44), (46–48), and (50–70) for minimization of makespan. The complete model **M2** is provided in Section S7 of the Supporting Information.

### 4.3 Extensions

The proposed model **M1** can be extended to address the following situations like variable recipes, and states subject to NIS and ZW policies. We use model **M1** for this extension due to its better computational performance than **M2** (see Section 5).

#### 4.3.1 Variable Recipes

The proposed model can be extended to handle schedule problems with variable recipe of a task,^40^ which indicates flexible proportions of output or input materials are used in a task. Such features have been addressed in the literature for some industrial applications.^41−43^ We define two new continuous variables  and *bsv*_*sijn*_ to denote the amount of state *s* produced by task *i* in unit *j* from *n* to *n*^′^ and the amount of state *s* in a batch produced by task *i* and held in unit *j* at *n*, respectively. Set **I**^*V*^ indicates tasks with variable recipes. The amount of state *s* produced by task *i*∈**I**^*V*^ is bounded by maximum and minimum fractions.
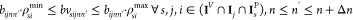
71

The total amount of states produced by task *i*∈**I**^*V*^ should equal the batch size of the task.
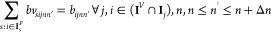
72

Amounts of one state in one batch, produced by task *i* at previous events and temporarily held in the processing unit *j* at event *n*, are bounded by the maximum and minimum fractions.
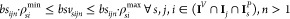
73
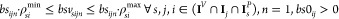
74

The amount in a batch held in unit *j* at event point *n* is calculated by eqs 75 and 76.
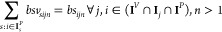
75
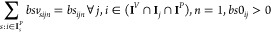
76

For task *i*∈**I**^*V*^, the amount of a state in a batch produced by task *i* and temporarily held in unit *j* at event point (*n* + 1) is no more than its amount provided by the task *i* at *n*.
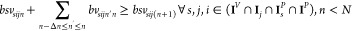
77

To handle variable production recipes for some tasks, constraints concerning material balance and material transfer should be modified. Specifically, variables () and (ρ_*si*_·*bs*_*ijn*_) for task *i*∈**I**^*V*^ should be replaced by  and *bsv*_*sijn*_, respectively.

#### 4.3.2 NIS and ZW Policies

A state with NIS policy can be either temporarily held in the processing unit or directly transferred into downstream processing units after its production, as there is no storage tank.
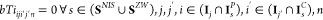
78
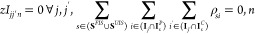
79

Equations 78 and 79 are also true for a state with ZW policy as the state must be transferred to and processed immediately in the downstream processing units after production.
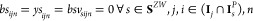
80

When task *i* in unit *j* produces a state subject to ZW policy at event point *n*, the duration of this event point *n* over unit *j* must be exactly equal to the processing time of this task *i* at event point *n*, which is ensured by eqs 6 and 81. Sets **I**^*Pzw*^ and **J**^*Pzw*^ denote tasks in corresponding units that are able to produce a state with ZW policy, respectively.
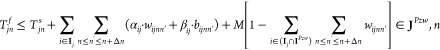
81

Equation 82 enforces the end time of the producing tasks is earlier than the start time of the consuming tasks for direct transfer of a state subject to NIS or ZW policy.
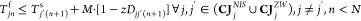
82where  and .

For a state with ZW policy, a task consuming this state at event point (*n* + 1) should immediately start after this state is produced at *n*, as pre-processing unit wait is not permitted.
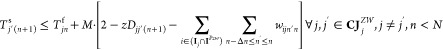
83
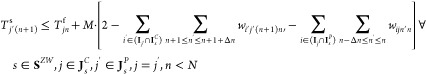
84

Equation 85 expresses that for a state subject to NIS or ZW policy, materials produced by a task or held at event point (*n* – 1) should be consumed, or partially held in the processing units at event point *n*. Given that a state for ZW cannot be held in processing units, eq 86 is valid only if the state subject to NIS is initially stored at one processing unit.
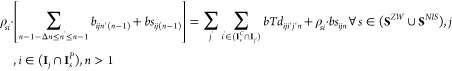
85
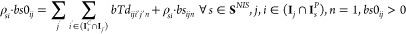
86

For a state subject to NIS or ZW policy, the amount of this state consumed must be from direct material transfer.
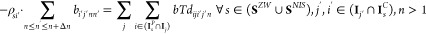
87
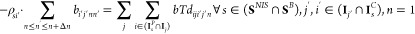
88

For a state subject to ZW policy, excess amounts of this state must be zero.
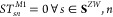
89
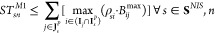
90

Equations 91 and 92 enforce that a task producing states subject to ZW cannot occur at the last event.
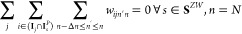
91
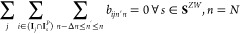
92

Equation 93 expresses that a state subject to ZW or NIS policy must be directly transferred to a downstream processing unit or partially held in the processing unit *j* for NIS after production. Similarly, materials used by consuming tasks at event *n* must be transferred directly from production tasks finished at the previous event (*n* – 1), given by eq 94.
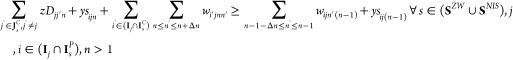
93
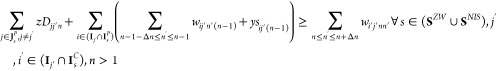
94

The complete model with all extensions is denoted as **EM1**, which is provided in Section S9 of the Supporting Information. Note that models **M1–M2** are developed for scheduling multipurpose batch plants for FIS and UIS, while the extended model **EM1** can solve problems with variable proportion of states produced/consumed and four types of storage policies (e.g., FIS, NIS, UIS, and ZW). It should also be noted that although many sets are defined, most of them can be automatically determined based on sets **S**^*R*^, **S**^*in*^, **S**^*P*^, **S**^*FIS*^, **S**^*UIS*^, **S**^*NIS*^, **S**^*ZW*^, **I**^*V*^, **I**_*j*_ and the parameter ρ_*si*_.

## 5 Computational Studies

To evaluate the performance of the proposed mathematical models **M1–M2**, we solve 16 examples including three motivating examples, 11 benchmark examples from the literature,^14,17^ a variant of benchmark example 3 (denoted as example 12), and the Kallrath example.^43^ Relevant data, STN representations, and values of *M* in big-M constraints for all examples are provided in the Supporting Information. It should be noted that we only compare the performance of our models with model **V&S**(17) when we solve the benchmarking examples and the Kallrath example, as **V&S** usually outperforms other continuous-time models.^17^ Although the multiple-grid slot-based model from Mostafaei and Harjunkoski^26^ eliminates some big-M constraints, it is not suitable for limited storage capacities and often leads to much greater number of event points. Detailed comparison of our models with Shaik and Floudas,^13^ Li and Floudas,^14^ Susarla, Li, and Karimi,^10^ and Mostafaei and Harjunkoski^26^ will be presented in our next contribution.

All models are run using the CPLEX 12.6.3/GAMS 24.6.1 on a desktop computer with AMD Ryzen 9 3900X 3.8 GHz and 48 GB RAM running Windows 10. The maximum computational time is set as 1 h for the motivating examples and benchmark examples, while it is set as 40,000 s for the industrial-scale Kallrath example. The relative optimality gap for all examples is set as zero.

### 5.1 Motivating Examples

We revisit the motivating examples 1–3. The computational results are provided in Table 1. From Table 1, it can be observed that our models can lead to a 66.7% higher profit ($500) for motivating example 1 than other representative models like **V&S**, **L&F**, and **SLK2** ($300) due to the added flexibility allowing materials produced to be held in the processing units over consecutive event points to share storage burden. For motivating example 2, our models lead to a 30.4% smaller makespan (8 vs 11.5 h) than the existing continuous-^10,14,17^ and discrete-time^32^ models. For motivating example 3, our models generate the optimal makespan (19 h) using 10 event points compared to **V&S** with 18 event points and Δ*n* = 6, resulting in a reduction in the numbers of event points by 44% (10 vs 18) and binary variables by 84% (114 vs 732). In addition, **V&S** requires more than 1 h to generate the optimal solution using 18 event points with Δ*n* = 1 and 2. As a result, the total CPU time required to generate the optimal makespan of 19 h using our models is significantly decreased by 3 orders of magnitude according to the iterative procedure. This is mainly because **V&S** imposes if task *i* produces state *s* at event point *n*, it must finish after a task *i*^′^ starts to consume this state *s*∈**S**^*FIS*^ at *n* and before a task *i*″ starts to consume this state *s*∈**S**^*in*^ at (*n* + 2), regardless of whether task *i*^′^ or *i*^″^ actually takes place at *n* or (*n* + 2), which is overcome by eqs 9, 10, 20, and 30 in **M1** and eqs 59–64 in **M2**.

**Table 1:** Computational Results for Motivating Examples^a^

model	event points/H^b^	RMILP	MILP	CPU time (s)	binary variables	continuous variables	constraints
Motivating Example 1 (*H* = 8 h)							
**V&S**	3	500.00	300.00	0.05	12	32	66
**L&**F	3	500.00	300.00	0.13	6	29	41
**SLK2**	4	500.00	300.00	0.05	12	81	94
**M1**	3	500.00	**500.00**	0.05	12	37	72
**M2**	3	500.00	**500.00**	0.05	12	40	72
Motivating Example 2 (*D*_*S*3_ = 100 mu)							
**V&S**	3	5.00	11.5	0.02	12	32	68
**L&F**	3	5.00	11.5	0.02	6	29	43
**SLK2**	4	5.00	11.5	0.02	12	83	99
**VMM**	100^c^	5.00	11.5	0.23	404	1006	2822
**M1**	3	8.00	**8**	0.02	12	37	76
**M2**	3	8.00	**8**	0.03	12	41	81
Motivating Example 3 (*D*_*P*1_ = 100 mu, *D*_*P*2_ = 200 mu)							
**V&S**	10	27.00	27.00	0.05	114	350	898
	18	19.00	25.00	44.5	210	638	1666
	18 (Δ*n* = 1)	19.00	24.00^d^	3600^f^	312	740	2074
	18 (Δ*n* = 2)	19.00	23.00^e^	3600^f^	408	836	2362
	18 (Δ*n* = 5)	19.00	20.00	459	660	1088	3118
	18 (Δ*n* = 6)	19.00	19.00	20.7	732	1160	3334
**L&F**	16	19.00	21.00	2.52	96	418	724
	17	19.00	20.00	2.02	102	444	770
	18	19.00	19.00	0.41	108	470	816
**SLK2**	11	28.00	28.00	0.05	80	534	733
	12	19.00	19.00	0.19	88	584	805
**VMM**	100^g^	10.00	19.00	0.86	612	1415	3256
**M1**	**10**	19.00	19.00	0.02	114	479	803
**M2**	**10**	19.00	19.00	0.03	114	403	856

a **V&S**: Vooradi and Shaik;^17^**L&F**: Li and Floudas;^14^**SLK2**: Susarla, Li, and Karimi;^10^**VMM**: Velez, Merchan, and Maravelias.^32^

b Event points for continuous-time models and H for **VMM**. Step size.

c δ = 0.5. Relative gap.

d 3.33%.

e 4.35%.

f Resource limit reached. Step size.

g δ = 1.

### 5.2 Benchmark Examples

The computational results from solving 11 benchmark examples and a variant example of example 3 (i.e., example 12)^14^ are presented in Tables 2–5. The model statistics (e.g., the number of binary variables, continuous variables, and constraints) for **V&S** are different from those reported in Vooradi and Shaik^17^ as some binary variables are not fixed based on STN. If we fix them, we do get the same model statistics. We do not present results with tiny computational efforts in these tables. The complete results are provided in Section S3 of the Supporting Information.

**Table 2:** Computational Results for Benchmark Examples for Minimization of Makespan (UIS)^a^

model	event points	RMILP	MILP (h)	CPU time (s)	binary variables	continuous variables	constraints
Example 1a (*D*_*S*4_ = 2000 mu)							
**V&S**	14	24.24	27.88	833.2	122	319	667
	15	24.24	27.88^b^	3600	131	342	716
**M1**	14	24.24	27.88	**133.7**	122	359	868
	15	24.24	27.88	**2578**	131	385	933
**M2**	14	24.24	27.88	**165.3**	122	348	812
	15	24.24	27.88^c^	3600	131	373	872
Example 1b (*D*_*S*4_ = 4000 mu)							
**V&S**	23	48.47	52.07	1164	203	526	1108
**M1**	23	48.47	52.07	**597**	203	593	1453
**M2**	23	48.47	52.07	**449.4**	203	573	1352

a **V&S**: Vooradi and Shaik;^17^ relative gap.

b 4.64%.

c 0.47%.

**Table 3:** Computational Results for Benchmark Examples for Maximization of Profit (UIS)^a^

model	event points	RMILP	MILP ($)	CPU time (s)	binary variables	continuous variables	constraints
Example 1d (*H* = 16 h)							
**V&S**	9	6601.65	5038.05	12.8	77	204	417
	10	6601.65	5038.05	353.6	86	227	466
**M1**	9	6601.65	5038.05	4.7	77	229	533
	10	6601.65	5038.05	**66.9**	86	255	598
**M2**	9	6601.65	5038.05	5.3	77	223	500
	10	6601.65	5038.05	**84.1**	86	248	560
Example 2d (*H* = 16 h)							
**V&S**	8	4291.68	3738.38	10.99	134	364	772
	9	4438.96	3738.38	736.0	152	411	878
**M1**	8	4291.68	3738.38	10.88	120	540	876
	9	4438.96	3738.38	**417.9**	136	613	1000
**M2**	8	4291.68	3738.38	10.56	120	334	790
	9	4438.96	3738.38	**527.0**	136	377	899
Example 3a (*H* = 8 h)							
**V&S**	5	2100.00	1583.44	1.13	123	309	682
	6	2750.96	1583.44	721.1	151	374	842
**M1**	5	2100.00	1583.44	0.80	115	469	815
	6	2750.96	1583.44	**42.0**	141	576	1016
**M2**	5	2100.00	1583.44	0.83	115	296	762
	6	2750.96	1583.44	**72.6**	141	358	943
Example 3b (*H* = 10 h)							
**V&S**	7	3369.69	2358.20	514.9	179	439	1002
	8	3618.64	2358.20^b^	3600	207	504	1162
**M1**	7	3369.69	2358.20	**70.1**	167	683	1217
	8	3618.64	2358.20	**2476**	193	790	1418
**M2**	7	3369.69	2358.20	**114.8**	167	420	1124
	8	3618.64	2358.20^c^	3600	193	482	1305
Example 3d (*H* = 16 h)							
**V&**S	10	5225.86	4262.80	49.53	263	634	1482
	11	5644.59	4262.80^d^	3600	291	699	1642
**M1**	10	5225.86	4262.80	42.25	245	1004	1820
	11	5644.59	4262.80	**2067**	271	1111	2021
**M2**	10	5225.86	4262.80	62.02	245	606	1667
	11	5644.59	4262.80	**2125**	271	668	1848

a **V&S**: Vooradi and Shaik;^17^ relative gap.

b 0.16%.

c 0.09%.

d 0.09%.

**Table 4:** Computational Results for Benchmark Examples with Maximization of Profit (FIS)^a^

model	event points	RMILP	MILP ($)	CPU time (s)	binary variables	continuous variables	constraints
Example 3b (*H* = 10 h)							
**V&S**	7	3369.69	2358.20	950.7	383	553	1794
	8	3618.64	2358.20^b^	3600	445	637	2086
**M1**	7	3369.69	2358.20	**339.5**	311	1097	1855
	8	3618.64	2358.20^c^	3600	361	1273	2164
**M2**	7	3369.69	2358.20	**420.5**	311	630	1789
	8	3618.64	2358.20^d^	3600	361	727	2084
Example 3d (*H* = 16 h)							
**V&S**	10	5225.86	4262.80	98.31	569	805	2670
	11	5644.59	4262.80^e^	3600	631	889	2962
**M1**	10	5225.86	4262.80	**83.63**	461	1625	2782
	11	5644.59	4262.80^f^	3600	511	1801	3091
**M2**	10	5225.86	4262.80	**69.23**	461	921	2674
	11	5644.59	4262.80^g^	3600	511	1018	2969
Example 6 (*H* = 9 h)							
**V&S**	5	300.00	180.00	0.17	97	160	425
	5 (Δ*n* = 1)	300.00	**210.00**	0.16	117	180	505
	6	360.00	180.00	0.28	120	194	525
**M1**	**5**	300.00	**210.00**	0.05	89	194	509
**M2**	**5**	300.00	**210.00**	0.06	89	196	493
Example 8 (*H* = 10 h)							
**V&S**	6	400.00	200.13	0.38	84	149	432
	6 (Δ*n* = 1)	400.00	200.13	1.27	104	169	512
	6 (Δ*n* = 2)	400.00	300.00	0.38	120	185	560
	6 (Δ*n* = 3)	400.00	400.00	0.16	132	197	596
	7	500.00	200.13	0.61	100	175	514
**M1**	**6**	400.00	**400.00**	0.05	**79**	185	536
**M2**	**6**	400.00	**400.00**	0.14	**79**	209	536
Example 9 (*H* = 10 h)							
**V&S**	10	400.00	200.13	10.81	148	253	760
	10 (Δ*n* = 1)	400.00	200.13	173.9	184	289	904
	10 (Δ*n* = 2)	400.00	200.13	485.2	216	321	1000
	10 (Δ*n* = 3)	400.00	200.13	2020	244	349	1084
	10 (Δ*n* = 4)	400.00	250.00	127.6	268	373	1156
	10 (Δ*n* = 5)	400.00	300.00	113.6	288	393	1216
	10 (Δ*n* = 6)	400.00	350.00	9.86	304	409	1264
	10 (Δ*n* = 7)	400.00	400.00	1.77	316	421	1300
	11	450.00	200.13	19.7	164	279	842
**M1**	**10**	400.00	**400.00**	**0.16**	**139**	317	956
**M2**	**10**	400.00	**400.00**	**0.59**	**139**	357	956
Example 12 (*H* = 12 h)							
**V&S**	10	1795.48	989.03	2096	569	805	2670
	11	2208.35	989.03^h^	3600	631	889	2962
**M1**	10	1795.48	**1184.48**	**316.8**	461	1625	2782
	11	2208.35	**1201.39**^i^	3600	511	1801	3091
**M2**	10	1795.48	**1184.48**	**189.9**	461	921	2674
	11	2208.35	**1201.39**^j^	3600	511	1018	2969

a **V&S**: Vooradi and Shaik;^17^ relative gap.

b 1.99%.

c 0.15%.

d 0.15%.

e 1.41%.

f 0.83%.

g 3.11%.

h 3.23%.

i 5.74%.

j 5.08%.

**Table 5:** Computational Results for Benchmark Examples with Minimization of Makespan (FIS)^a^

model	event points	RMILP	MILP (h)	CPU time (s)	binary variables	continuous variables	constraints
Example 1a (*D*_*S*4_ = 2000 mu)							
**V&S**	14	24.24	27.88^b^	3600	226	371	1031
**M1**	14	24.24	27.88	**1969**	213	450	1243
**M2**	14	24.24	27.88^c^	3600	213	465	1210
Example 1b (*D*_*S*4_ = 4000 mu)							
**V&S**	24	48.47	52.07^d^	3600	396	641	1801
**M1**	24	48.47	52.07^e^	3600	373	780	2183
**M2**	24	48.47	52.07^f^	3600	373	805	2120
Example 2b (*D*_*S*8_ = 500 mu, *D*_*S*9_ = 400 mu)							
**V&S**	21	47.46	47.74^g^	3600	768	1255	3859
	21 (Δ*n* = 1)	47.38	47.69^h^	3600	928	1415	4499
	21 (Δ*n* = 2)	47.38	47.68^i^	3600	1080	1567	4955
	22	47.46	47.73^j^	3600	806	1316	4050
**M1**	**21**	47.38	**47.68**	**82.0**	**648**	2449	3791
**M2**	**21**	47.38	**47.68**^k^	3600	**648**	1413	3609

a **V&S**: Vooradi and Shaik;^17^ relative gap.

b 0.08%.

c 0.84%.

d 2.48%.

e 2.06%.

f 3.06%.

g 0.07%.

h 0.65%.

i 0.64%.

j 0.07%.

k 0.60%.

Table 2 lists the results for makespan minimization with UIS. From Table 2, our models require significantly less computational time to solve examples 1a and 1b to optimality than **V&S**. Specifically, the computational time for example 1a is reduced by 84% (133.7 vs 833.2 s) using **M1** with 14 event points compared with **V&S**. In addition, **M1** proves the optimality in 1 h (i.e., 2578 s) when 15 event points are used, while **V&S** and **M2** cannot. The relative gap from **M2** is 0.47%, which is much smaller than that from **V&S** (4.64%). **M1** outperforms **M2** with a reduction of CPU time by over 28% while solving example 1a with 15 event points, even though it has a relatively larger number of continuous variables and constraints.

Table 3 provides the computational results for maximization of profit with UIS. From Table 3, it is shown that the proposed models **M1**, **M2**, and **V&S** require the same number of event points to generate the optimal solution without allowing a task to span over multiple event points. However, our proposed models require a smaller number of binary variables for examples 2 and 3. This is because our models only examine if there is material transfer between processing units, while **V&S** tests if there is material transfer from a task to another. As multiple tasks share one processing unit in examples 2–3, our models lead to smaller model size and less computational time. Specifically, the computational time is reduced by 1 order of magnitude (721 vs 42 s from **M1**) using our models to find the best result of $1583.442 for Example 3a. Similar reduction in the computational time can also be observed in examples 3b and 3d. Although more constraints are involved in the proposed models, less computational effort is required due to additional tightening constraints imposed. Finally, **M1** shows better performance than **M2** as it can prove optimality for example 3b using 8 event points within 1 h, while **M2** cannot.

**Figure 7:**
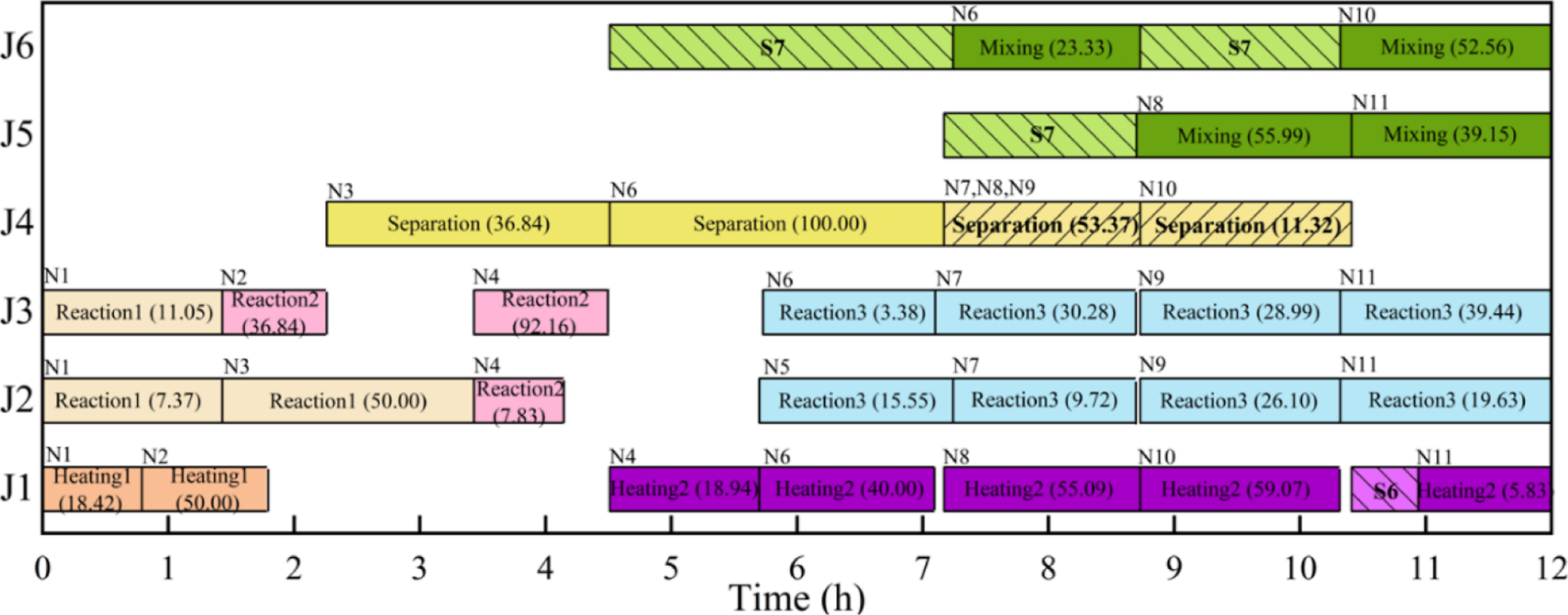
Schedule for the example 12 using **M1–M2** (profit = $1201.39) (
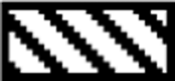
 means one state waits in a unit before processing, 
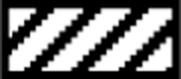
 means batch held in one unit after production).

Table 4 reports the computational results for benchmark examples to maximize profit subject to FIS policy. From Table 4, we can observe that the proposed models lead to a smaller model size and a reduction in the computational time by a factor of 2–3 for Example 3b. More importantly, our proposed model is proven to be more general as a better schedule with higher profit by 21.5% ($1201.39 vs $989.03) is generated for example 12 using our models compared to **V&S**. This is because the batch of materials is permitted to be temporarily held in the suitable processing units over consecutive event points in our models (see the schedule in Figure 7). Specifically, one batch produced by separation at event point N6 is partially held in unit J4 from N7 to N10 to share the storage burden of states S6 and S7.

The results for examples 6, 8, and 9 listed in Table 4 prove that our proposed models do not require a task to span over multiple events (i.e., Δ*n* = 0) due to allowing processing units to hold materials over multiple event points. Therefore, all proposed models lead to significantly smaller model size with less numbers of binary and continuous variables. For instance, our models require 40.2% (79 vs 132) and 56.0% (139 vs 316) fewer binary variables than **V&S** to generate the optimal solution for examples 8 and 9, respectively. As the iterative procedure is often conducted to estimate the appropriate event points, the total test time would be significantly reduced if tasks do not require to span over multiple events using our models.

Table 5 reports the computational statistics for makespan minimization with FIS. From Table 5, we can observe that **M1** proves optimality using dramatically decreased computational time for example 1a and leads to a 16.9% (2.06 vs 2.48%) reduction in the relative gap for example 1b, compared with **V&S**. For example 2b, all the proposed models **M1–M2** can yield the optimal solution of 47.68 h without any task spanning over multiple event points, but **V&S** reaches the optimal solution with Δ*n* = 2. Decrease in the number of binary variables by 40% (648 vs 1080) and computational time by a factor of 43 (82 vs 3600) are realized by **M1** compared with **V&S**. Comparing with **M2**, **M1** always shows better performances due to low relative gaps and short computational times. Specifically, Examples 1a and 2b are not solved to optimality by **M2** within 1 h, while **M1** finds the optimal solution in less than 2000 and 100 s, respectively. In addition, **M1** reports a smaller relative gap for example 1b.

### 5.3 Kallrath Example

We solve the industrial-scale Kallrath example^32,43^ using the extended model **EM1**, **V&S**,^17^ and **VMM**(32) with a resource limit of 40,000 s. The computational results are listed in Table 6. From Table 6, it can be observed that **EM1** leads to 50% less computational time for instance 1 and better objective values for instances 2 and 4–10, compared to **V&S**. **EM1** also requires less computational time (348 vs 447 s) to find the optimal solution for instance 3. **VMM** shows better computational performance, especially in computational efficiency, while solving instances 1–4 as all tasks have fixed and integer processing times in these instances. However, its dominance is diminished with the incremental customer demands. **EM1** finds the best feasible solutions with optimality gaps smaller than 17% within 40,000 s compared with **VMM** and **V&S** for larger instances 6–9. For instance, a better solution with 18% (334 vs 408) lower makespan is generated using **EM1** for instance 7 within the same time resource compared to **VMM**. This is mainly attributed to the huge number of time intervals required in **VMM**, leading to a dramatic rise in the model size and computational effort. For instance 10 with decimal processing times of tasks (see Table S30 in the Supporting Information), **VMM** is hard to find any feasible solution within 40,000 s using the greatest common divisor (i.e., δ = 0.1) of processing times due to the excessive number of discrete variables required. **EM1** leads to 7.6% (91.9 vs 99.6 and 99.5) smaller makespan and over 45% (11.01 vs 20.23 and 25.5%) lower relative gap than **V&S** and **VMM** with δ = 0.5.

**Table 6:** Computational Results for Kallrath Examples with Minimization of Makespan^a^

model	event points/H	RMILP	MILP (h)	CPU time (s)	binary variables	continuous variables	constraints
Instance 1 (*D*_*P*1_ = *D*_*P*3_ = 0, *D*_*P*2_ = *D*_*P*4_ = *D*_*P*5_ = 20 mu)							
**V&S**	10	16	32	10,761	888	1386	5113
**VMM**	100^r^	16	32	167	2448	4546	11,093
**EM1**	10	16	32	5377	591	1267	4229
Instance 2 (*D*_*P*1_ = *D*_*P*2_ = 10 mu, *D*_*P*3_ = *D*_*P*4_ = 20 mu, *D*_*P*5_ = 30 mu)							
**V&S**	11	28	40^b^	40,000	984	1530	5664
**VMM**	100^r^	16	39	5967	2448	4546	11,093
**EM1**	11	28	39	21,890	654	1401	4690
Instance 3 (*D*_*P*1_ = *D*_*P*2_ = *D*_*P*3_ = *D*_*P*4_ = *D*_*P*5_ = 18 mu)							
**V&S**	9	21.6	36	447	792	1242	4562
**VMM**	100^r^	16	36	328	2448	4546	11,093
**EM1**	9	21.6	36	348	528	1133	3768
Instance 4 (*D*_*P*1_ = *D*_*P*2_ = 30 mu, *D*_*P*3_ = 40 mu, *D*_*P*4_ = 20 mu, *D*_*P*5_ = 40 mu)							
**V&S**	15	40	56^c^	40,000	1368	2106	7868
**VMM**	100^r^	32	52	438	2448	4546	11,093
**EM1**	15	40	54^d^	40,000	906	1937	6534
Instance 5 (*D*_*P*1_ = *D*_*P*2_ = 60 mu, *D*_*P*3_ = 80 mu, *D*_*P*4_ = 40 mu, *D*_*P*5_ = 80 mu)							
**V&S**	30	80	108^e^	40,000	2808	4266	16,133
**VMM**	120^r^	72	96	15,757	2928	5446	13,273
**EM1**	30	80	105^f^	40,000	1851	3947	13,449
Instance 6 (*D*_*P*1_ = *D*_*P*3_ = 0, *D*_*P*2_ = *D*_*P*4_ = *D*_*P*5_ = 200 mu)							
**V&S**	88	210.15	268^g^	40,000	8376	12,618	48,091
**VMM**	500^r^	212	NA	40,000	12,048	22,546	54,693
**EM1**	88	210.15	**254**^h^	40,000	5505	11,719	40,187
Instance 7 (*D*_*P*1_ = *D*_*P*2_ = 100 mu, *D*_*P*3_ = *D*_*P*4_ = 200 mu, *D*_*P*5_ = 300 mu)							
**V&**S	90	280	338^i^	40,000	8568	12,906	49,193
**VMM**	500^r^	228	408^j^	40,000	12,048	22,546	54,693
**EM1**	90	280	**334**^k^	40,000	5631	11,987	41,109
Instance 8 (*D*_*P*1_ = *D*_*P*2_ = *D*_*P*3_ = *D*_*P*4_ = *D*_*P*5_ = 180 mu)							
**V&S**	89	218.72	286^l^	40,000	8472	12,762	48,642
**VMM**	500^r^	220	342^m^	40,000	12,048	22,546	54,693
**EM1**	89	218.72	**283**^n^	40,000	5568	11,853	40,648
Instance 9 (*D*_*P*1_ = *D*_*P*2_ = 150 mu, *D*_*P*3_ = 200 mu, *D*_*P*4_ = 100 mu, *D*_*P*5_ = 200 mu)							
**V&S**	78	200	260^o^	40,000	7416	11,178	42,581
**VMM**	500^r^	192	281^p^	40,000	12,048	22,546	54,693
**EM1**	78	200	**254**^q^	40,000	4875	10,379	35,577
Instance 10 (*D*_*P*1_ = *D*_*P*2_ = 60 mu, *D*_*P*3_ = 80 mu, *D*_*P*4_ = 40 mu, *D*_*P*5_ = 80 mu)							
**V&S**	30	70.55	99.6^u^	40,000	2808	4266	16,133
**VMM**	120^s^	72	99.5^v^	40,000	5808	10,846	26,353
	120^t^	72	NA	40,000	28,848	54,046	130,993
**EM1**	30	70.56	**91.9**^w^	40,000	1851	3947	13,449

a **V&S**: Vooradi and Shaik;^17^**VMM**: Velez, Merchan, and Maravelias;^32^ NA: no integer solution found after resource limit exceeded. Relative gap.

b 7.5%.

c 14.29%.

d 3.70%.

e 14.81%.

f 12.31%.

g 16.55%.

h 16.69%.

i 8.88%.

j 32.89%.

k 7.78%%.

l 16.08%.

m 33.04%.

n 15.19%.

o 12.31%.

p 24.36%.

q 10.24%. Step size.

r δ = 1.

s δ = 0.5.

t δ = 0.1. Relative gap.

u 20.23%.

v 25.5%.

w 11.01%.

To further illustrate the robustness and efficiency of the proposed models, we also solve all motivating and benchmark examples using CPLEX 12.8.0/GAMS 25.1.3. The computational results are provided in Section S3 of the Supporting Information. Similar conclusions can be made when using CPLEX 12.8.0.

## 6 Conclusions

In this work, we presented two novel unit-specific event-based MILP models for short-term scheduling of multipurpose batch plants. We introduced the concept of indirect and direct material transfer, allowing efficient sequence or alignment of different tasks in different units. A batch was allowed to be partially transferred to downstream facilities and held in processing time over multiple event points. A new continuous variable denoting the available time of one state was introduced to avoid the limitations in the existing unit-specific event-based models. New tightening constraints were proposed to reduce computational expense.

The computational results for the motivating examples and benchmark examples demonstrated that the proposed models require fewer numbers of discrete variables in most cases, especially where a processing unit can process multiple tasks, compared with the existing unit-specific event-based formulation. Interestingly, all tasks do not need to span over multiple event points to yield optimal solutions for all solved examples. As a result, the computational effort was significantly reduced by a factor ranging from 1 to 43 using the proposed models especially **M1**. In some cases, it was reduced by at least 1 order of magnitude. More importantly, the proposed models can generate the same or better objective values for all solved examples. The increase in profit can range from 21.5 to 67% and the reduction in makespan can vary from 1.05 to 30.4%. This is due to the added flexibility that materials are allowed to be temporarily stored in the suitable processing units. In other words, additional storage space from processing units is provided. Therefore, the proposed models especially model **M1** are superior (e.g., more general) to all existing unit-specific event-based models. Additionally, model **M1** outperforms **M2** on both solution optimality and computational efficiency.

The industrial-scale Kallrath example is more complicated compared to the benchmark examples due to variable recipes and three storage policies involved. Results show that compared with the existing unit-specific event-based formulation, the extended variant **EM1** leads to a reduction in the CPU time over 45% for medium instances and better makespan for large instances. Additionally, for large instances, **EM1** yields competitive solutions with maximum improvement in makespan by 18% compared with the discrete-time formulation,^32^ while the discrete-time formulation shows^32^ better computational performance especially in computational efficiency for medium instances where all tasks have fixed and integer processing times. In the future, we will integrate the proposed model **M1** into the rolling-horizon decomposition framework or develop a more efficient decomposition algorithm to solve larger-scale scheduling problems.

## Supporting Information Available

Unit-specific event-based time representation; STN representations and information of examples; computational results for examples; nomenclature; automatic determination of sets; model **M1**; model **M2**; model **M3**; extended model **M1** (**EM1)**; and differences between *T*_*sn*_^*M*2^ and *T*_*sjn*_ (PDF)

## Abbreviations

STN: state task network
RTN: resource task network
FIS: finite intermediate storage
ZW: zero wait
UIS: unlimited intermediate storage
NIS: no intermediate storage
