# Supplementary material for: Novel Multiple Time-grid Continuous-time Mathematical Formulation for Short-term Scheduling of Multipurpose Batch Plants
Source: Ind Eng Chem Res. 2022 Oct 19;61(43):16093–111. doi: 10.1021/acs.iecr.2c01363 (PMC9634804; doi:10.1021/acs.iecr.2c01363)
Supplement: Supplementary file 1 — ie2c01363_si_001.pdf [file ie2c01363_si_001.pdf]

Supporting Information for
A novel multiple time-grid continuous-time mathematical formulation for short-term scheduling multipurpose batch plants

Dan Li,[1,‡] Nikolaos Rakovitis,[1,‡] Taicheng Zheng,[1] Yueting Pan,[2] Jie Li,[1,*] and Giorgos Kopanos[3]

[1]Centre for Process Integration, Department of Chemical Engineering, The University of Manchester, Manchester, M13 9PL, United Kingdom
[2]Department of Chemical Engineering, The University of Manchester, Manchester, M13 9PL, United Kingdom
[3]Flexciton Limited, London, 145 City Rd, Hoxton, London EC1V 1AZ

S1. Unit-specific event-based time representation

S2. STN representations and information for examples

S3. Computational results for examples

S4. Nomenclature

S5. Automatic determination of sets

S6. Model **M1**

S7. Model **M2**

S8. Model **M3**

S9. Extended Model M1 (**EM1**)

S10. Differences between T_{sn}^{M2} and T_{sjn}

‡ Equal contribution
* To whom correspondence should be addressed. Email: jie.li-2@manchester.ac.uk. Tel: +44 (0) 161 306 8622. Fax: +44 (0) 161 236 7439

Tables

Table S1. Processing times and capacities for Motivating Example 1

Table S2. Processing times and capacities for Motivating Example 2

Table S3. Storage and price data for Motivating Examples 1-2

Table S4. Processing times and capacities for Motivating Example 3

Table S5. Initial amounts and storage capacities for Motivating Example 3

Table S6. Processing information for the Example 1

Table S7. Storage and price data for Example 1

Table S8. Processing times and capacities for Example 2

Table S9. Initial amounts, storage capacities and prices for Example 2

Table S10. Processing times and capacities for Examples 3 and 12

Table S11. Initial amounts, storage capacities and prices for Example 3

Table S12. Initial amounts, storage capacities and prices for Example 12

Table S13. Processing times and capacities for Example 4

Table S14. Initial amounts, storage capacities and prices for Example 4

Table S15. Processing times and capacities for Example 5

Table S16. Storage information and prices for Example 5

Table S17. Processing information for Example 6

Table S18. Storage information and prices for Example 6

Table S19. Processing information for Example 7

Table S20. Storage information and prices for Example 7

Table S21. Processing information for Example 8

Table S22. Processing information for Example 9

Table S23. Storage information and prices for Example 8

Table S24. Storage information and prices for Example 9

Table S25. Processing information for Example 10

Table S26. Storage information and prices for Example 10

Table S27. Processing information for Example 11

Table S28. Storage information and prices for Example 11

Table S29. Processing information for Instance 1-9 of the Kallrath Example

Table S30. Processing information for Instance 10 of the Kallrath Example

Table S31. Storage information and prices for the Kallrath Example

Table S32. Value of M for Big-M constraints with minimization of makespan

Table S33. Computational results for Motivating Examples

Table S34. Computational results for Examples 1-3 maximizing profit with UIS

Table S35. Computational results for Examples 4-9 maximizing profit with UIS

Table S36. Computational results for Examples 1-3 maximizing profit with FIS

Table S37. Computational results for Examples 4-12 maximizing profit with FIS

Table S38. Computational results for Examples 1-3 minimizing Makespan with UIS

Table S39. Computational results for examples 1-3 minimizing Makespan with FIS

Table S40. Computational results for Motivating Examples

Table S41. Computational results for Examples 1-3 maximizing profit with UIS

Table S42. Computational results for Examples 4-9 maximizing profit with UIS

Table S43. Computational results for Examples 1-3 maximizing profit with FIS

Table S44. Computational results for Examples 4-12 maximizing profit with FIS

Table S45. Computational results for Examples 1-3 minimizing makespan with UIS

Table S46. Computational results for Examples 1-3 minimizing makespan with FIS

Figures

Figure S1. Schematic diagram of the unit-specific event-based time representation

Figure S2. STN representation for the Motivating Examples 1-2

Figure S3. STN representation for Motivating Example 3

Figure S4. STN representation for Example 1

Figure S5. STN representation for Example 2

Figure S6. STN representation for Examples 3 and 12

Figure S7. STN representation for Example 4

Figure S8. STN representation for Example 5

Figure S9. STN representation for Example 6

Figure S10. STN representation for Example 7

Figure S11. STN representation for Examples 8-9

Figure S12. STN representation of the Example 10

Figure S13. STN representation for Example 11

Figure S14. STN representation for the Kallrath Example

Figure S15. Optimal schedule for the Example 12 using V&S model

Figure S16. An example solution to illustrate the differences between T_{sn}^{M2} in model **M2** and T_{sjn} in model **M3**

S1. Unit-specific event-based time representation

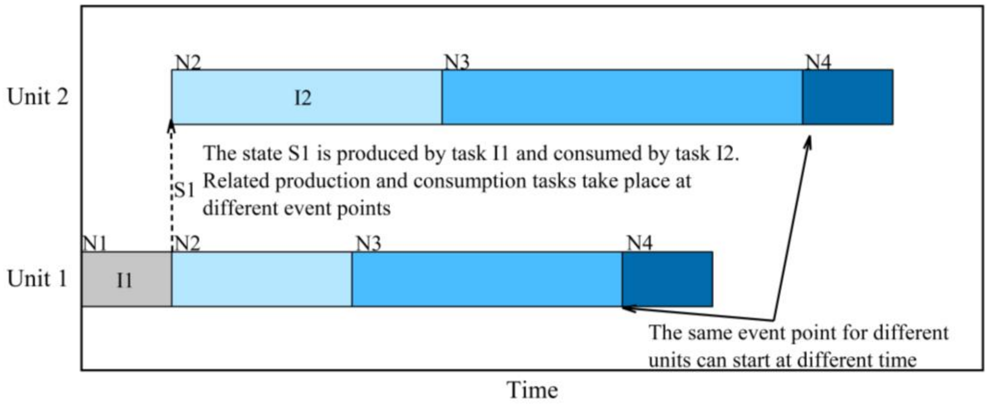

Figure S1. Schematic diagram of the unit-specific event-based time representation

S2. STN representations and information for examples

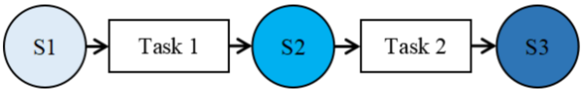

Figure S2. STN representation for the Motivating Examples 1-2

Table S1. Processing times and capacities for Motivating Example 1

Task	Processing Unit	α_i (h)	β_i(h mu^{-1})	B_i^{min}(mu)	B_i^{max}(mu)
1	1	3	0.02	0	100
2	2	1	0.01	0	50

Table S2. Processing times and capacities for Motivating Example 2

Task	Processing Unit	α_i (h)	β_i(h mu^{-1})	B_i^{min}(mu)	B_i^{max}(mu)
1	1	5	0	0	100
2	2	1.5	0	0	50

Table S3. Storage and price data for Motivating Examples 1-2

	S1	S2	S3
$ST0_s$ (mu)	$+\infty$	0	0
ST_s^{max} (mu)	$+\infty$	10	$+\infty$
p_s (\$ mu^{-1})	0	0	5

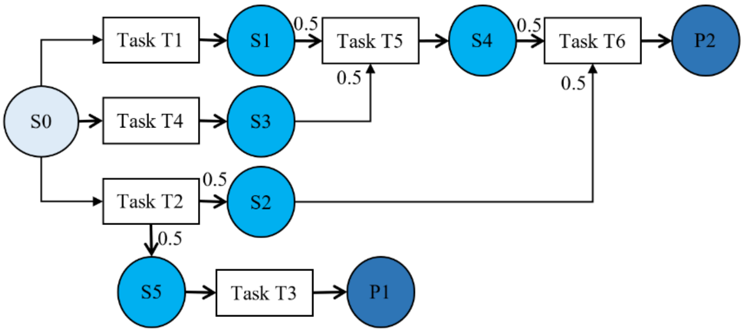

Figure S3. STN representation for Motivating Example 3

Table S4. Processing times and capacities for Motivating Example 3

Task	Processing Unit	α_i(h)	β_i(h mu^{-1})	B_i^{min}(mu)	B_i^{max}(mu)
T1	J1	1.00	0	0	50
T2	J1	1.00	0	0	25
T3	J1	10.00	0	0	100
T4	J2	10.00	0	0	50
T5	J2	1.00	0	0	12.5
T6	J2	1.00	0	0	200

Table S5. Initial amounts and storage capacities for Motivating Example 3

	S0	S1	S2	S3	S4	S5	P1	P2
$ST0_s$ (mu)	$+\infty$	0	0	0	0	0	0	0
ST_s^{max} (mu)	$+\infty$	1000	1000	1000	1000	1000	$+\infty$	$+\infty$

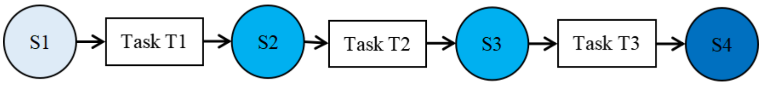

Figure S4. STN representation for Example 1

Table S6. Processing information for the Example 1

Task	Processing Unit	α_i (h)	β_i(h mu^{-1})	B_i^{min}(mu)	B_i^{max}(mu)
T1	Unit 1	1.33	0.01333	0	100
	Unit 2	1.33	0.01333	0	150
T2	Unit 3	1.00	0.00500	0	200
T3	Unit 4	0.667	0.00445	0	150
	Unit 5	0.667	0.00445	0	150

Table S7. Storage and price data for Example 1

	S1	S2	S3	S4
$ST0_s$ (mu)	+∞	0	0	0
ST_s^{max} (mu)	+∞	200	250	+∞
p_s (\$ mu^{-1})	0	0	0	5

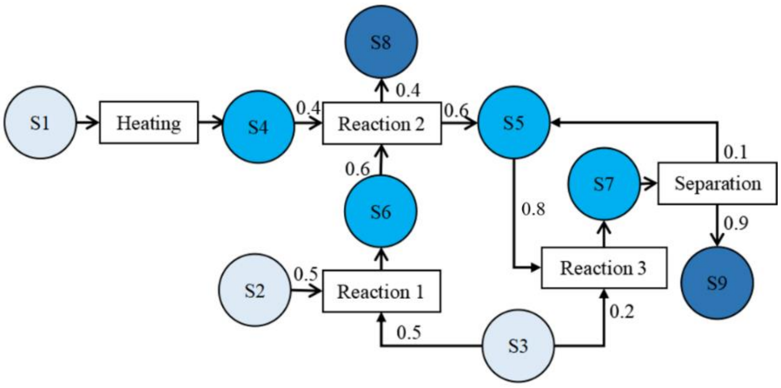

Figure S5. STN representation for Example 2

Table S8. Processing times and capacities for Example 2

Task	Processing Unit	α_i (h)	β_i(h mu^{-1})	B_i^{min}(mu)	B_i^{max}(mu)
Heating	Heater	0.6670	0.006700	0	100
Reaction1	Reactor 1	1.3340	0.026640	0	50
	Reactor 2	1.3340	0.016650	0	80
Reaction2	Reactor 1	1.3340	0.026640	0	50
	Reactor 2	1.3340	0.016650	0	80
Reaction3	Reactor 1	0.6670	0.013320	0	50
	Reactor 2	0.6670	0.008325	0	80
Separation	Filter	1.3342	0.006660	0	200

Table S9. Initial amounts, storage capacities and prices for Example 2

	S1	S2	S3	S4	S5	S6	S7	S8	S9
$ST0_s$ (mu)	+∞	+∞	+∞	0	0	0	0	0	0
ST_s^{max} (mu)	+∞	+∞	+∞	100	200	150	200	+∞	+∞
p_s (\$mu^{-1})	0	0	0	0	0	0	0	10	10

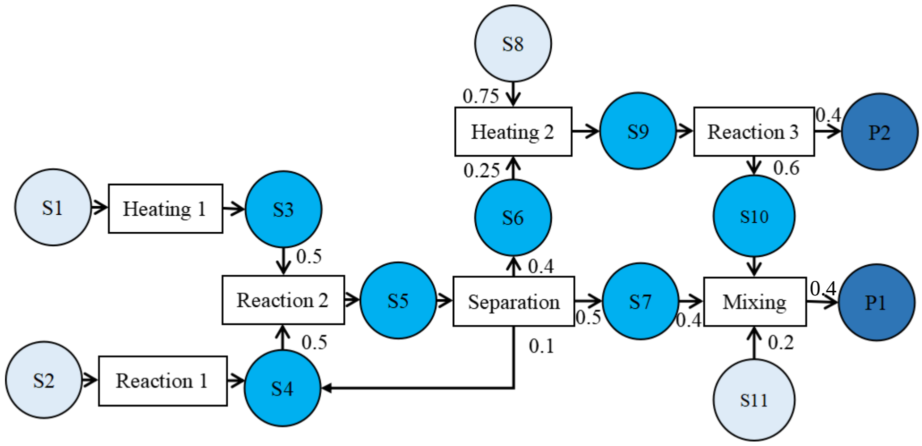

Figure S6. STN representation for Examples 3 and 12

Table S10. Processing times and capacities for Examples 3 and 12

Task	Processing Unit	α_i (h)	β_i(h mu^{-1})	B_i^{min}(mu)	B_i^{max}(mu)
Heating 1	Heater	0.667	0.00667	0	100
Heating 2	Heater	1.000	0.01000	0	100
Reaction 1	Reactor 1	1.333	0.01333	0	100
	Reactor 2	1.333	0.00889	0	150
Reaction2	Reactor 1	0.667	0.00667	0	100
	Reactor 2	0.667	0.00445	0	150
Reaction3	Reactor 1	1.333	0.01330	0	100
	Reactor 2	1.333	0.00889	0	150
Separation	Separator	2.000	0.00667	0	300
Mixing	Mixer 1	1.333	0.00667	20	200
	Mixer 2	1.333	0.00667	20	200

Table S11. Initial amounts, storage capacities and prices for Example 3

	S1	S2	S3	S4	S5	S6	S7	S8	S9	S10	S11	P1	P2
$ST0_s$ (mu)	+∞	+∞	0	0	0	50	50	+∞	0	0	+∞	0	0
ST_s^{max} (mu)	+∞	+∞	100	100	300	150	150	+∞	150	150	+∞	+∞	+∞
p_s ($/mu)	0	0	0	0	0	0	0	0	0	0	0	5	5

Table S12. Initial amounts, storage capacities and prices for Example 12

	S1	S2	S3	S4	S5	S6	S7	S8	S9	S10	S11	P1	P2
$ST0_s$ (mu)	+∞	+∞	0	0	0	0	0	+∞	0	0	+∞	0	0
ST_s^{max} (mu)	+∞	+∞	100	100	10	10	10	+∞	150	150	+∞	+∞	+∞
p_s ($/mu)	0	0	0	0	0	0	0	0	0	0	0	5	5

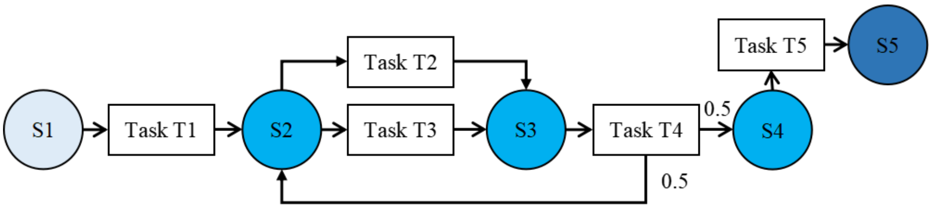

Figure S7. STN representation for Example 4

Table S13. Processing times and capacities for Example 4

Task	Processing Unit	α_i (h)	β_i(h mu^{-1})	B_i^{min}(mu)	B_i^{max}(mu)
T1	Unit 1	0.9500	0.010000	0	6
T2	Unit 2	2.9400	0.020000	0	3
T3	Unit 3	2.4800	0.010000	0	2
T4	Unit 4	4.4666	0.006680	0	6
T5	Unit 5	1.9663	0.013348	0	8

Table S14. Initial amounts, storage capacities and prices for Example 4

	S1	S2	S3	S4	S5
$ST0_s$ (mu)	+∞	0	0	0	0
ST_s^{max} (mu)	+∞	3	4	8	+∞
p_s ($ mu^{-1})	0	0	0	0	1

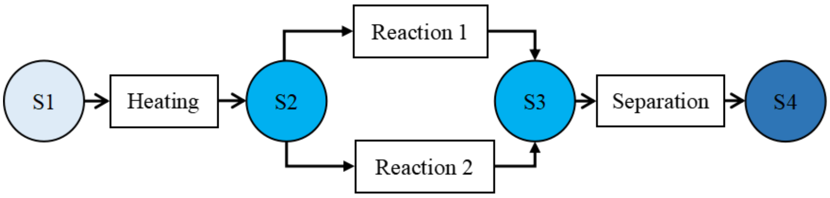

Figure S8. STN representation for Example 5

Table S15. Processing times and capacities for Example 5

Task	Processing Unit	α_i (h)	β_i(h mu^{-1})	B_i^{min}(mu)	B_i^{max}(mu)
Heating	Unit 1	1	0	0	10
Reaction 1	Unit 2	3	0	0	4
Reaction 2	Unit 3	1	0	0	2
Separation	Unit 4	2	0	0	10

Table S16. Storage information and prices for Example 5

	S1	S2	S3	S4
$ST0_s$ (mu)	+∞	0	0	0
ST_s^{max} (mu)	+∞	6	4	+∞
p_s ($/mu)	0	0	0	1

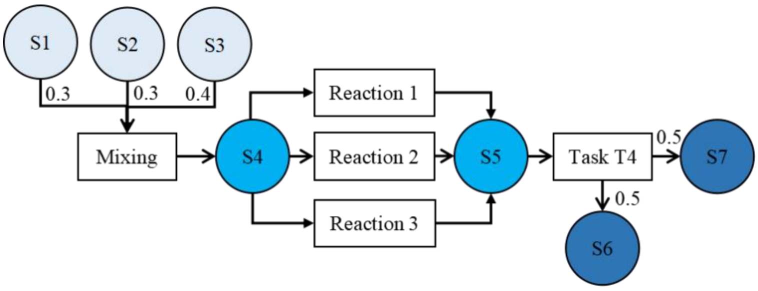

Figure S9. STN representation for Example 6

Table S17. Processing information for Example 6

Task	Processing Unit	α_i (h)	β_i(h mu^{-1})	B_i^{min}(mu)	B_i^{max}(mu)
Mixing	Unit 1	1.5	0	0	150
Reaction1	Unit 2	4.5	0	0	60
Reaction2	Unit 3	1.5	0	0	30
Reaction3	Unit 4	1.5	0	0	30
T4	Unit 5	3.0	0	0	150

Table S18. Storage information and prices for Example 6

	S1	S2	S3	S4	S5	S6	S7
$ST0_s$ (mu)	+∞	+∞	+∞	0	0	0	0
ST_s^{max} (mu)	+∞	+∞	+∞	60	60	+∞	+∞
p_s ($/mu)	0	0	0	0	0	1	1

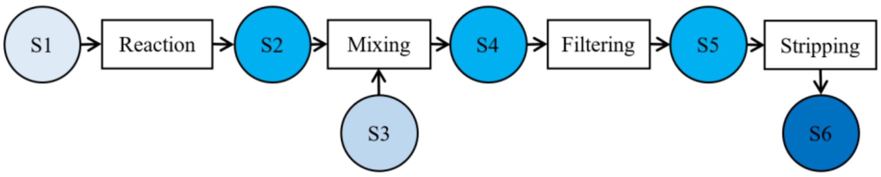

Figure S10. STN representation for Example 7

Table S19. Processing information for Example 7

Task	Processing Unit	α_i (h)	β_i(h)	B_i^{min}(mu)	B_i^{max}(mu)
Reaction	Unit 1	17.333	0.866	0	20
Mixing	Unit 2	2.667	0.133	0	20
	Unit 3	2.667	0.133	0	20
Filtering	Unit 4	4.000	0.200	0	20
	Unit 5	5.333	0.266	0	20
Stripping	Unit 6	5.333	0.266	0	20

Table S20. Storage information and prices for Example 7

	S1	S2	S3	S4	S5	S6
$ST0_s$ (mu)	+∞	0	+∞	0	0	0
ST_s^{max} (mu)	+∞	100	+∞	100	100	+∞
p_s ($/mu)	0	0	0	0	0	2

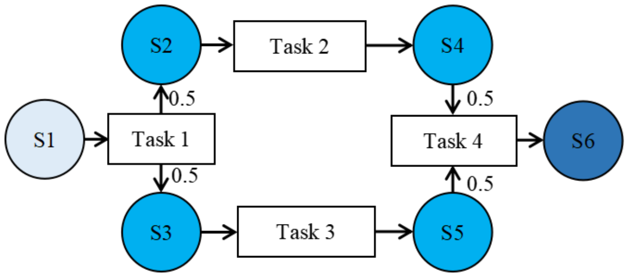

Figure S11. STN representation for Examples 8-9

Table S21. Processing information for Example 8

Task	Processing Unit	α_i (h)	β_i(h mu^{-1})	B_i^{min}(mu)	B_i^{max}(mu)
T1	Unit 1	1.666	0.03335	0	40
T2	Unit 2	2.333	0.08335	0	20
T3	Unit 3	0.667	0.06660	0	5
T4	Unit 4	2.667	0.008325	0	40

Table S22. Processing information for Example 9

Task	Processing Unit	α_i (h)	β_i(h mu^{-1})	B_i^{min}(mu)	B_i^{max}(mu)
T1	Unit 1	1.666	0.03335	0	40
T2	Unit 2	2.333	0.08335	0	20
T3	Unit 3	0.333	0.06680	0	2.5
T4	Unit 4	2.667	0.008325	0	40

Table S23. Storage information and prices for Example 8

	S1	S2	S3	S4	S5	S6
$ST0_s$ (mu)	+∞	0	0	0	0	0
ST_s^{max} (mu)	+∞	10	15	10	15	+∞
p_s ($ mu^{-1})	0	0	0	0	0	10

Table S24. Storage information and prices for Example 9

	S1	S2	S3	S4	S5	S6
$ST0_s$ (mu)	+∞	0	0	0	0	0
ST_s^{max} (mu)	+∞	10	17.5	10	18	+∞
p_s ($ mu^{-1})	0	0	0	0	0	10

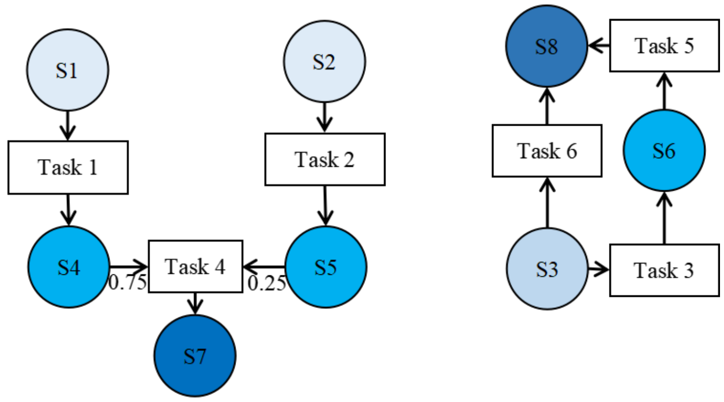

Figure S12. STN representation of the Example 10

Table S25. Processing information for Example 10

Task	Processing Unit	α_i (h)	β_i(h mu^{-1})	B_i^{min}(mu)	B_i^{max}(mu)
T1	Unit 1	1.666	0.0778	0	30
T2	Unit 2	2.333	0.0667	0	10
T3	Unit 3	0.669	0.0777	0	30
T4	Unit 3	0.667	0.03325	0	40
T5	Unit 2	1.332	0.0556	0	30
T6	Unit 1	1.500	0.025	0	20

Table S26. Storage information and prices for Example 10

	S1	S2	S3	S4	S5	S6	S7	S8
$ST0_s$ (mu)	+∞	+∞	+∞	0	0	0	0	0
ST_s^{max} (mu)	+∞	+∞	+∞	10	5	10	+∞	+∞
p_s (\$ mu^{-1})	0	0	0	0	0	0	10	5

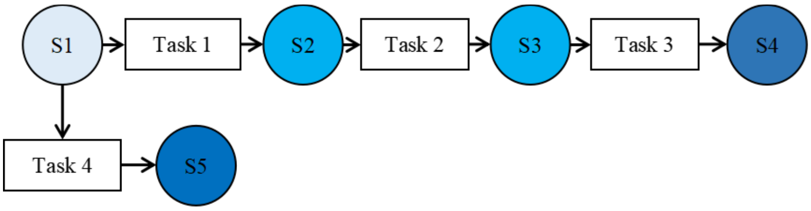

Figure S13. STN representation for Example 11

Table S27. Processing information for Example 11

Task	Processing Unit	α_i (h)	β_i(h mu^{-1})	B_i^{min}(mu)	B_i^{max}(mu)
T1	Unit 1	2	0	0	260
T2	Unit 2	3	0	0	140
	Unit 3	2	0	0	120
T3	Unit 4	3	0	0	120
	Unit 5	2	0	0	140
T4	Unit 1	1	0	0	1
	Unit 2	3	0	0	1

Table S28. Storage information and prices for Example 11

	S1	S2	S3	S4	S5
$ST0_s$ (mu)	+∞	0	0	0	0
ST_s^{max} (mu)	+∞	10	10	+∞	+∞
p_s (\$ mu^{-1})	0	0	0	1	1

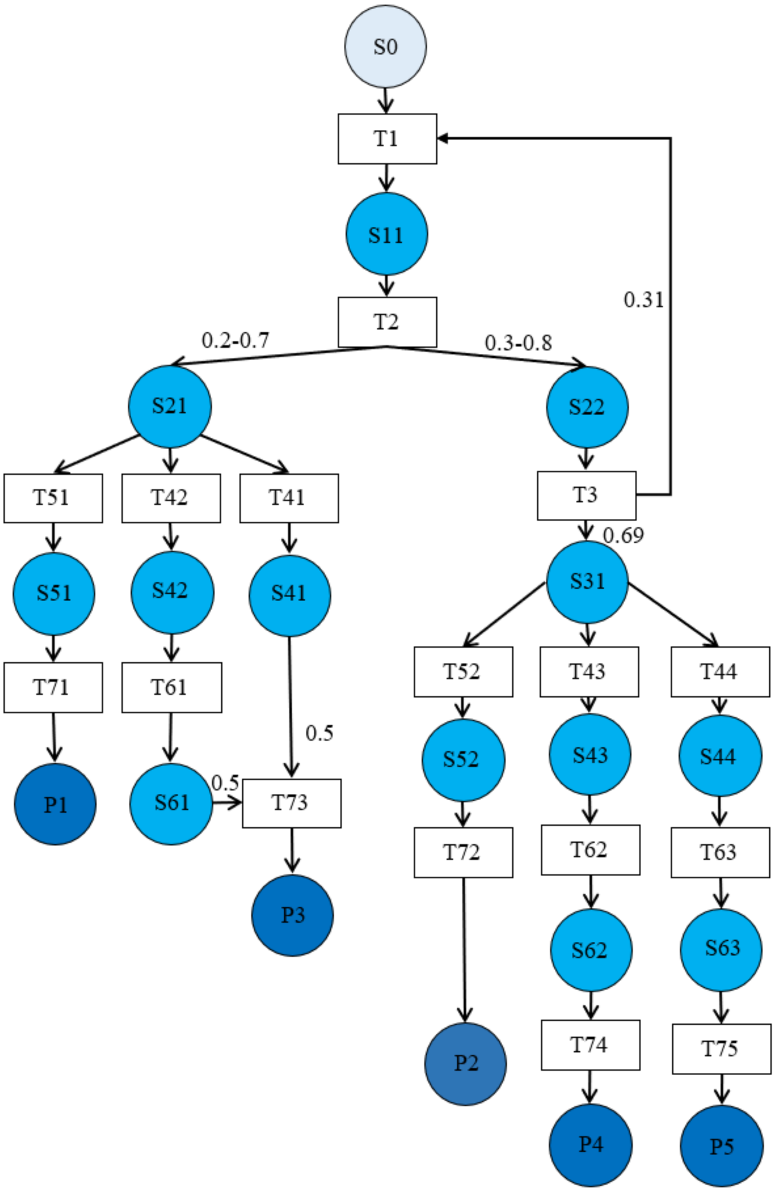

Figure S14. STN representation for the Kallrath Example

Table S29. Processing information for Instance 1-9 of the Kallrath Example

Task	Processing Unit	α_i (h)	β_i(h)	B_i^{min}(kg)	B_i^{max}(kg)
T1	Unit 1	2	0	3	10
T2	Unit 2	4	0	5	20
T3	Unit 3	2	0	4	10
T41	Unit 4	4	0	4	10
T42	Unit 4	4	0	4	10
T43	Unit 4	4	0	4	10
T44	Unit 4	4	0	4	10
T51	Unit 5	6	0	4	10
T52	Unit 5	6	0	4	10
T61	Unit 6	4	0	3	7
	Unit 7	5	0	3	7
T62	Unit 6	5	0	3	7
	Unit 7	6	0	3	7
T63	Unit 6	6	0	3	7
	Unit 7	6	0	3	7
T71	Unit 8	4	0	4	12
	Unit 9	6	0	4	12
T72	Unit 8	4	0	4	12
	Unit 9	6	0	4	12
T73	Unit 8	4	0	4	12
T74	Unit 8	6	0	4	12
	Unit 9	6	0	4	12
T75	Unit 8	6	0	4	12
	Unit 9	6	0	4	12

Table S30. Processing information for Instance 10 of the Kallrath Example

Task	Processing Unit	α_i (h)	β_i(h)	B_i^{min}(kg)	B_i^{max}(kg)
T1	Unit 1	2	0	3	10
T2	Unit 2	4	0	5	20
T3	Unit 3	2	0	4	10
T41	Unit 4	3.2	0	4	10
T42	Unit 4	3.2	0	4	10
T43	Unit 4	3.2	0	4	10
T44	Unit 4	3.2	0	4	10
T51	Unit 5	5.4	0	4	10
T52	Unit 5	5.4	0	4	10
T61	Unit 6	4	0	3	7
	Unit 7	5	0	3	7
T62	Unit 6	5	0	3	7
	Unit 7	6	0	3	7
T63	Unit 6	6	0	3	7
	Unit 7	6	0	3	7
T71	Unit 8	4	0	4	12
	Unit 9	6	0	4	12
T72	Unit 8	4	0	4	12
	Unit 9	6	0	4	12
T73	Unit 8	3.5	0	4	12
T74	Unit 8	5.5	0	4	12
	Unit 9	5.5	0	4	12
T75	Unit 8	5.5	0	4	12
	Unit 9	5.5	0	4	12

Table S31. Storage information and prices for the Kallrath Example

	S0	S11	S21	S22	S31	S41	S42	S43	S44	S51	S52
$ST0_s$ (kg)	+∞	20	20	0	20	0	0	0	0	0	0
ST_s^{max} (kg)	+∞	30	30	15	30	ZW	10	10	10	ZW	ZW

	S61	S62	S63	P1	P2	P3	P4	P5
$ST0_s$ (kg)	0	0	0	0	0	0	0	0
ST_s^{max} (kg)	10	ZW	10	+∞	+∞	+∞	+∞	+∞

Table S32. Value of M for Big-M constraints with minimization of makespan

Problem		M
Motivating Example 2		100
Motivating Example 3		100
Example 1a		50
Example 1b		100
Example 2a		50
Example 2b		100
Example 3a		50
Example 3b		100
Kallrath Example	Instance 1	100
	Instance 2	100
	Instance 3	100
	Instance 4	100
	Instance 5	120
	Instance 6	500
	Instance 7	500
	Instance 8	500
	Instance 9	500

S3. Computational results for examples

Note that computational results in Tables S33-39 are generated using **CPLEX 12.8.0/GAMS 25.1.3**, whilst those in Tables S40-46 using **CPLEX 12.6.3/GAMS 24.6.1**, on a desktop computer with AMD Ryzen™ 9 3900X 3.8 GHz and 48 GB RAM running Windows 10.

Table S33. Computational results for Motivating Examples

Model	Event Points/H	RMILP	MILP	CPU time (s)	Binary variables	Continuous variables	Constraints
			Motivating Example 1 (H = 8h)				
**V&S**[17]	3	500.00	300.00	0.05	12	32	66
**L&F**[14]	3	500.00	300.00	0.11	6	29	41
**SLK2**[10]	4	500.00	300.00	0.14	12	81	94
**M1**	3	500.00	500.00	0.03	12	37	72
**M2**	3	500.00	500.00	0.03	12	40	72
**M3**	3	500.00	500.00	0.03	12	37	75
			Motivating Example 2 (D_{S3} = 100 mu)				
**V&S**[17]	3	5.00	11.5	0.02	12	32	68
**L&F**[14]	3	5.00	11.5	0.11	6	29	43
**SLK2**[10]	4	5.00	11.5	0.02	12	83	99
**VMM**[32]	100[a]	5.00	11.5	0.22	404	1006	2822
**M1**	3	8.00	**8**	0.03	12	37	76
**M2**	3	8.00	**8**	0.03	12	41	81
**M3**	3	8.00	**8**	0.03	12	37	79
			Motivating Example 3 (D_{P1} =100 mu, D_{P2} = 200 mu)				
**V&S**[17]	10	27.00	27.00	0.05	114	350	898
	11	19.00	26.00	0.19	126	386	994
	12	19.00	25.00	0.38	138	422	1090
	18	19.00	25.00	52.1	210	638	1666
	18($\Delta n = 1$)	19.00	24.00	2116	312	740	2074
	18($\Delta n = 2$)	19.00	23.00	3466	408	836	2362
	18($\Delta n = 3$)	19.00	22.00	2031	498	926	2632
	18($\Delta n = 4$)	19.00	21.00	1312	582	1010	2884
	18($\Delta n = 5$)	19.00	20.00	528	660	1088	3118
	18($\Delta n = 6$)	19.00	19.00	25	732	1160	3334
**L&F**[14]	10	28.00	28.00	0.19	60	262	448
	11	19.00	26.00	0.16	66	288	494
	12	19.00	25.00	0.27	72	314	540
	13	19.00	24.00	0.84	78	340	586
	14	19.00	23.00	1.92	84	366	632
	15	19.00	22.00	2.58	90	392	678
	16	19.00	21.00	3.61	96	418	724
	17	19.00	20.00	1.53	102	444	770
	18	19.00	19.00	0.25	108	470	816
**SLK2**[10]	11	28.00	28.00	0.05	80	534	733
	12	19.00	19.00	0.19	88	584	805
**VMM**[32]	100[b]	10.00	19.00	0.86	612	1415	3256
**M1**	**10**	19.00	**19.00**	0.05	114	479	803
**M2**	**10**	19.00	**19.00**	0.14	114	403	856
**M3**	**10**	19.00	**19.00**	0.05	114	479	773

[a]: step size: $\delta = 0.5$, [b]: step size: $\delta = 0.5$.

Table S34. Computational results for Examples 1-3 maximizing profit with UIS

Model	Event points	RMILP	MILP	CPU time (s)	Binary variables	Continuous variables	Constraints
			Example 1a (H = 8h)				
**V&S**	4	2000.00	1840.17	0.13	32	89	172
	5	3000.00	1840.17	0.19	41	112	221
**M1**	4	2000.00	1840.17	0.05	32	99	208
	5	3000.00	1840.17	0.17	41	125	273
**M2**	4	2000.00	1840.17	0.03	32	98	200
	5	3000.00	1840.17	0.16	41	123	260
**M3**	4	2000.00	1840.17	0.05	32	99	228
	5	3000.00	1840.17	0.17	41	125	298
			Example 1b (H = 10h)				
**V&S**	5	3000.00	2628.19	0.08	41	112	221
	6	4000.00	2628.19	0.63	50	135	270
**M1**	5	3000.00	2628.19	0.05	41	125	273
	6	4000.00	2628.19	0.28	50	151	338
**M2**	5	3000.00	2628.19	0.05	41	123	260
	6	4000.00	2628.19	0.30	50	148	320
**M3**	5	3000.00	2628.19	0.03	41	125	298
	6	4000.00	2628.19	0.28	50	151	368
			Example 1c (H = 12h)				
**V&S**	6	4000.00	3463.62	0.19	50	135	270
	7	4951.24	3463.62	1.16	59	158	319
**M1**	6	4000.00	3463.62	0.14	50	151	338
	7	4951.24	3463.62	0.63	59	177	403
**M2**	6	4000.00	3463.62	0.16	50	148	320
	7	4951.24	3463.62	0.72	59	173	380
**M3**	6	4000.00	3463.62	0.05	50	151	368
	7	4951.24	3463.62	1.08	59	177	438
			Example 1d (H = 16h)				
**V&S**	9	6601.65	5038.05	20.19	77	204	417
	10	6601.65	5038.05	464.06	86	227	466
**M1**	9	6601.65	5038.05	6.63	77	229	533
	10	6601.65	5038.05	75.53	86	255	598
**M2**	9	6601.65	5038.05	6.67	77	223	500
	10	6601.65	5038.05	79.48	86	248	560
**M3**	9	6601.65	5038.05	8.80	77	229	578
	10	6601.65	5038.05	145.05	86	255	648
			Example 2a (H = 8h)				
**V&S**	4	1730.87	1498.57	0.05	62	176	348
	5	2123.32	1498.57	0.28	80	223	454
**M1**	4	1730.87	1498.57	0.05	56	248	380
	5	2123.32	1498.57	0.28	72	321	504
**M2**	4	1730.87	1498.57	0.05	56	162	354
	5	2123.32	1498.57	0.38	72	205	463

**M3**	4	1730.87	1498.57	0.05	56	248	396
	5	2123.32	1498.57	0.30	72	321	524
			Example 2b (H = 10h)				
**V&S**	5	2436.69	1962.70	0.19	80	223	454
	6	2730.66	1962.70	3.75	98	270	560
**M1**	5	2436.69	1962.70	0.17	72	321	504
	6	2730.66	1962.70	3.77	88	394	628
**M2**	5	2436.69	1962.70	0.17	72	205	463
	6	2730.66	1962.70	3.39	88	248	572
**M3**	5	2436.69	1962.70	0.17	72	321	524
	6	2730.66	1962.70	3.23	88	394	652
			Example 2c (H = 12h)				
**V&S**	6	3076.62	2658.52	0.41	98	270	560
	7	3301.03	2658.52	6.94	116	317	666
**M1**	6	3076.62	2658.52	0.30	88	394	628
	7	3301.03	2658.52	6.28	104	467	752
**M2**	6	3076.62	2658.52	0.30	88	248	572
	7	3301.03	2658.52	6.99	104	291	681
**M3**	6	3076.62	2658.52	0.31	88	394	652
	7	3301.03	2658.52	6.94	104	467	780
			Example 2d (H = 16h)				
**V&S**	8	4291.68	3738.38	12.98	134	364	772
	9	4438.96	3738.38	478.34	152	411	878
**M1**	8	4291.68	3738.38	12.20	120	540	876
	9	4438.96	3738.38	486.19	136	613	1000
**M2**	8	4291.68	3738.38	13.64	120	334	790
	9	4438.96	3738.38	602.97	136	377	899
**M3**	8	4291.68	3738.38	10.73	120	540	908
	9	4438.96	3738.38	405.00	136	613	1036
			Example 3a (H = 8h)				
**V&S**	5	2100.00	1583.44	1.24	123	309	682
	6	2750.96	1583.44	1211.61	151	374	842
**M1**	5	2100.00	1583.44	1.17	115	469	815
	6	2750.96	1583.44	57.03	141	576	1016
**M2**	5	2100.00	1583.44	0.70	115	296	762
	6	2750.96	1583.44	47.63	141	358	943
**M3**	5	2100.00	1583.44	0.95	115	469	845
	6	2750.96	1583.44	73.05	141	576	1052
			Example 3b (H = 10h)				
**V&S**	7	3369.69	2358.20	436.45	179	439	1002
	8	3618.64	2358.20[a]	3600	207	504	1162
**M1**	7	3369.69	2358.20	53.95	167	683	1217
	8	3618.64	2358.20	2313.55	193	790	1418
**M2**	7	3369.69	2358.20	81.47	167	420	1124
	8	3618.64	2358.20	1625.39	193	482	1305
**M3**	7	3369.69	2358.20	109.20	167	683	1259

	8	3618.64	2358.20[b]	3600	193	790	1466
			Example 3c (H = 12h)				
**V&S**	7	3465.63	3041.27	0.50	179	439	1002
	8	3988.41	3041.27	5.27	207	504	1162
**M1**	7	3465.63	3041.27	1.06	167	683	1217
	8	3988.41	3041.27	5.09	193	790	1418
**M2**	7	3465.63	3041.27	0.94	167	420	1124
	8	3988.41	3041.27	9.02	193	482	1305
**M3**	7	3465.63	3041.27	1.28	167	683	1259
	8	3988.41	3041.27	4.47	193	790	1466
			Example 3d (H = 16h)				
**V&S**	10	5225.86	4262.80	91.67	263	634	1482
	11	5644.59	4262.80[c]	3600	291	699	1642
**M1**	10	5225.86	4262.80	96.16	245	1004	1820
	11	5644.59	4262.80	3113.50	271	1111	2021
**M2**	10	5225.86	4262.80	59.92	245	606	1667
	11	5644.59	4262.80	3144.05	271	668	1848
**M3**	10	5225.86	4262.80	45.11	245	1004	1880
	11	5644.59	4262.80[d]	3600	271	1111	2087

Relative gap, [a]: 0.30%, [b]: 0.15%, [c]: 0.85, [d]: 0.01%.

Table S35. Computational results for Examples 4-9 maximizing profit with UIS

Model	Event points	RMILP	MILP	CPU time (s)	Binary variables	Continuous variables	Constraints
			Example 4 (H = 15h)				
**V&S**	6	7.50	5.32	0.06	65	156	338
	7	9.92	5.32	0.30	77	183	401
**M1**	6	7.50	5.32	0.06	65	182	479
	7	9.74	5.32	0.19	77	214	573
**M2**	6	7.50	5.32	0.06	65	175	442
	7	9.74	5.32	0.16	77	205	526
**M3**	6	7.50	5.32	0.05	65	182	509
	7	9.74	5.32	0.19	77	214	608
			Example 5 (H = 6h)				
**V&S**	5	14.00	10.00	0.05	36	97	196
	6	16.00	10.00	0.08	44	117	240
**M1**	5	14.00	10.00	0.05	36	110	254
	6	15.79	10.00	0.05	44	133	315
**M2**	5	14.00	10.00	0.05	36	108	241
	6	15.79	10.00	0.05	44	130	297
**M3**	5	14.00	10.00	0.03	36	110	274
	6	15.79	10.00	0.08	44	133	339
			Example 6 (H = 9h)				
**V&S**	5	300.00	210.00	0.06	49	136	265
	6	360.00	210.00	0.19	60	164	325

**M1**	5	300.00	210.00	0.05	49	154	353
	6	353.68	210.00	0.16	60	186	438
**M2**	5	300.00	210.00	0.03	49	148	331
	6	353.68	210.00	0.14	60	178	408
**M3**	5	300.00	210.00	0.06	49	154	378
	6	353.68	210.00	0.16	60	186	468
			Example 7 (H = 76h)				
**V&S**	5	80.00	58.99	0.05	54	146	294
	6	87.73	58.99	0.38	66	176	360
**M1**	5	80.00	58.99	0.05	54	163	375
	6	87.73	58.99	0.16	66	197	465
**M2**	5	80.00	58.99	0.05	54	162	360
	6	87.73	58.99	0.16	66	195	444
**M3**	5	80.00	58.99	0.05	54	163	405
	6	87.73	58.99	0.16	66	197	501
			Example 8 (H = 10h)				
**V&S**	6	400.00	400.00	0.08	44	129	272
	7	500.00	400.00	0.17	52	151	322
**M1**	6	400.00	400.00	0.05	44	150	361
	7	500.00	400.00	0.14	52	176	431
**M2**	6	400.00	400.00	0.03	44	154	349
	7	500.00	400.00	0.28	52	180	415
**M3**	6	400.00	400.00	0.06	44	150	385
	7	500.00	400.00	0.19	52	176	459
			Example 9 (H = 10h)				
**V&S**	10	400.00	400.00	0.06	76	217	472
	11	450.00	400.00	0.41	84	239	522
**M1**	10	400.00	400.00	0.03	76	254	641
	11	450.00	400.00	0.30	84	280	711
**M2**	10	400.00	400.00	0.14	76	258	613
	11	450.00	400.00	0.36	84	284	679
**M3**	10	400.00	400.00	0.05	76	254	681
	11	450.00	400.00	0.50	84	280	755

Table S36. Computational results for Examples 1-3 maximizing profit with FIS

Model	Event points	RMILP	MILP	CPU time (s)	Binary variables	Continuous variables	Constraints
			Example 1a (H = 8h)				
**V&S**	4	2000.00	1840.17	0.08	56	101	256
	5	3000.00	1840.17	0.19	73	128	333
**M1**	4	2000.00	1840.17	0.05	53	120	293
	5	3000.00	1840.17	0.28	69	153	387
**M2**	4	2000.00	1840.17	0.03	53	125	288
	5	3000.00	1840.17	0.30	69	159	379
**M3**	4	2000.00	1840.17	0.03	53	120	305

	5	3000.00	1840.17	0.19	69	153	402
Example 1b (H = 10h)							
**V&S**	5	3000.00	2628.19	0.08	73	128	333
	6	4000.00	2628.19	0.52	90	155	410
**M1**	5	3000.00	2628.19	0.05	69	153	387
	6	4000.00	2628.19	0.61	85	186	481
**M2**	5	3000.00	2628.19	0.14	69	159	379
	6	4000.00	2628.19	0.80	85	193	470
**M3**	5	3000.00	2628.19	0.06	69	153	387
	6	4000.00	2628.19	0.52	85	186	499
Example 1c (H = 12h)							
**V&S**	6	4000.00	3463.62	0.16	90	155	410
	7	4951.24	3463.62	1.67	107	182	487
**M1**	6	4000.00	3463.62	0.19	85	186	481
	7	4951.24	3463.62	2.22	101	219	575
**M2**	6	4000.00	3463.62	0.16	85	193	470
	7	4951.24	3463.62	2.52	101	227	561
**M3**	6	4000.00	3463.62	0.17	85	186	499
	7	4951.24	3463.62	2.66	101	219	596
Example 1d (H = 16h)							
**V&S**	9	6601.65	5038.05	22.09	141	236	641
	10	6601.65	5038.05	1147.22	158	263	718
**M1**	9	6601.65	5038.05	28.89	133	285	763
	10	6601.65	5038.05	1248.17	149	318	857
**M2**	9	6601.65	5038.05	39.38	133	295	743
	10	6601.65	5038.05	544.03	149	329	834
**M3**	9	6601.65	5038.05	29.50	133	285	790
	10	6601.65	5038.05	1547.14	149	318	887
Example 2a (H = 8h)							
**V&S**	4	1730.87	1498.57	0.05	122	218	603
	5	2123.32	1498.57	0.44	160	279	794
**M1**	4	1730.87	1498.57	0.06	104	392	569
	5	2123.32	1498.57	0.44	136	513	758
**M2**	4	1730.87	1498.57	0.16	104	240	553
	5	2123.32	1498.57	0.60	136	309	732
**M3**	4	1730.87	1498.57	0.08	104	392	569
	5	2123.32	1498.57	0.61	136	513	758
Example 2b (H = 10h)							
**V&S**	5	2436.69	1962.70	0.17	160	279	794
	6	2730.66	1962.70	6.39	198	340	985
**M1**	5	2436.69	1962. 70	0.25	136	513	758
	6	2730.66	1962.70	7.03	168	634	947
**M2**	5	2436.69	1962. 70	0.38	136	309	732
	6	2730.66	1962.70	9.83	168	378	911
**M3**	5	2436.69	1962. 70	0.20	136	513	758
	6	2730.66	1962.70	6.59	168	634	947

			Example 2c (H = 12h)				
**V&S**	6	3076.62	2658.52	0.52	198	340	985
	7	3301.03	2658.52	14.70	236	401	1176
**M1**	6	3076.62	2658. 52	0.45	168	634	947
	7	3301.03	2658.52	14.77	200	755	1136
**M2**	6	3076.62	2658. 52	0.58	168	378	911
	7	3301.03	2658.52	11.84	200	447	1090
**M3**	6	3076.62	2658. 52	0.50	168	634	947
	7	3301.03	2658.52	15.84	200	755	1136
			Example 2d (H = 16h)				
**V&S**	8	4291.68	3738.38	25.50	274	462	1367
	9	4438.96	3738.38	989.77	312	523	1558
**M1**	8	4291.68	3738.38	25.08	232	876	1325
	9	4438.96	3738.38	968.66	264	997	1514
**M2**	8	4291.68	3738.38	24.50	232	516	1269
	9	4438.96	3738.38	1570.70	264	585	1448
**M3**	8	4291.68	3738.38	29.14	232	876	1325
	9	4438.96	3738.38	1051.75	264	997	1514
			Example 3a (H = 8h)				
**V&S**	5	2100.00	1583.44	2.38	259	385	1210
	6	2750.96	1583.44	1573.77	321	469	1502
**M1**	5	2100.00	1583.44	1.48	211	745	1237
	6	2750.96	1583.44	351.20	261	921	1546
**M2**	5	2100.00	1583.44	1.89	211	436	1199
	6	2750.96	1583.44	328.47	261	533	1494
**M3**	5	2100.00	1583.44	1.27	211	745	1232
	6	2750.96	1583.44	271.97	261	921	1540
			Example 3b (H = 10h)				
**V&S**	7	3369.69	2358.20	1068.02	383	553	1794
	8	3618.64	2358.20[a]	3600	445	637	2086
**M1**	7	3369.69	2358.20	235.24	311	1097	1855
	8	3618.64	2358.20[b]	3600	361	1273	2164
**M2**	7	3369.69	2358.20	373.91	311	630	1789
	8	3618.64	2358.20[c]	3600	361	727	2084
**M3**	7	3369.69	2358.20	398.58	311	1097	1848
	8	3618.64	2358.20[d]	3600	361	1273	2156
			Example 3c (H = 12h)				
**V&S**	7	3465.63	3041.27	2.28	383	553	1794
	8	3988.41	3041.27	9.70	445	637	2086
**M1**	7	3465.63	3041.27	1.81	311	1097	1855
	8	3988.41	3041.27	8.08	361	1273	2164
**M2**	7	3465.63	3041.27	1.34	311	630	1789
	8	3988.41	3041.27	6.45	361	727	2084
**M3**	7	3465.63	3041. 27	0.58	311	1097	1848
	8	3988.41	3041.27	16.81	361	1273	2156
			Example 3d (H = 16h)				

**V&S**	10	5225.86	4262.80	123.56	569	805	2670
	11	5644.59	4262.80[e]	3600	631	889	2962
**M1**	10	5225.86	4262.80	97.81	461	1625	2782
	11	5644.59	4262.80[f]	3600	511	1801	3091
**M2**	10	5225.86	4262.80	116.53	461	921	2674
	11	5644.59	4262.80[g]	3600	511	1018	2969
**M3**	10	5225.86	4262.80	102.97	461	1625	2772
	11	5644.59	4262.80[h]	3600	511	1801	3080

Relative gap, [a]: 1.43%, [b]: 0.15%, [c]: 0.91%, [d]: 0.15%, [e]: 1.06%, [f]: 0.83%, [g]: 5.36%, [h]: 4.66%.

Table S37. Computational results for Examples 4-12 maximizing profit with FIS

Model	Event points	RMILP	MILP	CPU time (s)	Binary variables	Continuous variables	Constraints
			Example 4 (H = 15h)				
**V&S**	6	7.50	5.32	0.17	135	191	573
	7	9.92	5.32	0.50	161	225	683
**M1**	6	7.50	5.32	0.28	120	237	710
	7	9.76	5.32	1.06	143	280	851
**M2**	6	7.50	5.32	0.20	120	245	680
	7	9.76	5.32	0.72	143	289	813
**M3**	6	7.50	5.32	0.22	120	237	722
	7	9.76	5.32	0.83	143	280	865
			Example 5 (H = 6h)				
**V&S**	5	14.00	10.00	0.06	68	113	308
	6	16.00	10.00	0.17	84	137	380
**M1**	5	14.00	10.00	0.08	64	138	368
	6	15.80	10.00	0.16	79	168	458
**M2**	5	14.00	10.00	0.03	64	144	360
	6	15.80	10.00	0.16	79	175	447
**M3**	5	14.00	10.00	0.05	64	138	378
	6	15.80	10.00	0.05	79	168	470
			Example 6 (H = 9h)				
**V&S**	5	300.00	180.00	0.17	97	160	425
	5($\Delta n = 1$)	300.00	210.00	0.19	117	180	505
	6	360.00	180.00	0.30	120	194	525
**M1**	5	300.00	210.00	0.06	89	194	509
	6	354.00	210.00	0.39	110	236	634
**M2**	5	300.00	210.00	0.06	89	196	493
	6	354.00	210.00	0.27	110	238	612
**M3**	5	300.00	210.00	0.17	89	194	524
	6	354.00	210.00	0.41	110	236	652
			Example 7 (H = 76h)				
**V&S**	5	80.00	58.99	0.06	102	170	462
	6	87.73	58.99	0.27	126	206	570
**M1**	5	80.00	58.99	0.06	94	203	544

	6	87.73	58.99	0.19	116	247	677
**M2**	5	80.00	58.99	0.14	94	214	536
	6	87.73	58.99	0.25	116	260	666
**M3**	5	80.00	58.99	0.05	94	203	559
	6	87.73	58.99	0.28	116	247	695
			Example 8 (H = 10h)				
**V&S**	6	400.00	**200.13**	0.39	84	149	432
	6($\Delta n = 1$)	400.00	**200.13**	1.06	104	169	512
	6($\Delta n = 2$)	400.00	**300.00**	0.41	120	185	560
	6($\Delta n = 3$)	400.00	**400.00**	0.03	132	197	596
	7	500.00	200.13	1.28	100	175	514
**M1**	6	400.00	400.00	0.16	79	185	536
	7	500.00	400.00	0.30	94	218	641
**M2**	6	400.00	400.00	0.05	79	209	536
	7	500.00	400.00	0.27	94	246	641
**M3**	6	400.00	400.00	0.05	79	185	536
	7	500.00	400.00	0.41	94	218	641
			Example 9 (H = 10h)				
**V&S**	10	400.00	**200.13**	9.05	148	253	760
	10($\Delta n = 1$)	400.00	**200.13**	173.88	184	289	904
	10($\Delta n = 2$)	400.00	**200.13**	455.14	216	321	1000
	10($\Delta n = 3$)	400.00	**200.13**	1709.89	244	349	1084
	10($\Delta n = 4$)	400.00	**250.00**	243.94	268	373	1156
	10($\Delta n = 5$)	400.00	**300.00**	149.22	288	393	1216
	10($\Delta n = 6$)	400.00	**350.00**	21.70	304	409	1264
	10($\Delta n = 7$)	400.00	**400.00**	2.84	316	421	1300
	11	450.00	**200.13**	15.42	164	279	842
**M1**	10	400.00	400.00	0.39	139	317	956
	11	450.00	400.00	1.58	154	350	1061
**M2**	10	400.00	400.00	0.27	139	357	956
	11	450.00	400.00	2.53	154	394	1061
**M3**	10	400.00	400.00	0.63	139	317	956
	11	450.00	400.00	1.47	154	350	1061
			Example 10 (H = 6h)				
**V&S**	2	650.00	650.00	0.06	21	60	114
	3	675.00	650.00	0.05	36	92	200
**M1**	2	650.00	650.00	0.05	21	74	100
	3	675.00	650.00	0.05	36	124	185
**M2**	2	650.00	650.00	0.03	21	62	100
	3	675.00	650.00	0.03	36	97	185
**M3**	2	650.00	650.00	0.05	21	74	100
	3	675.00	650.00	0.05	36	124	185
			Example 11 (H = 4h)				
**V&S**	7	961.62	386.000	3.53	157	254	683
	8	961.62	386.000	15.22	182	292	792

**M1**	7	961.62	386.000	10.06	139	336	718
	8	961.62	386.000	49.78	161	388	836
**M2**	7	961.62	386.000	6.34	139	272	694
	8	961.62	386.000	56.78	161	313	807
**M3**	7	961.62	386.000	6.47	139	336	739
	8	961.62	386.000	43.75	161	388	860
			Example 12 (H = 12h)				
**V&S**	8	887.68	887.68	0.28	445	637	2086
	9	1087.68	989.03	2.34	507	721	2378
	10	1795.48	989.03[a]	3600	569	805	2670
	11	2208.35	989.03[b]	3600	631	889	2962
**M1**	8	887.68	887.68	0.27	361	1273	2164
	9	1087.68	1065.63	3.70	411	1449	2473
	10	1795.48	1184.48	126.86	461	1625	2782
	11	2208.35	1201.39[c]	3600	511	1801	3091
**M2**	8	887.68	887.68	0.24	361	727	2084
	9	1087.68	1065.63	5.39	411	824	2379
	10	1795.48	1184.48	194.42	461	921	2674
	11	2208.35	1201.39[d]	3600	511	1018	2969
**M3**	8	887.68	887.68	0.27	361	1273	2156
	9	1087.68	1065.63	3.77	411	1449	2464
	10	1795.48	1184.48	193.22	461	1625	2772
	11	2208.35	1201.39[e]	3600	511	1801	3080

Relative gap, [a]: 0.17%, [b]: 1.29%, [c]: 5.98%, [d]: 5.89%, [e]: 5.36%.

Table S38. Computational results for Examples 1-3 minimizing Makespan with UIS

Model	Event points	RMILP	MILP	CPU time (s)	Binary variables	Continuous variables	Constraints
			Example 1a (D_{S4}=2000 mu)				
**V&S**	14	24.24	27.88	1616.41	122	319	667
	15	24.24	27.88[a]	3600	131	342	716
**M1**	14	24.24	27.88	178.78	122	359	868
	15	24.24	27.88[b]	3600	131	385	933
**M2**	14	24.24	27.88	160.30	122	348	812
	15	24.24	27.88	2843.73	131	373	872
**M3**	14	24.24	27.88	257.55	122	359	938
	15	24.24	27.88[c]	3600	131	385	1008
			Example 1b (D_{S4}=4000 mu)				
**V&S**	23	48.47	52.07	1015.83	203	526	1108
	24	48.47	52.07[d]	3600	212	549	1157
	25	48.47	52.07[e]	3600	221	572	1206
**M1**	23	48.47	52.07	820.34	203	593	1453
	24	48.47	52.07[f]	3600	212	619	1518
	25	48.47	52.07[g]	3600	221	645	1583
**M2**	23	48.47	52.07	484.47	203	573	1352

	24	48.47	52.07[h]	3600	212	598	1412
	25	48.47	52.07[i]	3600	221	623	1472
**M3**	23	48.47	52.07	951.56	203	593	1568
	24	48.47	52.07[j]	3600	212	619	1638
	25	48.47	52.07[k]	3600	221	645	1708
Example 2a (D_{S8}=200 mu, D_{S9}=200 mu)							
**V&S**	9	18.68	19.34	2.48	152	411	887
	10	18.68	19.34	1.28	170	458	993
**M1**	9	18.68	19.34	1.70	136	613	1009
	10	18.68	19.34	1.06	152	686	1133
**M2**	9	18.68	19.34	1.33	136	377	912
	10	18.68	19.34	2.66	152	420	1021
**M3**	9	18.68	19.34	1.41	136	613	1045
	10	18.68	19.34	1.56	152	686	1173
Example 2b (D_{S8}=500 mu, D_{S9}=400 mu)							
**V&S**	20	45.57	46.11	6.50	350	928	2053
	21	45.57	46.11	3.33	368	975	2159
**M1**	20	45.57	46.11	3.09	312	1416	2373
	21	45.57	46.11	2.63	328	1489	2497
**M2**	20	45.57	46.11	2.95	312	850	2111
	21	45.57	46.11	2.09	328	893	2220
**M3**	20	45.57	46.11	1.97	312	1416	2453
	21	45.57	46.11	2.74	328	1489	2581
Example 3a (D_{S12}=100 mu, D_{S13}=200 mu)							
**V&S**	7	11.25	13.37	0.50	179	439	1014
	8	11.25	13.37	10.14	207	504	1174
**M1**	7	11.25	13.37	0.72	167	683	1230
	8	11.25	13.37	3.08	193	790	1431
**M2**	7	11.25	13.37	0.50	167	420	1144
	8	11.25	13.37	3.89	193	482	1325
**M3**	7	11.25	13.37	0.45	167	683	1272
	8	11.25	13.37	2.99	193	790	1479
Example 3b (D_{S12}=250 mu, D_{S13}=250 mu)							
**V&S**	10	14.27	17.03	0.61	263	634	1494
	11	14.27	17.03	4.91	291	699	1654
**M1**	10	14.27	17.03	0.81	245	1004	1833
	11	14.27	17.03	4.13	271	1111	2034
**M2**	10	14.27	17.03	0.70	245	606	1687
	11	14.27	17.03	3.28	271	668	1868
**M3**	10	14.27	17.03	1.00	145	1004	1893
	11	14.27	17.03	2.56	271	1111	2100

Relative gap, [a]: 4.73%, [b]: 0.49%, [c]: 0.54%, [d]: 1.88%, [e]: 4.34%, [f]: 0.07%, [g]: 0.87%, [h]: 0.07%, [i]: 0.46%, [j]: 0.47%, [k]: 3.39% .

Table S39. Computational results for examples 1-3 minimizing Makespan with FIS

Model	Event points	RMILP	MILP	CPU time (s)	Binary variables	Continuous variables	Constraints
			Example 1a (D_{S4}=2000 mu)				
**V&S**	14	24.24	27.88[a]	3600	226	371	1031
	15	24.24	27.88[b]	3600	243	398	1108
**M1**	14	24.24	27.88	2838.75	213	450	1243
	15	24.24	27.88[c]	3600	229	483	1337
**M2**	14	24.24	27.88	3065.42	213	465	1210
	15	24.24	27.88[d]	3600	229	499	1301
**M3**	14	24.24	27.88[e]	3600	213	450	1285
	15	24.24	27.88[f]	3600	229	483	1382
			Example 1b (D_{S4}=4000 mu)				
**V&S**	24	48.47	52.07[g]	3600	396	641	1801
	25	48.47	52.07[h]	3600	413	668	1878
	26	48.47	52.07[i]	3600	430	695	1955
**M1**	24	48.47	52.07[j]	3600	373	780	2183
	25	48.47	52.07[k]	3600	389	813	2277
	26	48.47	52.07[l]	3600	405	846	2371
**M2**	24	48.47	52.24[m]	3600	373	805	2120
	25	48.47	52.07[n]	3600	389	839	2211
	26	48.47	52.07[o]	3600	405	873	2302
**M3**	24	48.47	52.07[p]	3600	373	780	2255
	25	48.47	52.07[q]	3600	389	813	2352
	26	48.47	52.07[r]	3600	405	846	2449
			Example 2a (D_{S8}=200 mu, D_{S9}=200 mu)				
**V&S**	9	18.68	19.34	2.47	312	523	1567
	10	18.68	19.34	1.74	350	584	1758
**M1**	9	18.68	19.34	2.11	264	997	1523
	10	18.68	19.34	3.73	296	1118	1712
**M2**	9	18.68	19.34	0.36	264	585	1461
	10	18.68	19.34	4.83	296	654	1640
**M3**	9	18.68	19.34	2.77	264	997	1523
	10	18.68	19.34	2.53	296	1118	1712
			Example 2b (D_{S8}=500 mu, D_{S9}=400 mu)				
**V&S**	21	47.46	47.74[s]	3600	768	1255	3859
	21($\Delta n = 1$)	47.38	47.69[t]	3600	928	1415	4499
	21($\Delta n = 2$)	47.38	47.68	1723.44	1,080	1567	4955
	22	47.46	47.73[u]	3600	806	1316	4050
**M1**	21	47.38	47.68[v]	3600	648	2449	3791
	22	47.38	47.68	17.92	680	2570	3980
**M2**	21	47.38	47.68	350.52	648	1413	3609
	22	47.38	47.68[w]	3600	680	1482	3788
**M3**	21	47.38	47.68	20.58	648	2449	3791
	22	47.38	47.68	171.81	680	2570	3980

		Example 3a (D_{S12}=100 mu, D_{S13}=200 mu)					
**V&S**	7	11.25	13.37	0.86	383	553	1806
	8	11.25	13.37	16.39	445	637	2098
**M1**	7	11.25	13.37	1.20	311	1097	1868
	8	11.25	13.37	14.19	361	1273	2177
**M2**	7	11.25	13.37	1.98	311	630	1809
	8	11.25	13.37	8.33	361	727	2104
**M3**	7	11.25	13.37	1.28	311	1097	1861
	8	11.25	13.37	8.61	361	1273	2169
		Example 3b (D_{S12}=250 mu, D_{S13}=250 mu)					
**V&S**	10	14.27	17.03	1.28	569	805	2682
	11	14.27	17.03	5.67	631	889	2974
**M1**	10	14.27	17.03	1.98	461	1625	2795
	11	14.27	17.03	5.75	511	1801	3104
**M2**	10	14.27	17.03	1.80	461	921	2694
	11	14.27	17.03	4.09	511	1018	2989
**M3**	10	14.27	17.03	1.56	461	1625	2785
	11	14.27	17.03	7.23	511	1801	3093

Relative gap, [a]: 0.21%, [b]: 4.75%, [c]: 6.82%, [d]: 8.59%, [e]: 1.08%, [f]: 5.79%, [g]: 0.70%, [h]: 3.72%, [i]: 5.56%, [j]: 2.91%, [k]: 4.29%, [l]: 6.91%, [m]: 4.71%, [n]: 5.59%, [o]: 6.91%, [p]: 2.83%, [q]: 6.37%, [r]: 6.91%, [s]: 0.01%, [t]: 0.65%, [u]: 0.07%, [v]: 0.16%, [w]: 0.12%.

Table S40. Computational results for Motivating Examples

Model	Event points/H	RMILP	MILP	CPU time (s)	Binary variables	Continuous variables	Constraints
		Motivating Example 1 (H = 8h)					
**V&S**[17]	3	500.00	300.00	0.05	12	32	66
**L&F**[14]	3	500.00	300.00	0.13	6	29	41
**SLK2**[10]	4	500.00	300.00	0.05	12	81	94
**M1**	3	500.00	500.00	0.05	12	37	72
**M2**	3	500.00	500.00	0.05	12	40	72
**M3**	3	500.00	500.00	0.03	12	37	75
		Motivating Example 2 (D_{S3}=100 mu)					
**V&S**[17]	3	5.00	11.5	0.02	12	32	68
**L&F**[14]	3	5.00	11.5	0.02	6	29	43
**SLK2**[10]	4	5.00	11.5	0.02	12	83	99
**VMM**[32]	100[a]	5.00	11.5	0.23	404	1006	2822
**M1**	3	8.00	**8**	0.02	12	37	76
**M2**	3	8.00	**8**	0.03	12	41	81
**M3**	3	8.00	**8**	0.02	12	37	79
		Motivating Example 3 (D_{P1}=100 mu, D_{P2}=200 mu)					
**V&S**[17]	10	27.00	27.00	0.05	114	350	898
	11	19.00	26.00	0.19	126	386	994
	12	19.00	25.00	0.20	138	422	1090
	18	19.00	25.00	44.5	210	638	1666

	18($\Delta n = 1$)	19.00	24.00[b]	3600	312	740	2074
	18($\Delta n = 2$)	19.00	23.00[c]	3600	408	836	2362
	18($\Delta n = 3$)	19.00	22.00	2565	498	926	2632
	18($\Delta n = 4$)	19.00	21.00	1403	582	1010	2884
	18($\Delta n = 5$)	19.00	20.00	459	660	1088	3118
	18($\Delta n = 6$)	19.00	19.00	20.7	732	1160	3334
**L&F**[14]	10	28.00	28.00	0.08	60	262	448
	11	19.00	26.00	0.20	66	288	494
	12	19.00	25.00	0.50	72	314	540
	13	19.00	24.00	0.73	78	340	586
	14	19.00	23.00	1.89	84	366	632
	15	19.00	22.00	2.20	90	392	678
	16	19.00	21.00	2.52	96	418	724
	17	19.00	20.00	2.02	102	444	770
	**18**	19.00	19.00	0.41	108	470	816
**SLK2**[10]	11	28.00	28.00	0.05	80	534	733
	12	19.00	19.00	0.19	88	584	805
**VMM**[32]	100[d]	10.00	19.00	0.86	612	1415	3256
**M1**	**10**	19.00	19.00	0.02	114	479	803
**M2**	**10**	19.00	19.00	0.03	114	403	856
**M3**	**10**	19.00	19.00	0.05	114	479	773

[a]: step size: $\delta = 0.5$, Relative gap, [b]: 3.33%, [c]: 4.35%, [d]: step size: $\delta = 0.5$.

Table S41. Computational results for Examples 1-3 maximizing profit with UIS

Model	Event points	RMILP	MILP	CPU time (s)	Binary variables	Continuous variables	Constraints
			Example 1a (H = 8h)				
**V&S**	4	2000.00	1840.17	0.03	32	89	172
	5	3000.00	1840.17	0.16	41	112	221
**M1**	4	2000.00	1840.17	0.05	32	99	208
	5	3000.00	1840.17	0.19	41	125	273
**M2**	4	2000.00	1840.17	0.03	32	98	200
	5	3000.00	1840.17	0.16	41	123	260
**M3**	4	2000.00	1840.17	0.03	32	99	228
	5	3000.00	1840.17	0.16	41	125	298
			Example 1b (H = 10h)				
**V&S**	5	3000.00	2628.19	0.05	41	112	221
	6	4000.00	2628.19	0.72	50	135	270
**M1**	5	3000.00	2628.19	0.05	41	125	273
	6	4000.00	2628.19	0.28	50	151	338
**M2**	5	3000.00	2628.19	0.03	41	123	260
	6	4000.00	2628.19	0.30	50	148	320
**M3**	5	3000.00	2628.19	0.05	41	125	298
	6	4000.00	2628.19	0.28	50	151	368
			Example 1c (H = 12h)				

**V&S**	6	4000.00	3463.62	0.16	50	135	270
	7	4951.24	3463.62	1.03	59	158	319
**M1**	6	4000.00	3463.62	0.06	50	151	338
	7	4951.24	3463.62	0.50	59	177	403
**M2**	6	4000.00	3463.62	0.08	50	148	320
	7	4951.24	3463.62	0.41	59	173	380
**M3**	6	4000.00	3463.62	0.06	50	151	368
	7	4951.24	3463.62	1.16	59	177	438
			Example 1d (H = 16h)				
**V&S**	9	6601.65	5038.05	12.78	77	204	417
	10	6601.65	5038.05	353.55	86	227	466
**M1**	9	6601.65	5038.05	4.69	77	229	533
	10	6601.65	5038.05	66.88	86	255	598
**M2**	9	6601.65	5038.05	5.28	77	223	500
	10	6601.65	5038.05	84.09	86	248	560
**M3**	9	6601.65	5038.05	8.78	77	229	578
	10	6601.65	5038.05	114.75	86	255	648
			Example 2a (H = 8h)				
**V&S**	4	1730.87	1498.57	0.05	62	176	348
	5	2123.32	1498.57	0.30	80	223	454
**M1**	4	1730.87	1498.57	0.05	56	248	380
	5	2123.32	1498.57	0.30	72	321	504
**M2**	4	1730.87	1498.57	0.05	56	162	354
	5	2123.32	1498.57	0.38	72	205	463
**M3**	4	1730.87	1498.57	0.06	56	248	396
	5	2123.32	1498.57	0.28	72	321	524
			Example 2b (H = 10h)				
**V&S**	5	2436.69	1962.69	0.16	80	223	454
	6	2730.66	1962.69	4.25	98	270	560
**M1**	5	2436.69	1962.69	0.16	72	321	504
	6	2730.66	1962.69	3.39	88	394	628
**M2**	5	2436.69	1962.69	0.17	72	205	463
	6	2730.66	1962.69	3.17	88	248	572
**M3**	5	2436.69	1962.69	0.17	72	321	524
	6	2730.66	1962.69	3.28	88	394	652
			Example 2c (H = 12h)				
**V&S**	6	3076.62	2658.52	0.27	98	270	560
	7	3301.03	2658.52	7.08	116	317	666
**M1**	6	3076.62	2658.52	0.38	88	394	628
	7	3301.03	2658.52	7.52	104	467	752
**M2**	6	3076.62	2658.52	0.27	88	248	572
	7	3301.03	2658.52	6.03	104	291	681
**M3**	6	3076.62	2658.52	0.30	88	394	652
	7	3301.03	2658.52	5.61	104	467	780
			Example 2d (H = 16h)				
**V&S**	8	4291.68	3738.38	10.99	134	364	772

	9	4438.96	3738.38	736.02	152	411	878
**M1**	8	4291.68	3738.38	10.88	120	540	876
	9	4438.96	3738.38	417.88	136	613	1000
**M2**	8	4291.68	3738.38	10.56	120	334	790
	9	4438.96	3738.38	526.95	136	377	899
**M3**	8	4291.68	3738.38	10.27	120	540	908
	9	4438.96	3738.38	553.38	136	613	1036
Example 3a (H = 8h)							
**V&S**	5	2100.00	1583.44	1.13	123	309	682
	6	2750.96	1583.44	721.06	151	374	842
**M1**	5	2100.00	1583.44	0.80	115	469	815
	6	2750.96	1583.44	41.95	141	576	1016
**M2**	5	2100.00	1583.44	0.83	115	296	762
	6	2750.96	1583.44	72.64	141	358	943
**M3**	5	2100.00	1583.44	1.56	115	469	845
	6	2750.96	1583.44	75.58	141	576	1052
Example 3b (H = 10h)							
**V&S**	7	3369.69	2358.20	514.89	179	439	1002
	8	3618.64	2358.20[a]	3600	207	504	1162
**M1**	7	3369.69	2358.20	70.05	167	683	1217
	8	3618.64	2358.20	2476.33	193	790	1418
**M2**	7	3369.69	2358.20	114.81	167	420	1124
	8	3618.64	2358.20[b]	3600	193	482	1305
**M3**	7	3369.69	2358.20	147.64	167	683	1259
	8	3618.64	2358.20[c]	3600	193	790	1466
Example 3c (H = 12h)							
**V&S**	7	3465.63	3041.27	0.91	179	439	1002
	8	3988.41	3041.27	4.86	207	504	1162
**M1**	7	3465.63	3041.27	0.39	167	683	1217
	8	3988.41	3041.27	10.49	193	790	1418
**M2**	7	3465.63	3041.27	0.80	167	420	1124
	8	3988.41	3041.27	7.17	193	482	1305
**M3**	7	3465.63	3041.27	0.47	167	683	1259
	8	3988.41	3041.27	7.36	193	790	1466
Example 3d (H = 16h)							
**V&S**	10	5225.86	4262.80	49.53	263	634	1482
	11	5644.59	4262.80[d]	3600	291	699	1642
**M1**	10	5225.86	4262.80	42.25	245	1004	1820
	11	5644.59	4262.80	2066.67	271	1111	2021
**M2**	10	5225.86	4262.80	62.02	245	606	1667
	11	5644.59	4262.80	2125.45	271	668	1848
**M3**	10	5225.86	4262.80	101.70	245	1004	1880
	11	5644.59	4262.80[e]	3600	271	1111	2087

Relative gap, [a]: 0.16%, [b]: 0.09%, [b]: 0.08%, [d]: 0.09%, [e]: 0.03%.

Table S42. Computational results for Examples 4-9 maximizing profit with UIS

Model	Event points	RMILP	MILP	CPU time (s)	Binary variables	Continuous variables	Constraints
			Example 4 (H = 15h)				
**V&S**	6	7.50	5.32	0.05	65	156	338
	7	9.92	5.32	0.27	77	183	401
**M1**	6	7.50	5.32	0.05	65	182	479
	7	9.74	5.32	0.14	77	214	573
**M2**	6	7.50	5.32	0.16	65	175	442
	7	9.74	5.32	0.17	77	205	526
**M3**	6	7.50	5.32	0.05	65	182	509
	7	9.74	5.32	0.16	77	214	608
			Example 5 (H = 6h)				
**V&S**	5	14.00	10.00	0.03	36	97	196
	6	16.00	10.00	0.06	44	117	240
**M1**	5	14.00	10.00	0.05	36	110	254
	6	15.79	10.00	0.05	44	133	315
**M2**	5	14.00	10.00	0.03	36	108	241
	6	15.79	10.00	0.03	44	130	297
**M3**	5	14.00	10.00	0.05	36	110	274
	6	15.79	10.00	0.06	44	133	339
			Example 6 (H = 9h)				
**V&S**	5	300.00	210.00	0.05	49	136	265
	6	360.00	210.00	0.17	60	164	325
**M1**	5	300.00	210.00	0.05	49	154	353
	6	353.68	210.00	0.17	60	186	438
**M2**	5	300.00	210.00	0.03	49	148	331
	6	353.68	210.00	0.16	60	178	408
**M3**	5	300.00	210.00	0.05	49	154	378
	6	353.68	210.00	0.16	60	186	468
			Example 7 (H = 76h)				
**V&S**	5	80.00	58.99	0.03	54	146	294
	6	87.73	58.99	0.25	66	176	360
**M1**	5	80.00	58.99	0.05	54	163	375
	6	87.73	58.99	0.16	66	197	465
**M2**	5	80.00	58.99	0.03	54	162	360
	6	87.73	58.99	0.14	66	195	444
**M3**	5	80.00	58.99	0.05	54	163	405
	6	87.73	58.99	0.16	66	197	501
			Example 8 (H = 10h)				
**V&S**	6	400.00	400.00	0.03	44	129	272
	7	500.00	400.00	0.16	52	151	322
**M1**	6	400.00	400.00	0.05	44	150	361
	7	500.00	400.00	0.19	52	176	431
**M2**	6	400.00	400.00	0.11	44	154	349
	7	500.00	400.00	0.17	52	180	415

**M3**	6	400.00	400.00	0.05	44	150	385
	7	500.00	400.00	0.17	52	176	459
			Example 9 (H = 10h)				
**V&S**	10	400.00	400.00	0.06	76	217	472
	11	450.00	400.00	0.30	84	239	522
**M1**	10	400.00	400.00	0.03	76	254	641
	11	450.00	400.00	0.28	84	280	711
**M2**	10	400.00	400.00	0.05	76	258	613
	11	450.00	400.00	0.27	84	284	679
**M3**	10	400.00	400.00	0.05	76	254	681
	11	450.00	400.00	0.28	84	280	755

Table S43. Computational results for Examples 1-3 maximizing profit with FIS

Model	Event points	RMILP	MILP	CPU time (s)	Binary variables	Continuous variables	Constraints
			Example 1a (H = 8h)				
**V&S**	4	2000.00	1840.17	0.03	56	101	256
	5	3000.00	1840.17	0.17	73	128	333
**M1**	4	2000.00	1840.17	0.03	53	120	293
	5	3000.00	1840.17	0.16	69	153	387
**M2**	4	2000.00	1840.17	0.03	53	125	288
	5	3000.00	1840.17	0.17	69	159	379
**M3**	4	2000.00	1840.17	0.05	53	120	305
	5	3000.00	1840.17	0.17	69	153	402
			Example 1b (H = 10h)				
**V&S**	5	3000.00	2628.19	0.05	73	128	333
	6	4000.00	2628.19	0.94	90	155	410
**M1**	5	3000.00	2628.19	0.06	69	153	377
	6	4000.00	2628.19	0.61	85	186	481
**M2**	5	3000.00	2628.19	0.05	69	159	379
	6	4000.00	2628.19	0.58	85	193	470
**M3**	5	3000.00	2628.19	0.06	69	153	387
	6	4000.00	2628.19	0.52	85	186	499
			Example 1c (H = 12h)				
**V&S**	6	4000.00	3463.62	0.17	90	155	410
	7	4951.24	3463.62	1.34	107	182	487
**M1**	6	4000.00	3463.62	0.17	85	186	481
	7	4951.24	3463.62	1.70	101	219	575
**M2**	6	4000.00	3463.62	0.14	85	193	470
	7	4951.24	3463.62	2.39	101	227	561
**M3**	6	4000.00	3463.62	0.17	85	186	499
	7	4951.24	3463.62	2.66	101	219	596
			Example 1d (H = 16h)				
**V&S**	9	6601.65	5038.05	17.86	141	236	641
	10	6601.65	5038.05	607.30	158	263	718

**M1**	9	6601.65	5038.05	26.17	133	285	763
	10	6601.65	5038.05	867.92	149	318	857
**M2**	9	6601.65	5038.05	22.95	133	295	743
	10	6601.65	5038.05	1679.91	149	329	834
**M3**	9	6601.65	5038.05	35.55	133	285	790
	10	6601.65	5038.05	1602.11	149	318	887
			Example 2a (H = 8h)				
**V&S**	4	1730.87	1498.57	0.05	122	218	603
	5	2123.32	1498.57	0.41	160	279	794
**M1**	4	1730.87	1498.57	0.05	104	392	569
	5	2123.32	1498.57	0.44	136	513	758
**M2**	4	1730.87	1498.57	0.16	104	240	553
	5	2123.32	1498.57	0.67	136	309	732
**M3**	4	1730.87	1498.57	0.06	104	392	569
	5	2123.32	1498.57	0.50	136	513	758
			Example 2b (H = 10h)				
**V&S**	5	2436.69	1962.70	0.17	160	279	794
	6	2730.66	1962.70	5.92	198	340	985
**M1**	5	2436.69	1962.70	0.25	136	513	758
	6	2730.66	1962.70	6.66	168	634	947
**M2**	5	2436.69	1962.70	0.38	136	309	732
	6	2730.66	1962.70	7.13	168	378	911
**M3**	5	2436.69	1962.70	0.48	136	513	758
	6	2730.66	1962.70	8.05	168	634	947
			Example 2c (H = 12h)				
**V&S**	6	3076.62	2658.52	0.50	198	340	985
	7	3301.03	2658.52	11.05	236	401	1176
**M1**	6	3076.62	2658.52	0.52	168	634	947
	7	3301.03	2658.52	13.58	200	755	1136
**M2**	6	3076.62	2658.52	0.38	168	378	911
	7	3301.03	2658.52	14.34	200	447	1090
**M3**	6	3076.62	2658.52	0.48	168	634	947
	7	3301.03	2658.52	19.00	200	755	1136
			Example 2d (H = 16h)				
**V&S**	8	4291.68	3738.38	17.55	274	462	1367
	9	4438.96	3738.38	1155.91	312	523	1558
**M1**	8	4291.68	3738.38	24.36	232	876	1325
	9	4438.96	3738.38	1190.34	264	997	1514
**M2**	8	4291.68	3738.38	25.59	232	516	1269
	9	4438.96	3738.38	1514.14	264	585	1448
**M3**	8	4291.68	3738.38	34.20	232	876	1325
	9	4438.96	3738.38	1864.02	264	997	1514
			Example 3a (H = 8h)				
**V&S**	5	2100.00	1583.44	2.00	259	385	1210
	6	2750.96	1583.44	886.34	321	469	1502
**M1**	5	2100.00	1583.44	2.13	211	745	1237

	6	2750.96	1583.44	273.50	261	921	1546
**M2**	5	2100.00	1583.44	1.31	211	436	1199
	6	2750.96	1583.44	238.19	261	533	1494
**M3**	5	2100.00	1583.44	1.44	211	745	1232
	6	2750.96	1583.44	299.39	261	921	1540
			Example 3b (H = 10h)				
**V&S**	7	3369.69	2358.20	950.72	383	553	1794
	8	3618.64	2358.20[a]	3600	445	637	2086
**M1**	7	3369.69	2358.20	339.52	311	1097	1855
	8	3618.64	2358.20[b]	3600	361	1273	2164
**M2**	7	3369.69	2358.20	420.52	311	630	1789
	8	3618.64	2358.20[c]	3600	361	727	2084
**M3**	7	3369.69	2358.20	393.09	311	1097	1848
	8	3618.64	2358.20[d]	3600	361	1273	2156
			Example 3c (H = 12h)				
**V&S**	7	3465.63	3041.27	2.03	383	553	1794
	8	3988.41	3041.27	9.47	445	637	2086
**M1**	7	3465.63	3041.27	1.14	311	1097	1855
	8	3988.41	3041.27	3.38	361	1273	2164
**M2**	7	3465.63	3041.27	1.531	311	630	1789
	8	3988.41	3041.27	6.84	361	727	2084
**M3**	7	3465.63	3041.27	2.83	311	1097	1848
	8	3988.41	3041.27	19.06	361	1273	2156
			Example 3d (H = 16h)				
**V&S**	10	5225.86	4262.80	98.31	569	805	2670
	11	5644.59	4262.80[e]	3600	631	889	2962
**M1**	10	5225.86	4262.80	83.63	461	1625	2782
	11	5644.59	4262.80[f]	3600	511	1801	3091
**M2**	10	5225.86	4262.80	69.23	461	921	2674
	11	5644.59	4262.80[g]	3600	511	1018	2969
**M3**	10	5225.86	4262.80	66.97	461	1625	2772
	11	5644.59	4262.80[h]	3600	511	1801	3080

Relative gap, [a]: 1.99%, [b]: 0.15%, [c]: 0.15%, [d]: 0.15%, [e]: 1.41%, [f]: 0.83%, [g]: 3.11%, [h]: 0.83%.

Table S44. Computational results for Examples 4-11 maximizing profit with FIS

Model	Event points	RMILP	MILP	CPU time (s)	Binary variables	Continuous variables	Constraints
			Example 4 (H = 15h)				
**V&S**	6	7.50	5.32	0.08	135	191	573
	7	9.92	5.32	0.41	161	225	683
**M1**	6	7.50	5.32	0.20	120	237	710
	7	9.76	5.32	0.48	143	280	851
**M2**	6	7.50	5.32	0.28	120	245	680
	7	9.76	5.32	0.77	143	289	813
**M3**	6	7.50	5.32	0.20	120	237	722

	7	9.76	5.32	0.52	143	280	865
			Example 5 (H = 6h)				
**V&S**	5	14.00	10.00	0.06	68	113	308
	6	16.00	10.00	0.06	84	137	380
**M1**	5	14.00	10.00	0.05	64	138	368
	6	15.80	10.00	0.17	79	168	458
**M2**	5	14.00	10.00	0.03	64	144	360
	6	15.80	10.00	0.16	79	175	447
**M3**	5	14.00	10.00	0.05	64	138	378
	6	15.80	10.00	0.06	79	168	470
			Example 6 (H = 9h)				
**V&S**	5	300.00	180.00	0.17	97	160	425
	$5(\Delta n = 1)$	300.00	210.00	0.16	117	180	505
	6	360.00	180.00	0.28	120	194	525
**M1**	5	300.00	210.00	0.05	89	194	509
	6	354.00	210.00	0.38	110	236	634
**M2**	5	300.00	210.00	0.06	89	196	493
	6	354.00	210.00	0.28	110	238	612
**M3**	5	300.00	210.00	0.16	89	194	524
	6	354.00	210.00	0.31	110	236	652
			Example 7 (H = 76h)				
**V&S**	5	80.00	58.99	0.05	102	170	462
	6	87.73	58.99	0.27	126	206	570
**M1**	5	80.00	58.99	0.05	94	203	544
	6	87.73	58.99	0.27	116	247	677
**M2**	5	80.00	58.99	0.13	94	214	536
	6	87.73	58.99	0.16	116	260	666
**M3**	5	80.00	58.99	0.03	94	203	559
	6	87.73	58.99	0.16	116	247	695
			Example 8 (H = 10h)				
**V&S**	6	400.00	200.13	0.38	84	149	432
	$6(\Delta n = 1)$	400.00	200.13	1.27	104	169	512
	$6(\Delta n = 2)$	400.00	300.00	0.38	120	185	560
	$6(\Delta n = 3)$	400.00	400.00	0.16	132	197	596
	7	500.00	200.13	0.61	100	175	514
**M1**	6	400.00	400.00	0.05	79	185	536
	7	500.00	400.00	0.28	94	218	641
**M2**	6	400.00	400.00	0.14	79	209	536
	7	500.00	400.00	0.25	94	246	641
**M3**	6	400.00	400.00	0.16	79	185	536
	7	500.00	400.00	0.27	94	218	641
			Example 9 (H = 10h)				
**V&S**	10	400.00	200.13	10.81	148	253	760
	$10(\Delta n = 1)$	400.00	200.13	173.88	184	289	904
	$10(\Delta n = 2)$	400.00	200.13	485.17	216	321	1000

	10($\Delta n = 3$)	400.00	200.13	2020.09	244	349	1084
	10($\Delta n = 4$)	400.00	250.00	127.58	268	373	1156
	10($\Delta n = 5$)	400.00	300.00	113.56	288	393	1216
	10($\Delta n = 6$)	400.00	350.00	9.86	304	409	1264
	10($\Delta n = 7$)	400.00	400.00	1.77	316	421	1300
	11	450.00	200.13	19.73	164	279	842
**M1**	10	400.00	400.00	0.16	139	317	956
	11	450.00	400.00	0.89	154	350	1061
**M2**	10	400.00	400.00	0.59	139	357	956
	11	450.00	400.00	1.75	154	394	1061
**M3**	10	400.00	400.00	0.38	139	317	956
	11	450.00	400.00	1.22	154	350	1061
			Example 10 (H = 6h)				
**V&S**	2	650.00	650.00	0.05	21	60	114
	3	675.00	650.00	0.05	36	92	200
**M1**	2	650.00	650.00	0.03	21	74	100
	3	675.00	650.00	0.03	36	124	185
**M2**	2	650.00	650.00	0.02	21	62	100
	3	675.00	650.00	0.03	36	97	185
**M3**	2	650.00	650.00	0.05	21	74	100
	3	675.00	650.00	0.03	36	124	185
			Example 11 (H = 4h)				
**V&S**	7	961.62	386.00	3.16	157	254	683
	8	961.62	386.00	6.80	182	292	792
**M1**	7	961.62	386.00	8.67	139	336	718
	8	961.62	386.00	61.14	161	388	836
**M2**	7	961.62	386.00	10.02	139	272	694
	8	961.62	386.00	46.36	161	313	807
**M3**	7	961.62	386.00	10.34	139	336	739
	8	961.62	386.00	64.08	161	388	860
			Example 12 (H = 12h)				
**V&S**	8	887.68	887.68	0.41	445	637	2086
	9	1087.68	989.03	2.09	507	721	2378
	10	1795.48	989.03	2096.16	569	805	2670
	11	2208.35	989.03[a]	3600	631	889	2962
**M1**	8	887.68	887.68	0.24	361	1273	2164
	9	1087.68	1065.63	1.78	411	1449	2473
	10	1795.48	1184.48	316.81	461	1625	2782
	11	2208.35	1201.39[b]	3600	511	1801	3091
**M2**	8	887.68	887.68	0.24	361	727	2084
	9	1087.68	1065.63	4.19	411	824	2379
	10	1795.48	1184.48	189.94	461	921	2674
	11	2208.35	1201.39[c]	3600	511	1018	2969
**M3**	8	887.68	887.68	0.23	361	1273	2156
	9	1087.68	1065.63	2.61	411	1449	2464

	10	1795.48	1184.48	125.20	461	1625	2772
	11	2208.35	1201.39[d]	3600	511	1801	3080

Relative gap, [a]: 3.23%, [b]: 5.74%, [c]: 5.08%, [d]: 6.13%.

Table S45. Computational results for Examples 1-3 minimizing makespan with UIS

Model	Event points	RMILP	MILP	CPU time (s)	Binary variables	Continuous variables	Constraints
			Example 1a (D_{S4}=2000 mu)				
**V&S**	14	24.24	27.88	833.19	122	319	667
	15	24.24	27.88[a]	3600	131	342	716
**M1**	14	24.24	27.88	133.66	122	359	868
	15	24.24	27.88	2578.17	131	385	933
**M2**	14	24.24	27.88	165.33	122	348	812
	15	24.24	27.88[b]	3600	131	373	872
**M3**	14	24.24	27.88	319.77	122	359	938
	15	24.24	27.88[c]	3600	131	385	1008
			Example 1b (D_{S4}=4000 mu)				
**V&S**	23	48.47	52.07	1163.56	203	526	1108
	24	48.47	52.07[d]	3600	212	549	1157
	25	48.47	52.07[e]	3600	221	572	1206
**M1**	23	48.47	52.07	596.95	203	593	1453
	24	48.47	52.07[f]	3600	212	619	1518
	25	48.47	52.07[g]	3600	221	645	1583
**M2**	23	48.47	52.07	449.38	203	573	1352
	24	48.47	52.07[h]	3600	212	598	1412
	25	48.47	52.07[i]	3600	221	623	1472
**M3**	23	48.47	52.07	712.75	203	593	1568
	24	48.47	52.07[j]	3600	212	619	1638
	25	48.47	52.07[k]	3600	221	645	1708
			Example 2a (D_{S8}=200 mu, D_{S9}=200 mu)				
**V&S**	9	18.68	19.34	1.03	152	411	887
	10	18.68	19.34	0.89	170	458	993
**M1**	9	18.68	19.34	1.11	136	613	1009
	10	18.68	19.34	1.03	152	686	1133
**M2**	9	18.68	19.34	1.31	136	377	912
	10	18.68	19.34	1.20	152	420	1021
**M3**	9	18.68	19.34	1.20	136	613	1045
	10	18.68	19.34	1.30	152	686	1173
			Example 2b (D_{S8}=500 mu, D_{S9}=400 mu)				
**V&S**	20	45.57	46.11	1.28	350	928	2053
	21	45.57	46.11	1.55	368	975	2159
**M1**	20	45.57	46.11	2.27	312	1416	2373
	21	45.57	46.11	2.17	328	1489	2497
**M2**	20	45.57	46.11	3600	312	850	2111
	21	45.57	46.11	3600	328	893	2220

**M3**	20	45.57	46.11	1.86	312	1416	2453
	21	45.57	46.11	1.64	328	1489	2581
		Example 3a (D_{S12}=100 mu, D_{S13}=200 mu)					
**V&S**	7	11.25	13.37	0.42	179	439	1014
	8	11.25	13.37	12.94	207	504	1174
**M1**	7	11.25	13.37	0.33	167	683	1230
	8	11.25	13.37	3.34	193	790	1431
**M2**	7	11.25	13.37	0.39	167	420	1144
	8	11.25	13.37	2.72	193	482	1325
**M3**	7	11.25	13.37	0.59	167	683	1272
	8	11.25	13.37	3.78	193	790	1479
		Example 3b (D_{S12}=250 mu, D_{S13}=250 mu)					
**V&S**	10	14.27	17.03	0.61	263	634	1494
	11	14.27	17.03	5.02	291	699	1654
**M1**	10	14.27	17.03	0.89	245	1004	1833
	11	14.27	17.03	2.91	271	1111	2034
**M2**	10	14.27	17.03	0.72	245	606	1687
	11	14.27	17.03	3.38	271	668	1868
**M3**	10	14.27	17.03	0.67	145	1004	1893
	11	14.27	17.03	3.55	271	1111	2100

Relative gap, [a]: 4.64%, [b]: 0.47%, [c]: 0.59%, [d]: 2.28%, [e]: 5.10%, [f]:0.08%, [g]:0.92%, [h]: 0.07%, [i]: 0.69%, [j]: 0.09%, [k]: 1.99%.

Table S46. Computational results for Examples 1-3 minimizing makespan with FIS

Model	Event points	RMILP	MILP	CPU time (s)	Binary variables	Continuous variables	Constraints
			Example 1a (D_{S4}=2000 mu)				
**V&S**	14	24.24	27.88[a]	3600	226	371	1031
	15	24.24	27.88[b]	3600	243	398	1108
**M1**	14	24.24	27.88	1968.70	213	450	1243
	15	24.24	27.88[c]	3600	229	483	1337
**M2**	14	24.24	27.88[d]	3600	213	465	1210
	15	24.24	27.88[e]	3600	229	499	1301
**M3**	14	24.24	27.88[f]	3600	213	450	1285
	15	24.24	27.88[g]	3600	229	483	1382
			Example 1b (D_{S4}=4000 mu)				
**V&S**	24	48.47	52.07[h]	3600	396	641	1801
	25	48.47	52.07[i]	3600	413	668	1878
	26	48.47	52.07[j]	3600	430	695	1955
**M1**	24	48.47	52.07[k]	3600	373	780	2183
	25	48.47	52.07[l]	3600	389	813	2277
	26	48.47	52.07[m]	3600	405	846	2371
**M2**	24	48.47	52.07[n]	3600	373	805	2120
	25	48.47	52.07[o]	3600	389	839	2211
	26	48.47	52.07[p]	3600	405	873	2302

**M3**	24	48.47	52.07[q]	3600	373	780	2255
	25	48.47	52.07[r]	3600	389	813	2352
	26	48.47	52.07[s]	3600	405	846	2449
Example 2a (D_{S8}=200 mu, D_{S9}=200 mu)							
**V&S**	9	18.68	19.34	1.78	312	523	1567
	10	18.68	19.34	1.58	350	584	1758
**M1**	9	18.68	19.34	1.94	264	997	1523
	10	18.68	19.34	1.84	296	1118	1712
**M2**	9	18.68	19.34	1.34	264	585	1461
	10	18.68	19.34	1.63	296	654	1640
**M3**	9	18.68	19.34	2.17	264	997	1523
	10	18.68	19.34	1.77	296	1118	1712
Example 2b (D_{S8}=500 mu, D_{S9}=400 mu)							
**V&S**	21	47.46	47.74[t]	3600	768	1255	3859
	21($\Delta n = 1$)	47.38	47.69[u]	3600	928	1415	4499
	21($\Delta n = 2$)	47.38	47.68[v]	3600	1080	1567	4955
	22	47.46	47.73[w]	3600	806	1316	4050
**M1**	21	47.38	47.68	82.02	648	2449	3791
	22	47.38	47.68[x]	3600	680	2570	3980
**M2**	21	47.38	47.68[y]	3600	648	1413	3609
	22	47.38	47.68	113.28	680	1482	3788
**M3**	21	47.38	47.68[z]	3600	648	2449	3791
	22	47.38	47.68	7.39	680	2570	3980
Example 3a (D_{S12}=100 mu, D_{S13}=200 mu)							
**V&S**	7	11.25	13.37	0.50	383	553	1806
	8	11.25	13.37	16.08	445	637	2098
**M1**	7	11.25	13.37	1.25	311	1097	1868
	8	11.25	13.37	10.77	361	1273	2177
**M2**	7	11.25	13.37	1.33	311	630	1809
	8	11.25	13.37	36.22	361	727	2104
**M3**	7	11.25	13.37	1.16	311	1097	1861
	8	11.25	13.37	26.67	361	1273	2169
Example 3b (D_{S12}=250 mu, D_{S13}=250 mu)							
**V&S**	10	14.27	17.03	0.97	569	805	2682
	11	14.27	17.03	6.97	631	889	2974
**M1**	10	14.27	17.03	1.52	461	1625	2795
	11	14.27	17.03	5.28	511	1801	3104
**M2**	10	14.27	17.03	1.73	461	921	2694
	11	14.27	17.03	4.83	511	1018	2989
**M3**	10	14.27	17.03	1.56	461	1625	2785
	11	14.27	17.03	8.84	511	1801	3093

Relative gap, [a]: 0.08%, [b]: 4.93%, [c]: 5.47%, [d]: 0.84%, [e]: 10.75%, , [f]: 2.08%, [g]: 5.36%, [h]: 2.48%, [i]: 4.97%, [j]: 5.02%, [k]: 2.06%, [l]: 6.09%, [m]: 6.41%, [n]: 3.06%, [o]: 5.73%, [p]: 6.91%, [q]: 4.15%, [r]: 4.01%, [s]: 6.91%, [t]: 0.07%, [u]: 0.65%, [v]: 0.64%, [w]: 0.07%, [x]: 0.64%, [y]: 0.60%, [z]: 0.64%.

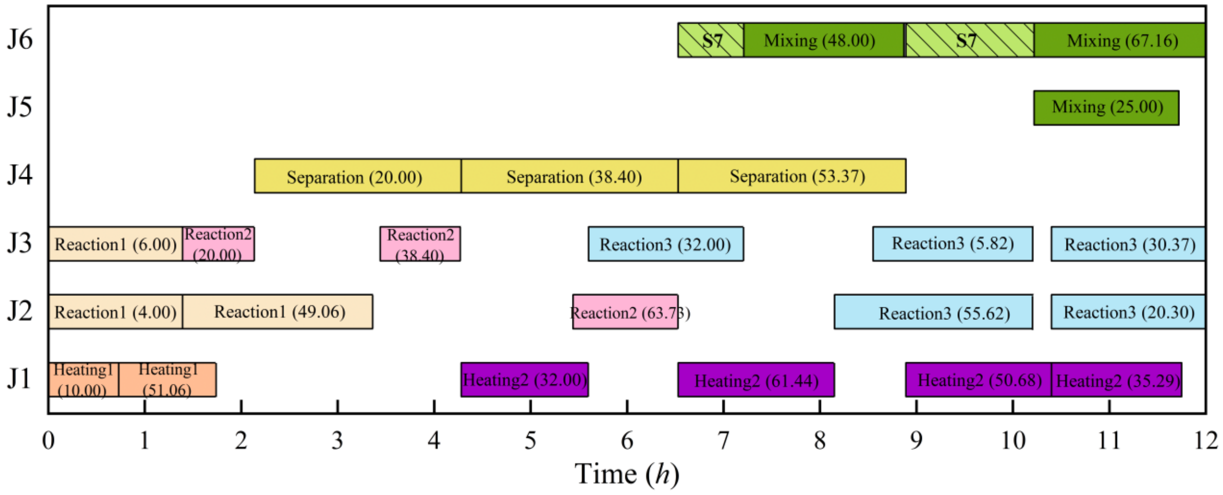

Figure S15. Optimal schedule for the Example 12 using V&S model (Profit = $989.03)
('▧' means one state waits in a unit before processing)

S4. Nomenclature

Indices

$i, i' \in \mathbf{I}$ = tasks

$j, j' \in \mathbf{J}$ = units

$s, s' \in \mathbf{S}$ = states

$n, n', n'' \in \mathbf{N}$ = event points

Sets

Mannually entered sets

$\mathbf{S}^R, \mathbf{S}^{in}, \mathbf{S}^P$ = raw material, intermediate states and products, respectively

$\mathbf{S}^{FIS}, \mathbf{S}^{UIS}, \mathbf{S}^{NIS}, \mathbf{S}^{ZW}$ = states subject to FIS, UIS, NIS and ZW, respectively

$\mathbf{I}^V$= tasks with variable recipes indicating flexible proportions for output

$\mathbf{I}_j$ = tasks that can be processed on unit *j*

Automatically determined sets

$\mathbf{I}_s$ = tasks that can produce or consume state *s*

$\mathbf{S}^B$ = states initially held in units processing production tasks of the state

$\mathbf{S}_i^{\mathrm{P}}$ = states that can be produced by task i

$\mathbf{S}_i^{\mathrm{C}}$ = tasks that can be consumed by task i

$\mathbf{I}_s^{\mathrm{P}}$ = tasks that produce state *s*

$\mathbf{I}_s^{\mathrm{C}}$ = tasks that consume state *s*

$\mathbf{I}^P$ = tasks which produce states that can be temporarily held in processing units

$\mathbf{I}^{Pzw}$ = tasks that can produce a state subject to ZW policy

$\mathbf{I}^{PF}$ = tasks that can produce a state subject to FIS policy

$\mathbf{J}_s$ = units that can perform tasks producing or consuming state *s*

$\mathbf{J}_s^P$ = units that can perform tasks producing state *s*

$\mathbf{J}_s^C$ = units that can perform tasks consuming state *s*

$\mathbf{J}^{Pzw}$ = units that can perform tasks producing a state subject to ZW policy

$\mathbf{CJ}_j$ = units where there are tasks consuming $s \in \mathbf{S}^{in}$ that is produced in the unit j.

$\mathbf{CJ}_j^U, \mathbf{CJ}_j^F, \mathbf{CJ}_j^{NIS}, \mathbf{CJ}_j^{ZW}$ = units consuming states with UIS, FIS, UIS and ZW, respectively, which are produced in the unit j.

$\mathbf{CJ0}_j$ = units $j' \in \mathbf{CJ}_j$ but excludes *j'* for which multiple states produced by tasks in j are consumed by a task in j' or multiple states consumed by tasks in j' are produced by a task in j.

Parameters

$B_{ij}^{\min}, B_{ij}^{\max}$ = minimum and maximum batch size of task i on unit j, respectively

Δn = maximum number of event points over which a task can span

ρ_{si} = proportion of state s produced (> 0) or consumed (< 0) by task i

$ST0_s$ = initial inventory of state s

$bs0_{ij}$ = initial amounts of one batch produced by task i that are held in a unit j

$bsv0_{sij}$= initial amount of state s produced by task $i \in \mathbf{I}^V$ that are held in a unit j

α_{ij}, β_{ij} = fixed and variable processing time of task i processing on unit j, respectively

M = large possible value

H = scheduling horizon

ST_s^{max} = maximum storage capacity for one state s

$\rho_{si}^{min}, \rho_{si}^{max}$ = minimum and maximum fractions of state s produced by task $i \in \mathbf{I}^V$, respectively

Binary variables

$w_{ijnn'}$ =1 if task i is processed in unit j from event point n to event point n'

ys_{ijn} =1 if a batch previously produced by task i is held in unit j at event point n

$zI_{jj'n}$ =1 if there is indirect material transfer from unit j to unit j' at event point n

$zD_{jj'n}$ =1 if there is direct material transfer from unit j to unit j' at event point n

Positive variables

$b_{ijnn'}$ = batch size of task i processed in unit j from event point n to event point n'

ST_{sn}^{M1} = excess amount of state s stored in both processing units and storage at event point n

ST_{sn}^{M2} = inventory level of state s at the beginning of an event point n

T_{jn}^{s} = start time of unit j at event point n

T_{jn}^{f} = end time of unit j at event point n

T_{sjn} = time when state s produced in unit j is available for consumption or storage at event point n

T_{sn}^{M2} = time when state s is available (for e.g. consumption or storage) at event point n

tt_{sn}^{M2} = an intermediate variable that is used to aid the sequencing between related production and consumption tasks taking place at event point n for state s with FIS policy

$bTi_{iji'j'n}$ = amount of a state produced by task i in unit j at event $(n-1)$ and indirectly transferred to unit j' where the task i' consume the state at event n

$bTd_{iji'j'n}$ = amount of a state produced by task i in unit j at event $(n-1)$ and directly

transferred to unit j' where the task i' consume the state at event n

$bv_{sijnn'}$ = amount of state s produced by task $i \in \mathbf{I}^V$ processed in unit j from event n to n'

bsv_{sijn} = amount of state s in one batch produced by task $i \in \mathbf{I}^V$ and held in unit j at event n

MS = makespan

z = profit

S5. Automatic determination of sets

Determine $\mathbf{I}_s, \mathbf{I}_s^{\mathrm{P}}$, and $\mathbf{I}_s^{C}$

For $s \in \mathbf{S}$

$\mathbf{I}_s = \emptyset, \mathbf{I}_s^{\mathrm{P}} = \emptyset, \mathbf{I}_s^{\mathrm{C}} = \emptyset$

For $i \in \mathbf{I}$

If $\rho_{si} > 0$

$\mathbf{I}_s^{\mathrm{P}} = \mathbf{I}_s^{\mathrm{P}} \cup \{i\}$

$\mathbf{I}_s = \mathbf{I}_s \cup \{i\}$

End

If $\rho_{si} < 0$

$\mathbf{I}_s^{C} = \mathbf{I}_s^{\mathrm{C}} \cup \{i\}$

$\mathbf{I}_s = \mathbf{I}_s \cup \{i\}$

End

End

End

Determine $\mathbf{S}_i^P$ *and* $\mathbf{S}_i^C$

For $i \in \mathbf{I}$

$\mathbf{S}_i^{\mathrm{P}} = \emptyset, \mathbf{S}_i^{\mathrm{C}} = \emptyset$

For $s \in \mathbf{S}$

If $\rho_{si} > 0$

$\mathbf{S}_i^{\mathrm{P}} = \mathbf{S}_i^{\mathrm{P}} \cup \{s\}$

End

If $\rho_{si} < 0$

$\mathbf{S}_i^{\mathrm{C}} = \mathbf{S}_i^{\mathrm{C}} \cup \{s\}$

End

End

End

Determine $\mathbf{S}^B$

$\mathbf{S}^B = \emptyset$

For $s \in \mathbf{S}$

For $j, i \in (\mathbf{I}_s^P \cap \mathbf{J}_i)$

If $bs0_{ij} > 0$

$\mathbf{S}^B = \mathbf{S}^B \cup \{s\}$

End

End

End

Determine $\mathbf{J}_s^{\mathrm{P}}$, and $\mathbf{J}_s^{\mathrm{C}}$

For $s \in \mathbf{S}$

$\mathbf{J}_s^{\mathrm{P}} = \emptyset, \mathbf{J}_s^{\mathrm{C}} = \emptyset$

For $j \in \mathbf{J}$

If $\exists i \in (\mathbf{I}_j \cap \mathbf{I}_s^{\mathrm{P}})$

$\mathbf{J}_s^{\mathrm{P}}$=$\mathbf{J}_s^{\mathrm{P}} \cup \{j\}$

End

If $\exists i \in (\mathbf{I}_j \cap \mathbf{I}_s^{\mathrm{C}})$

$\mathbf{J}_s^{\mathrm{C}}$=$\mathbf{J}_s^{\mathrm{C}} \cup \{j\}$

End

End

End

Determine $\mathbf{I}^{P}$, $\mathbf{I}^{PF}$, and $\mathbf{I}^{Pzw}$

$\mathbf{I}^{P} = \emptyset, \mathbf{I}^{PF} = \emptyset, \mathbf{I}^{Pzw} = \emptyset$

For $i \in \mathbf{I}$

    If $\exists s \in ((\mathbf{S}^{FIS} \cup \mathbf{S}^{NIS}) \cap \mathbf{S}_i^{\mathrm{P}})$

        $\mathbf{I}^{P} = \mathbf{I}^{P} \cup \{i\}$

      If $\exists s \in (\mathbf{S}^{ZW} \cap \mathbf{S}_i^{\mathrm{P}})$

        $\mathbf{I}^{P} = \mathbf{I}^{P} \setminus \{i\}$

      End

    End

    If $\exists s \in (\mathbf{S}^{FIS} \cap \mathbf{S}_i^{\mathrm{P}})$

        $\mathbf{I}^{PF} = \mathbf{I}^{PF} \cup \{i\}$

    End

    If $\exists s \in (\mathbf{S}^{ZW} \cap \mathbf{S}_i^{\mathrm{P}})$

        $\mathbf{I}^{Pzw} = \mathbf{I}^{Pzw} \cup \{i\}$

    End

End

Determine $\mathbf{J}_s$ *and* $\mathbf{J}^{Pzw}$

$\mathbf{J}^{Pzw} = \emptyset$

For $s \in \mathbf{S}$

  $\mathbf{J}_s = \emptyset$

  For $j \in \mathbf{J}$

    If $\exists i \in (\mathbf{I}_j \cap (\mathbf{I}_s^{P} \cup \mathbf{I}_s^{C}))$

        $\mathbf{J}_s = \mathbf{J}_s \cup \{j\}$

    End

    If $\exists i \in (\mathbf{I}_j \cap \mathbf{I}_s^{P})$

        If $s \in \mathbf{S}^{ZW}$

          $\mathbf{J}^{Pzw} = \mathbf{J}^{Pzw} \cup \{j\}$

        End

    End

  End

End

Determine $\mathbf{CJ}_j$, $\mathbf{CJ}_j^{F}$, $\mathbf{CJ}_j^{U}$, $\mathbf{CJ}_j^{NIS}$, and $\mathbf{CJ}_j^{ZW}$

For $j \in \mathbf{J}$

  $\mathbf{CJ}_j = \emptyset, \mathbf{CJ}_j^{F} = \emptyset, \mathbf{CJ}_j^{U} = \emptyset, \mathbf{CJ}_j^{NIS} = \emptyset, \mathbf{CJ}_j^{ZW} = \emptyset$

  For $j' \in \mathbf{J}$

      If $\exists s \in \mathbf{S}^{in}: j \in \mathbf{J}_s^{P}, j' \in \mathbf{J}_s^{C}$

        $\mathbf{CJ}_j = \mathbf{CJ}_j \cup \{j'\}$

      End

      If $\exists s \in \mathbf{S}^{FIS}: j \in \mathbf{J}_s^{P}, j' \in \mathbf{J}_s^{C}$

        $\mathbf{CJ}_j^{F} = \mathbf{CJ}_j^{F} \cup \{j'\}$

      End

      If $\exists s \in \mathbf{S}^{NIS}: j \in \mathbf{J}_s^{P}, j' \in \mathbf{J}_s^{C}$

        $\mathbf{CJ}_j^{NIS} = \mathbf{CJ}_j^{NIS} \cup \{j'\}$

      End

      If $\exists s \in \mathbf{S}^{ZW}: j \in \mathbf{J}_s^{P}, j' \in \mathbf{J}_s^{C}$

        $\mathbf{CJ}_j^{ZW} = \mathbf{CJ}_j^{ZW} \cup \{j'\}$

      End

  End

End

Determine $\mathbf{CJ0}_j$

For $j \in \mathbf{J}$

  $\mathbf{CJ0}_j = \mathbf{CJ}_j$

  For $j' \in \mathbf{CJ0}_j$

    For $s, s' \in \mathbf{S}^{in}: s' \neq s, j \in (\mathbf{J}_s^P \cap \mathbf{J}_{s'}^P), j' \in (\mathbf{J}_s^C \cap \mathbf{J}_{s'}^C)$

      If $\exists i \in (\mathbf{I}_j \cap \mathbf{I}_s^P \cap \mathbf{I}_{s'}^P)$

        $\mathbf{CJ0}_j = \mathbf{CJ0}_j \setminus \{j'\}$

      End

      If $\exists i' \in (\mathbf{I}_{j'} \cap \mathbf{I}_s^C \cap \mathbf{I}_{s'}^C)$

        $\mathbf{CJ0}_j = \mathbf{CJ0}_j \setminus \{j'\}$

      End

    End

  End

End

S6 Model M1

Objective functions

Maximization of profit

$$z = \sum_{s \in \mathbf{S}^P} \left[p_s \cdot \sum_j \sum_{i \in (\mathbf{I}_j \cap \mathbf{I}_s^P)} \sum_n \sum_{n - \Delta n \le n' \le n} (\rho_{si} \cdot b_{ijn'n}) \right] \tag{S6.1}$$

Minimization of makespan

$$T_{jn}^{\mathrm{f}} \le MS \qquad \forall j, n = N \tag{S6.2}$$

$$ST0_s + \sum_j \sum_{i \in (\mathbf{I}_j \cap \mathbf{I}_s^P)} \sum_n \sum\nolimits_{n \le n' \le n + \Delta n} (\rho_{si} \cdot b_{ijnn'}) \ge D_s \qquad \forall s \in \mathbf{S}^P \tag{S6.3}$$

Tightening constraints

$$\sum_{i \in (\mathbf{I}_j \cap \mathbf{I}_s^P)} \left(\sum_{n - \Delta n \le n' \le n} w_{ijn'n} + ys_{ijn} \right) \ge \sum_{i' \in (\mathbf{I}_{j'} \cap \mathbf{I}_s^C)} \sum_{n+1 \le n' \le n+1+\Delta n} w_{i'j'(n+1)n'} + zI_{jj'(n+1)} - 1$$

$$\forall s \in \mathbf{S}^{in}, j \in \mathbf{J}_s^P, j' \in \mathbf{CJ0}_j, j \ne j', j' \in \mathbf{J}_s^C, n < N \tag{S6.4}$$

$$\sum_{i' \in (\mathbf{I}_{j'} \cap \mathbf{I}_s^C)} \sum_{n+1 \le n' \le n+1+\Delta n} w_{i'j'(n+1)n'} \ge \sum_{i \in (\mathbf{I}_j \cap \mathbf{I}_s^P)} \left[\sum_{n - \Delta n \le n' \le n} w_{ijn'n} + ys_{ijn} \right] + zI_{jj'(n+1)} - 1$$

$$\forall s \in \mathbf{S}^{in}, j \in \mathbf{J}_s^P, j' \in \mathbf{CJ0}_j, j \ne j', j' \in \mathbf{J}_s^C, n < N \tag{S6.5}$$

$$zI_{jj'n} \ge zD_{jj'n} \qquad \forall j, j' \in \mathbf{CJ}_j^F, j \ne j', n > 1 \tag{S6.6}$$

$$2 \cdot zI_{jj'n} \le \sum_{i \in \mathbf{I}_j} \sum_{n-1-\Delta n \le n' \le n-1} w_{ijn'(n-1)} + \sum_{i \in (\mathbf{I}_j \cap \mathbf{I}^P)} ys_{ij(n-1)} + \sum_{i' \in \mathbf{I}_{j'}} \sum_{n \le n' \le n + \Delta n} w_{i'j'nn'}$$

$$\forall j, j' \in \mathbf{CJ}_j, j \ne j', \sum_{s \in (\mathbf{S}^R \cup \mathbf{S}^P), s:(j, j' \in \mathbf{J}_s)} 1 = 0, n > 1 \tag{S6.7}$$

Capacity constraints

$$B_{ij}^{\min} \cdot w_{ijnn'} \le b_{ijnn'} \le B_{ij}^{\max} \cdot w_{ijnn'} \qquad \forall j, i \in \mathbf{I}_j, n \le n' \le n + \Delta n \tag{S6.8}$$

Material balance constraints

$$ST_{sn}^{M1} = ST_{s(n-1)}^{M1} + \sum_j \sum_{i \in (\mathbf{I}_j \cap \mathbf{I}_s^P)} \sum_{n-1-\Delta n \le n' \le n-1} \rho_{si} \cdot b_{ijn'(n-1)}$$

$$+ \sum_j \sum_{i \in (\mathbf{I}_j \cap \mathbf{I}_s^C)} \sum_{n \le n' \le n + \Delta n} \rho_{si} \cdot b_{ijnn'} \qquad \forall s, n > 1 \tag{S6.9}$$

$$ST_{sn}^{M1} = ST0_s + \sum_j \sum_{i \in (\mathbf{I}_j \cap \mathbf{I}_s^P)} \rho_{si} \cdot bs0_{ij} + \sum_j \sum_{i \in (\mathbf{I}_j \cap \mathbf{I}_s^C)} \sum_{n \le n' \le n + \Delta n} \rho_{si} \cdot b_{ijnn'}$$

$$\forall s, n = 1 \tag{S6.10}$$

$$\sum_{j}\sum_{i\in(\mathbf{I}_j\cap\mathbf{I}_s^{\mathrm{P}})}\rho_{si}\cdot bs_{ijn}\leq ST_{sn}^{M1}\qquad\forall s\in\mathbf{S}^{FIS},n\tag{S6.11}$$

Duration constraints

$$T_{jn}^{\mathrm{f}}\geq T_{jn}^{\mathrm{s}}+\sum_{i\in\mathbf{I}_j}\sum_{n\leq n'\leq n+\Delta n}\left(\alpha_{ij}\cdot w_{ijnn'}+\beta_{ij}\cdot b_{ijnn'}\right)\qquad\forall j,n\tag{S6.12}$$

Sequence constraints for different tasks in the same unit

$$T_{j(n+1)}^{\mathrm{s}}\geq T_{jn}^{\mathrm{f}}\qquad\forall j,n<N\tag{S6.13}$$

$$T_{sjn}\leq T_{sj(n+1)}\qquad\forall s\in\mathbf{S}^{in},j\in\mathbf{J}_s^{P},n<N-1\tag{S6.14}$$

$$T_{sjn}\geq T_{jn}^{\mathrm{f}}-M\cdot\left[1-\sum_{i\in\left(\mathbf{I}_j\cap\mathbf{I}_s^{P}\right)}\sum_{n-\Delta n\leq n'\leq n}w_{ijn'n}\right]\qquad\forall s\in\mathbf{S}^{in},j\in\mathbf{J}_s^{P},n<N\tag{S6.15}$$

$$T_{sjn}\leq T_{j(n+1)}^{\mathrm{s}}+M\cdot\left[1-\sum_{i\in\left(\mathbf{I}_j\cap\mathbf{I}_s^{P}\right)}\left(\sum_{n-\Delta n\leq n'\leq n}w_{ijn'n}+ys_{ijn}\right)\right]$$

$$\forall s\in\mathbf{S}^{in},j\in\mathbf{J}_s^{P},n<N\tag{S6.16}$$

Material transfer

Indirect material transfer

$$T_{jn}^{\mathrm{f}}\leq T_{j'(n+1)}^{\mathrm{s}}+M\cdot\left[1-zI_{jj'(n+1)}\right]\qquad\forall j,j'\in\mathbf{CJ}_j,j\neq j',n<N\tag{S6.17}$$

$$\sum_{j'}\sum_{i'\in\left(\mathbf{I}_s^{C}\cap\mathbf{I}_{j'}\right)}\left(-\rho_{si'}\cdot\sum_{n\leq n'\leq n+\Delta n}b_{i'j'nn'}\right)\leq ST_{s(n-1)}^{M1}+\sum_{j}\sum_{j'}\sum_{i\in\left(\mathbf{I}_s^{P}\cap\mathbf{I}_j\right)}\sum_{i'\in\left(\mathbf{I}_s^{C}\cap\mathbf{I}_{j'}\right)}bTi_{iji'j'n}$$

$$\forall s\in\mathbf{S}^{in},n>1\tag{S6.19}$$

$$\sum_{j'}\sum_{i'\in\left(\mathbf{I}_s^{C}\cap\mathbf{I}_{j'}\right)}\left(-\rho_{si'}\cdot\sum_{n\leq n'\leq n+\Delta n}b_{i'j'nn'}\right)\leq ST0_s+\sum_{j}\sum_{j'}\sum_{i\in\left(\mathbf{I}_s^{P}\cap\mathbf{I}_j\right)}\sum_{i'\in\left(\mathbf{I}_s^{C}\cap\mathbf{I}_{j'}\right)}bTi_{iji'j'n}$$

$$\forall s\in\left(\mathbf{S}^{in}\cap\mathbf{S}^{B}\right),n=1\tag{S6.20}$$

$$\rho_{si}\cdot\sum_{n-1-\Delta n\leq n'\leq n-1}b_{ijn'(n-1)}\geq\sum_{j'}\sum_{i'\in\left(\mathbf{I}_{j'}\cap\mathbf{I}_s^{C}\right)}bTi_{iji'j'n}$$

$$\forall s\in\mathbf{S}^{in},j,i\in\left(\mathbf{I}_j\cap\mathbf{I}_s^{P}\right),n>1\tag{S6.21}$$

$$\rho_{si}\cdot bs0_{ij}\geq\sum_{j'}\sum_{i'\in\left(\mathbf{I}_{j'}\cap\mathbf{I}_s^{C}\right)}bTi_{iji'j'n}$$

$$\forall s\in\mathbf{S}^{in},j,i\in\left(\mathbf{I}_j\cap\mathbf{I}_s^{P}\right),n=1,bs0_{ij}>0\tag{S6.22}$$

$$-\rho_{si'} \cdot \sum_{n \le n' \le n+\Delta n} b_{i'j'nn'} \ge \sum_{j} \sum_{i \in (\mathbf{I}_j \cap \mathbf{I}_s^P)} bTi_{iji'j'n}$$

$$\forall s \in \mathbf{S}^{in}, j', i' \in (\mathbf{I}_{j'} \cap \mathbf{I}_s^C), n > 1 \qquad \text{(S6.23)}$$

$$-\rho_{si'} \cdot \sum_{n \le n' \le n+\Delta n} b_{i'j'nn'} \ge \sum_{j} \sum_{i \in (\mathbf{I}_j \cap \mathbf{I}_s^P)} bTi_{iji'j'n}$$

$$\forall s \in (\mathbf{S}^{in} \cap \mathbf{S}^B), j', i' \in (\mathbf{I}_{j'} \cap \mathbf{I}_s^C), n = 1 \qquad \text{(S6.24)}$$

$$\sum_{i \in \mathbf{I}_j} \sum_{i' \in \mathbf{I}_{j'}} bTi_{iji'j'n} \le \min\left[B_j^{\max}, B_{j'}^{\max}\right] \cdot zI_{jj'n} \qquad \forall j, j' \in \mathbf{CJ}_j, j \ne j', n > 1 \qquad \text{(S6.25)}$$

$$\sum_{i \in \mathbf{I}_j} \sum_{i' \in \mathbf{I}_{j'}} bTi_{iji'j'n} \le \min\left[B_j^{\max}, B_{j'}^{\max}\right] \cdot zI_{jj'n}$$

$$\forall j, j' \in \mathbf{CJ}_j, j \ne j', n = 1, \sum_{s \in \mathbf{S}^{FIS}} \sum_{i \in (\mathbf{I}_j \cap \mathbf{I}_s^P)} \sum_{i' \in (\mathbf{I}_{j'} \cap \mathbf{I}_s^C)} bs0_{ij} > 0 \qquad \text{(S6.26)}$$

where $B_j^{max} = \max_{s, i \in (\mathbf{I}_j \cap \mathbf{I}_s^P)} [\rho_{si} \cdot B_{ij}^{max}]$ and $B_{j'}^{max} = \max_{s, i' \in (\mathbf{I}_{j'} \cap \mathbf{I}_s^C)} [-\rho_{si'} \cdot B_{i'j'}^{max}]$.

$$T_{sjn} \le T_{j'(n+2)}^{\mathrm{s}} + M \cdot \left[1 - \sum_{i' \in (\mathbf{I}_{j'} \cap \mathbf{I}_s^C)} \sum_{n+2 \le n' \le n+2+\Delta n} w_{i'j'(n+2)n'}\right]$$

$$\forall s \in \mathbf{S}^{in}, j \ne j', j \in \mathbf{J}_s^P, j' \in \mathbf{J}_s^C, n < N-1 \qquad \text{(S6.27)}$$

Direct material transfer

$$T_{j'n}^{\mathrm{f}} \le T_{jn}^{\mathrm{f}} + M \cdot \left[1 - zD_{jj'(n+1)}\right] \qquad \forall j, j' \in \mathbf{CJ}_j^F, j \ne j', n < N \qquad \text{(S6.28)}$$

$$\sum_{j} \sum_{i \in (\mathbf{I}_j \cap \mathbf{I}_s^P)} \left[\rho_{si} \cdot \sum_{n-1-\Delta n \le n' \le n-1} b_{ijn'(n-1)}\right] + ST_{s(n-1)}^{M1}$$

$$\le ST_s^{\max} + \sum_{j} \sum_{j'} \sum_{i \in (\mathbf{I}_j \cap \mathbf{I}_s^P)} \sum_{i' \in (\mathbf{I}_{j'} \cap \mathbf{I}_s^C)} bTd_{iji'j'n}$$

$$+ \sum_{j} \sum_{i \in (\mathbf{I}_j \cap \mathbf{I}_s^P)} \rho_{si} \cdot bs_{ijn} \qquad \forall s \in \mathbf{S}^{FIS}, n > 1 \qquad \text{(S6.29)}$$

$$\sum_{j} \sum_{i \in (\mathbf{I}_j \cap \mathbf{I}_s^P)} \rho_{si} \cdot bs0_{ij} + ST0_s$$

$$\le ST_s^{\max} + \sum_{j} \sum_{j'} \sum_{i \in (\mathbf{I}_j \cap \mathbf{I}_s^P)} \sum_{i' \in (\mathbf{I}_{j'} \cap \mathbf{I}_s^C)} bTd_{iji'j'n} + \sum_{j} \sum_{i \in (\mathbf{I}_j \cap \mathbf{I}_s^P)} \rho_{si} \cdot bs_{ijn}$$

$$\forall s \in (\mathbf{S}^{FIS} \cap \mathbf{S}^B), n = 1 \qquad \text{(S6.30)}$$

Note that $ST0_s$ is the initial inventory of storage, and it does not include the amount of material

held in the processing units.

$$\rho_{si} \cdot \left[\sum_{n-1-\Delta n \le n' \le n-1} b_{ijn'(n-1)} + bs_{ij(n-1)} \right] \ge \sum_{j'} \sum_{i' \in \left(\mathbf{I}_s^C \cap \mathbf{I}_{j'}\right)} bTd_{iji'j'n} + \rho_{si} \cdot bs_{ijn}$$

$$\forall s \in \mathbf{S}^{FIS}, j, i \in \left(\mathbf{I}_j \cap \mathbf{I}_s^P\right), n > 1 \quad \text{(S6.31)}$$

$$\rho_{si} \cdot bs0_{ij} \ge \sum_{j'} \sum_{i' \in \left(\mathbf{I}_s^C \cap \mathbf{I}_{j'}\right)} bTd_{iji'j'n} + \rho_{si} \cdot bs_{ijn}$$

$$\forall s \in \mathbf{S}^{FIS}, j, i \in \left(\mathbf{I}_j \cap \mathbf{I}_s^P\right), n = 1, bs0_{ij} > 0 \quad \text{(S6.32)}$$

$$-\rho_{si'} \cdot \sum_{n \le n' \le n+\Delta n} b_{i'j'nn'} \ge \sum_{j} \sum_{i \in \left(\mathbf{I}_s^P \cap \mathbf{I}_j\right)} bTd_{iji'j'n}$$

$$\forall s \in \mathbf{S}^{FIS}, j', i' \in \left(\mathbf{I}_{j'} \cap \mathbf{I}_s^C\right), n > 1 \quad \text{(S6.33)}$$

$$-\rho_{si'} \cdot \sum_{n \le n' \le n+\Delta n} b_{i'j'nn'} \ge \sum_{j} \sum_{i \in \left(\mathbf{I}_s^P \cap \mathbf{I}_j\right)} bTd_{iji'j'n}$$

$$\forall s \in (\mathbf{S}^{FIS} \cap \mathbf{S}^B), j', i' \in \left(\mathbf{I}_{j'} \cap \mathbf{I}_s^C\right), n = 1 \quad \text{(S6.34)}$$

$$\sum_{i \in \mathbf{I}_j} \sum_{i' \in \mathbf{I}_{j'}} bTd_{iji'j'n} \le \min\left[B_j^{\max}, B_{j'}^{\max}\right] \cdot zD_{jj'n} \qquad \forall j, j' \in \mathbf{CJ}_j^F, j \ne j', n > 1 \quad \text{(S6.35)}$$

$$\sum_{i \in \mathbf{I}_j} \sum_{i' \in \mathbf{I}_{j'}} bTd_{iji'j'n} \le \min\left[B_j^{\max}, B_{j'}^{\max}\right] \cdot zD_{jj'n}$$

$$\forall j, j' \in \mathbf{CJ}_j', j \ne j', n = 1, \sum_{s \in \mathbf{S}^{FIS}} \sum_{i \in \left(\mathbf{I}_j \cap \mathbf{I}_s^P\right)} \sum_{i' \in \left(\mathbf{I}_{j'} \cap \mathbf{I}_s^C\right)} bs0_{ij} > 0 \quad \text{(S6.36)}$$

Sequence constraints for a state subject to FIS policy

$$T_{sjn} \ge T_{j'(n-1)}^{\mathrm{f}} - M \cdot \left[1 - \sum_{i' \in \left(\mathbf{I}_{j'} \cap \mathbf{I}_s^C\right)} \sum_{n \le n' \le n+\Delta n} w_{i'j'nn'} \right]$$

$$\forall s \in \mathbf{S}^{FIS}, j \ne j', j \in \mathbf{J}_s^P, j' \in \mathbf{J}_s^C, 1 < n < N \quad \text{(S6.37)}$$

Allowing processing units to store materials

$$bs_{ijn} \le B_{ij}^{\max} \cdot ys_{ijn} \qquad \forall j, i \in \left(\mathbf{I}_j \cap \mathbf{I}^P\right), n \quad \text{(S6.38)}$$

Allocation constraints

$$\sum_{i \in (\mathbf{I}_j \cap \mathbf{I}^P)} ys_{ijn} \le 1 - \sum_{i \in \mathbf{I}_j} \sum_{n-\Delta n \le n' \le n} \sum_{n \le n'' \le n'+\Delta n} w_{ijn'n''} \qquad \forall j, n \quad \text{(S6.39)}$$

Tightening constraint for minimization of makespan

$$\sum_{i \in \mathbf{I}_j} \sum_{n} \sum_{n \le n' \le n+\Delta n} (\alpha_{ij} \cdot w_{ijnn'} + \beta_{ij} \cdot b_{ijnn'}) \le MS \qquad \forall j \quad \text{(S6.40)}$$

Bounds and fixing

$$T_{jn}^{\mathrm{s}} \leq H, T_{jn}^{\mathrm{f}} \leq H \qquad \forall j,n \qquad \text{(S6.41,42)}$$

$$ST_{sn}^{M1} \leq ST_s^{max} + \sum_{j \in \mathbf{J}_s^P} \left[\max_{i \in (\mathbf{I}_j \cap \mathbf{I}_s^P)} \left(\rho_{si} \cdot B_{ij}^{\max} \right) \right] \qquad \forall s \in \mathbf{S}^{FIS}, n \qquad \text{(S6.43)}$$

$$bs_{ijn} \leq bs0_{ij} \qquad \forall j, i \in (\mathbf{I}_j \cap \mathbf{I}^P), n = 1 \qquad \text{(S6.44)}$$

$$ys_{ijn} = 0, bs_{ijn} = 0 \qquad \forall i \notin \mathbf{I}^P, j, n \qquad \text{(S6.45)}$$

$$w_{ijnn'} = 0, b_{ijnn'} = 0 \qquad \forall i, j, n, n' < n \qquad \text{(S6.46)}$$

$$w_{ijnn'} = 0, b_{ijnn'} = 0 \qquad \forall j, i \notin \mathbf{I}_j, n, n' \qquad \text{(S6.47)}$$

$$bTd_{iji'j'n} = bTi_{iji'j'n} = zI_{jj'n} = zD_{jj'n} = ys_{ijn} = bs_{ijn} = 0$$

$$\forall i, i', j, j', n = 1, bs0_{ij} = 0 \qquad \text{(S6.48)}$$

$$b_{ijnn'}, bs_{ijn}, bTi_{iji'j'n}, bTd_{iji'j'n}, MS, ST_{sn}^{M1}, T_{sjn}, T_{jn}^{\mathrm{s}}, T_{jn}^{\mathrm{f}} \geq 0 \qquad \text{(S6.49)}$$

$$w_{ijnn'}, ys_{ijn}, zD_{jj'n}, zI_{jj'n} \in \{0,1\} \qquad \text{(S6.50)}$$

The mathematical model **M1** consists of constraints (S6.1), (S6.4) - (S6.39) and (S6.41) - (S6.50) for maximization of profit, and (S6.2) - (S6.50) for minimization of makespan.

S7. Model M2

Objective functions

Maximizing profit

$$z = \sum_{s \in \mathbf{S}^P} \left[p_s \cdot \sum_j \sum_{i \in (\mathbf{I}_j \cap \mathbf{I}_s^P)} \sum_n \sum_{n-\Delta n \le n' \le n} \left(\rho_{si} \cdot b_{ijn'n} \right) \right] \quad \text{(S7.1)}$$

Minimizing makespan

$$T_{jn}^{\mathrm{f}} \le MS \qquad \forall j, n = N \quad \text{(S7.2)}$$

$$T_{sn}^{M2} \le MS \qquad \forall s \in \mathbf{S}^{in}, n = N \quad \text{(S7.3)}$$

$$ST0_s + \sum_j \sum_{i \in (\mathbf{I}_j \cap \mathbf{I}_s^P)} \sum_n \sum\nolimits_{n \le n' \le n+\Delta n} \left(\rho_{si} \cdot b_{ijnn'} \right) \ge D_s \qquad \forall s \in \mathbf{S}^P \quad \text{(S7.4)}$$

Tightening constraints

$$\sum_{i \in (\mathbf{I}_j \cap \mathbf{I}_s^P)} \left(\sum_{n-\Delta n \le n' \le n} w_{ijn'n} + ys_{ijn} \right) \ge \sum_{i' \in (\mathbf{I}_{j'} \cap \mathbf{I}_s^C)} \sum_{n+1 \le n' \le n+1+\Delta n} w_{i'j'(n+1)n'} + zI_{jj'(n+1)} - 1$$

$$\forall s \in \mathbf{S}^{in}, j \in \mathbf{J}_s^P, j' \in \mathbf{CJ0}_j, j \ne j', j' \in \mathbf{J}_s^C, n < N \quad \text{(S7.5)}$$

$$\sum_{i' \in (\mathbf{I}_{j'} \cap \mathbf{I}_s^C)} \sum_{n+1 \le n' \le n+1+\Delta n} w_{i'j'(n+1)n'} \ge \sum_{i \in (\mathbf{I}_j \cap \mathbf{I}_s^P)} \left[\sum_{n-\Delta n \le n' \le n} w_{ijn'n} + ys_{ijn} \right] + zI_{jj'(n+1)} - 1$$

$$\forall s \in \mathbf{S}^{in}, j \in \mathbf{J}_s^P, j' \in \mathbf{CJ0}_j, j \ne j', j' \in \mathbf{J}_s^C, n < N \quad \text{(S7.6)}$$

$$zI_{jj'n} \ge zD_{jj'n} \qquad \forall j, j' \in \mathbf{CJ}_j^F, j \ne j', n > 1 \quad \text{(S7.7)}$$

$$2 \cdot zI_{jj'n} \le \sum_{i \in \mathbf{I}_j} \sum_{n-1-\Delta n \le n' \le n-1} w_{ijn'(n-1)} + \sum_{i \in (\mathbf{I}_j \cap \mathbf{I}^P)} ys_{ij(n-1)} + \sum_{i' \in \mathbf{I}_{j'}} \sum_{n \le n' \le n+\Delta n} w_{i'j'nn'}$$

$$\forall j, j' \in \mathbf{CJ}_j, j \ne j', \sum_{s:(s \epsilon (\mathbf{S}^R \cup \mathbf{S}^P), j, j' \in \mathbf{J}_\mathrm{s})} 1 = 0, n > 1 \quad \text{(S7.8)}$$

Capacity constraints

$$B_{ij}^{\min} \cdot w_{ijnn'} \le b_{ijnn'} \le B_{ij}^{\max} \cdot w_{ijnn'} \qquad \forall j, i \in \mathbf{I}_j, n \le n' \le n + \Delta n \quad \text{(S7.9)}$$

Material balance constraints

$$ST_{sn}^{M2} = ST_{s(n-1)}^{M2} + \sum_j \sum_{i \in (\mathbf{I}_j \cap \mathbf{I}_s^P)} \sum_{n-1-\Delta n \le n' \le n-1} \rho_{si} \cdot b_{ijn'(n-1)} + \sum_j \sum_{i \in (\mathbf{I}_j \cap \mathbf{I}_s^P)} \rho_{si} \cdot bs_{ij(n-1)}$$

$$+ \sum_j \sum_{i \in (\mathbf{I}_j \cap \mathbf{I}_s^C)} \sum_{n \le n' \le n+\Delta n} \rho_{si} \cdot b_{ijnn'} - \sum_j \sum_{i \in (\mathbf{I}_j \cap \mathbf{I}_s^P)} \rho_{si} \cdot bs_{ijn}$$

$$\forall s, n > 1 \quad \text{(S7.10)}$$

$$ST_{sn}^{M2} = ST0_s + \sum_j \sum_{i \in (\mathbf{I}_j \cap \mathbf{I}_s^{\mathrm{P}})} \rho_{si} \cdot bs0_{ij} + \sum_j \sum_{i \in (\mathbf{I}_j \cap \mathbf{I}_s^{\mathrm{C}})} \sum_{n \le n' \le n+\Delta n} \rho_{si} \cdot b_{ijnn'} - \sum_j \sum_{i \in (\mathbf{I}_j \cap \mathbf{I}_s^{\mathrm{P}})} \rho_{si} \cdot bs_{ijn} \quad \forall s, n = 1 \tag{S7.11}$$

Duration constraints

$$T_{jn}^{\mathrm{f}} \ge T_{jn}^{\mathrm{s}} + \sum_{i \in \mathbf{I}_j} \sum_{n \le n' \le n+\Delta n} \left(\alpha_{ij} \cdot w_{ijnn'} + \beta_{ij} \cdot b_{ijnn'}\right) \quad \forall j, n \tag{S7.12}$$

Sequence constraints for different tasks in the same unit

$$T_{j(n+1)}^{\mathrm{s}} \ge T_{jn}^{\mathrm{f}} \quad \forall j, n < N \tag{S7.13}$$

$$T_{s(n+1)}^{M2} \ge T_{sn}^{M2} \quad \forall s \in \mathbf{S}^{in}, n < N \tag{S7.14}$$

$$T_{sn}^{M2} \ge T_{jn}^{\mathrm{f}} - M \cdot \left[1 - \sum_{i \in (\mathbf{I}_j \cap \mathbf{I}_s^P)} \sum_{n-\Delta n \le n' \le n} w_{ijn'n}\right] \quad \forall s \in \mathbf{S}^{in}, j \in \mathbf{J}_s^P, n \tag{S7.15}$$

Material transfer

Indirect material transfer

$$T_{jn}^{\mathrm{f}} \le T_{j'(n+1)}^{\mathrm{s}} + M \cdot \left[1 - zI_{jj'(n+1)}\right] \quad \forall j, j' \in \mathbf{CJ}_j, j \ne j', n < N \tag{S7.16}$$

$$\sum_{j'} \sum_{i' \in \left(\mathbf{I}_s^C \cap \mathbf{I}_{j'}\right)} \left(-\rho_{si'} \cdot \sum_{n \le n' \le n+\Delta n} b_{i'j'nn'}\right) \le ST_{s(n-1)}^{M2} + \sum_j \sum_{j'} \sum_{i \in (\mathbf{I}_s^P \cap \mathbf{I}_j)} \sum_{i' \in \left(\mathbf{I}_s^C \cap \mathbf{I}_{j'}\right)} bTi_{iji'j'n} \quad \forall s \in \mathbf{S}^{in}, n > 1 \tag{S7.17}$$

$$\sum_{j'} \sum_{i' \in \left(\mathbf{I}_s^C \cap \mathbf{I}_{j'}\right)} \left(-\rho_{si'} \cdot \sum_{n \le n' \le n+\Delta n} b_{i'j'nn'}\right) \le ST0_s + \sum_j \sum_{j'} \sum_{i \in (\mathbf{I}_s^P \cap \mathbf{I}_j)} \sum_{i' \in \left(\mathbf{I}_s^C \cap \mathbf{I}_{j'}\right)} bTi_{iji'j'n} \quad \forall s \in (\mathbf{S}^{in} \cap \mathbf{S}^B), n = 1 \tag{S7.18}$$

$$\rho_{si} \cdot \left[\sum_{n-1-\Delta n \le n' \le n-1} b_{ijn'(n-1)} + bs_{ij(n-1)}\right] \ge \sum_{j'} \sum_{i' \in \left(\mathbf{I}_{j'} \cap \mathbf{I}_s^C\right)} bTi_{iji'j'n} + \rho_{si} \cdot bs_{ijn} \quad \forall s \in \mathbf{S}^{in}, j, i \in \left(\mathbf{I}_j \cap \mathbf{I}_s^P\right), n > 1 \tag{S7.19}$$

$$\rho_{si} \cdot bs0_{ij} \ge \sum_{j'} \sum_{i' \in \left(\mathbf{I}_{j'} \cap \mathbf{I}_s^C\right)} bTi_{iji'j'n} + \rho_{si} \cdot bs_{ijn} \quad \forall s \in \mathbf{S}^{in}, j, i \in \left(\mathbf{I}_j \cap \mathbf{I}_s^P\right), n = 1, bs0_{ij} > 0 \tag{S7.20}$$

$$-\rho_{si'} \cdot \sum_{n \le n' \le n+\Delta n} b_{i'j'nn'} \ge \sum_j \sum_{i \in (\mathbf{I}_j \cap \mathbf{I}_s^P)} bTi_{iji'j'n}$$

$$\forall s \in \mathbf{S}^{in}, j', i' \in \left(\mathbf{I}_{j'} \cap \mathbf{I}_s^C\right), n > 1 \qquad \text{(S7.21)}$$

$$-\rho_{si'} \cdot \sum_{n \le n' \le n+\Delta n} b_{i'j'nn'} \ge \sum_{j} \sum_{i \in \left(\mathbf{I}_j \cap \mathbf{I}_s^P\right)} bTi_{iji'j'n}$$

$$\forall s \in (\mathbf{S}^{in} \cap \mathbf{S}^B), j', i' \in \left(\mathbf{I}_{j'} \cap \mathbf{I}_s^C\right), n = 1 \qquad \text{(S7.22)}$$

$$\sum_{i \in \mathbf{I}_j} \sum_{i' \in \mathbf{I}_{j'}} bTi_{iji'j'n} \le \min\left[B_j^{\max}, B_{j'}^{\max}\right] \cdot zI_{jj'n} \qquad \forall j, j' \in \mathbf{CJ}_j, j \ne j', n > 1 \qquad \text{(S7.23)}$$

$$\sum_{i \in \mathbf{I}_j} \sum_{i' \in \mathbf{I}_{j'}} bTi_{iji'j'n} \le \min\left[B_j^{\max}, B_{j'}^{\max}\right] \cdot zI_{jj'n}$$

$$\forall j, j' \in \mathbf{CJ}_j, j \ne j', n = 1, \sum\nolimits_{s \in \mathbf{S}^{FIS}} \sum\nolimits_{i \in \left(\mathbf{I}_j \cap \mathbf{I}_s^P\right)} \sum\nolimits_{i' \in \left(\mathbf{I}_{j'} \cap \mathbf{I}_s^C\right)} bs0_{ij} > 0 \qquad \text{(S7.24)}$$

$$T_{sn}^{M2} \le T_{j'(n+2)}^{s} + M \cdot \left[1 - \sum_{i' \in \left(\mathbf{I}_{j'} \cap \mathbf{I}_s^C\right)} \sum_{n+2 \le n' \le n+2+\Delta n} w_{i'j'(n+2)n'}\right]$$

$$\forall s \in \mathbf{S}^{in}, j' \in \mathbf{J}_s^C, n < N - 1 \qquad \text{(S7.25)}$$

Direct material transfer

$$T_{j'n}^{\mathrm{f}} \le T_{jn}^{\mathrm{f}} + M \cdot \left[1 - zD_{jj'(n+1)}\right] \qquad \forall j, j' \in \mathbf{CJ}_j^F, j \ne j', n < N \qquad \text{(S7.26)}$$

$$\sum_{j} \sum_{i \in \left(\mathbf{I}_j \cap \mathbf{I}_s^P\right)} \left[\rho_{si} \cdot \sum_{n-1-\Delta n \le n' \le n-1} b_{ijn'(n-1)}\right] + \sum_{j} \sum_{i \in \left(\mathbf{I}_j \cap \mathbf{I}_s^P\right)} \rho_{si} \cdot bs_{ij(n-1)} + ST_{s(n-1)}^{M2}$$
$$\le ST_s^{\max} + \sum_{j} \sum_{j'} \sum_{i \in \left(\mathbf{I}_j \cap \mathbf{I}_s^P\right)} \sum_{i' \in \left(\mathbf{I}_{j'} \cap \mathbf{I}_s^C\right)} bTd_{iji'j'n} + \sum_{j} \sum_{i \in \left(\mathbf{I}_j \cap \mathbf{I}_s^P\right)} \rho_{si} \cdot bs_{ijn}$$

$$\forall s \in \mathbf{S}^{FIS}, n > 1 \qquad \text{(S7.27)}$$

$$\sum_{j} \sum_{i \in \left(\mathbf{I}_j \cap \mathbf{I}_s^P\right)} \rho_{si} \cdot bs0_{ij} + ST0_s$$
$$\le ST_s^{\max} + \sum_{j} \sum_{j'} \sum_{i \in \left(\mathbf{I}_j \cap \mathbf{I}_s^P\right)} \sum_{i' \in \left(\mathbf{I}_{j'} \cap \mathbf{I}_s^C\right)} bTd_{iji'j'n} + \sum_{j} \sum_{i \in \left(\mathbf{I}_j \cap \mathbf{I}_s^P\right)} \rho_{si} \cdot bs_{ijn}$$

$$\forall s \in (\mathbf{S}^{FIS} \cap \mathbf{S}^B), n = 1 \qquad \text{(S7.28)}$$

$$\rho_{si} \cdot \left[\sum_{n-1-\Delta n \le n' \le n-1} b_{ijn'(n-1)} + bs_{ij(n-1)}\right] \ge \sum_{j'} \sum_{i' \in \left(\mathbf{I}_s^C \cap \mathbf{I}_{j'}\right)} bTd_{iji'j'n} + \rho_{si} \cdot bs_{ijn}$$

$$\forall s \in \mathbf{S}^{FIS}, j, i \in \left(\mathbf{I}_j \cap \mathbf{I}_s^P\right), n > 1 \qquad \text{(S7.29)}$$

$$\rho_{si} \cdot bs0_{ij} \ge \sum_{j'} \sum_{i' \in \left(\mathbf{I}_s^C \cap \mathbf{I}_{j'}\right)} bTd_{iji'j'n} + \rho_{si} \cdot bs_{ijn}$$

$$\forall s \in \mathbf{S}^{FIS}, j, i \in \left(\mathbf{I}_j \cap \mathbf{I}_s^P\right), n = 1, bs0_{ij} > 0 \quad \text{(S7.30)}$$

$$-\rho_{si'} \cdot \sum_{n \le n' \le n+\Delta n} b_{i'j'nn'} \ge \sum_{j} \sum_{i \in \left(\mathbf{I}_s^P \cap \mathbf{I}_j\right)} bTd_{iji'j'n}$$

$$\forall s \in \mathbf{S}^{FIS}, j', i' \in \left(\mathbf{I}_{j'} \cap \mathbf{I}_s^C\right), n > 1 \quad \text{(S7.31)}$$

$$-\rho_{si'} \cdot \sum_{n \le n' \le n+\Delta n} b_{i'j'nn'} \ge \sum_{j} \sum_{i \in \left(\mathbf{I}_s^P \cap \mathbf{I}_j\right)} bTd_{iji'j'n}$$

$$\forall s \in (\mathbf{S}^{FIS} \cap \mathbf{S}^B), j', i' \in \left(\mathbf{I}_{j'} \cap \mathbf{I}_s^C\right), n = 1 \quad \text{(S7.32)}$$

$$\sum_{i \in \mathbf{I}_j} \sum_{i' \in \mathbf{I}_{j'}} bTd_{iji'j'n} \le \min\left[B_j^{\max}, B_{j'}^{\max}\right] \cdot zD_{jj'n} \quad \forall j, j' \in \mathbf{CJ}_j^F, j \ne j', n > 1 \quad \text{(S7.33)}$$

$$\sum_{i \in \mathbf{I}_j} \sum_{i' \in \mathbf{I}_{j'}} bTd_{iji'j'n} \le \min\left[B_j^{\max}, B_{j'}^{\max}\right] \cdot zD_{jj'n}$$

$$\forall j, j' \in \mathbf{CJ}_j^F, j \ne j', n = 1, \sum\nolimits_{s \in \mathbf{S}^{FIS}} \sum\nolimits_{i \in \left(\mathbf{I}_j \cap \mathbf{I}_s^P\right)} \sum\nolimits_{i' \in \left(\mathbf{I}_{j'} \cap \mathbf{I}_s^C\right)} bs0_{ij} > 0 \quad \text{(S7.34)}$$

Sequence constraints for a state subject to FIS policy

$$tt_{sn}^{M2} \le T_{j(n+1)}^{\mathrm{s}} + M \cdot \left[1 - \sum_{i \in \left(\mathbf{I}_j \cap \mathbf{I}_s^P\right)} \left(\sum_{n-\Delta n \le n' \le n} w_{ijn'n} + ys_{ijn} \right)\right]$$

$$\forall s \in \mathbf{S}^{FIS}, j \in \mathbf{J}_s^P, n < N \quad \text{(S7.35)}$$

$$tt_{sn}^{M2} \ge T_{j'(n-1)}^{\mathrm{f}} - M \cdot \left[1 - \sum_{i' \in \left(\mathbf{I}_{j'} \cap \mathbf{I}_s^C\right)} \sum_{n \le n' \le n+\Delta n} w_{i'j'nn'}\right]$$

$$\forall s \in \mathbf{S}^{FIS}, j' \in \mathbf{J}_s^C, n > 1, n < N \quad \text{(S7.36)}$$

$$tt_{s(n+1)}^{M2} \ge tt_{sn}^{M2} \quad \forall s \in \mathbf{S}^{FIS}, n < N-1 \quad \text{(S7.37)}$$

Allowing processing units to store materials

$$bs_{ijn} \le B_{ij}^{\max} \cdot ys_{ijn} \quad \forall j, i \in \left(\mathbf{I}_j \cap \mathbf{I}^P\right), n \quad \text{(S7.38)}$$

Allocation constraints

$$\sum_{i \in (\mathbf{I}_j \cap \mathbf{I}^P)} ys_{ijn} \le 1 - \sum_{i \in \mathbf{I}_j} \sum_{n-\Delta n \le n' \le n} \sum_{n \le n'' \le n'+\Delta n} w_{ijn'n''} \quad \forall j, n \quad \text{(S7.39)}$$

Tightening constraints for Minimization of makespan

$$\sum_{i \in \mathbf{I}_j} \sum_{n} \sum_{n \le n' \le n+\Delta n} (\alpha_{ij} \cdot w_{ijnn'} + \beta_{ij} \cdot b_{ijnn'}) \le MS \quad \forall j \quad \text{(S7.40)}$$

Bounds and fixing

$$T_{jn}^{\mathrm{s}} \le H, T_{jn}^{\mathrm{f}} \le H \quad \forall j, n \quad \text{(S7.41)}$$

$$ST_{sn}^{M2} \leq ST_s^{max} \qquad \forall s \in \mathbf{S}^{FIS}, n \in N \qquad (S7.42)$$

$$b_{ijnn'} \leq B_{ij}^{max} \qquad \forall j, i \in \mathbf{I}_j, n, n \leq n' \leq n + \Delta n \qquad (S7.43)$$

$$bs_{ijn} \leq bs0_{ij} \qquad \forall j, i \in \left(\mathbf{I}_j \cap \mathbf{I}^P\right), n = 1 \qquad (S7.44)$$

$$bs_{ijn} \leq B_{ij}^{max} \qquad \forall j, i \in \mathbf{I}_j, n \qquad (S7.45)$$

$$ys_{ijn} = 0, bs_{ijn} = 0 \qquad \forall i \notin \mathbf{I}^P, j, n \qquad (S7.46)$$

$$w_{ijnn'} = 0, b_{ijnn'} = 0 \qquad \forall i, j, n, n' < n \qquad (S7.47)$$

$$w_{ijnn'} = 0, b_{ijnn'} = 0 \qquad \forall j, i \notin \mathbf{I}_j, n, n' \qquad (S7.48)$$

$$bTd_{iji'j'n} \leq \min\left[\left(\rho_{si} \cdot B_{ij}^{max}\right), \left(-\rho_{si'} \cdot B_{i'j'}^{max}\right)\right]$$

$$\forall s \in \mathbf{S}^{FIS}, j, i \in \left(\mathbf{I}_j \cap \mathbf{I}_s^P\right), j', i' \in \left(\mathbf{I}_{j'} \cap \mathbf{I}_s^C\right), n \qquad (S7.49)$$

$$bTi_{iji'j'n} \leq \min\left[\left(\rho_{si} \cdot B_{ij}^{max}\right), \left(-\rho_{si'} \cdot B_{i'j'}^{max}\right)\right]$$

$$\forall s \in \mathbf{S}^{in}, j, i \in \left(\mathbf{I}_j \cap \mathbf{I}_s^P\right), j', i' \in \left(\mathbf{I}_{j'} \cap \mathbf{I}_s^C\right), n \qquad (S7.50)$$

$$bTd_{iji'j'n} = 0, bTi_{iji'j'n} = 0, zI_{jj'n} = 0, zD_{jj'n} = 0 \qquad \forall i, i', j, j', n = 1, bs0_{ij} = 0 \qquad (S7.51)$$

$$b_{ijnn'}, bs_{ijn}, bTi_{iji'j'n}, bTd_{iji'j'n}, MS, ST_{sn}^{M2}, tt_{sn}^{M2}, T_{sn}^{M2}, T_{jn}^{s}, T_{jn}^{f} \geq 0 \qquad (S7.52)$$

$$w_{ijnn'}, ys_{ijn}, zD_{jj'n}, zI_{jj'n} \in \{0,1\} \qquad (S7.53)$$

The mathematical model **M2** consists of constraints (S7.1), (S7.5) - (S7.39) and (S7.41) - (S7.53) for maximization of profit, and (S7.2) - (S7.53) for minimization of makespan.

S8. Model M3

This model is almost the same as model **M2** but uses T_{sjn}. It is used to compare with model **M2** justifying why T_{sjn} is not used in model **M2**.

Objective functions

$$z = \sum_{s \in \mathbf{S}^P} \left[p_s \cdot \sum_j \sum_{i \in (\mathbf{I}_j \cap \mathbf{I}_s^P)} \sum_n \sum_{n - \Delta n \le n' \le n} (\rho_{si} \cdot b_{ijn'n}) \right] \quad \text{(S8.1)}$$

$$T_{jn}^{\mathrm{f}} \le MS \qquad \forall j, n = N \quad \text{(S8.2)}$$

$$ST0_s + \sum_j \sum_{i \in (\mathbf{I}_j \cap \mathbf{I}_s^P)} \sum_n \sum_{n \le n' \le n + \Delta n} (\rho_{si} \cdot b_{ijnn'}) \ge D_s \qquad \forall s \in \mathbf{S}^P \quad \text{(S8.3)}$$

Tightening constraints

$$\sum_{i \in (\mathbf{I}_j \cap \mathbf{I}_s^P)} \left(\sum_{n - \Delta n \le n' \le n} w_{ijn'n} + ys_{ijn} \right) \ge \sum_{i' \in (\mathbf{I}_{j'} \cap \mathbf{I}_s^C)} \sum_{n+1 \le n' \le n+1+\Delta n} w_{i'j'(n+1)n'} + zI_{jj'(n+1)} - 1$$

$$\forall s \in \mathbf{S}^{in}, j \in \mathbf{J}_s^P, j' \in \mathbf{CJ0}_j, j \ne j', j' \in \mathbf{J}_s^C, n < N \quad \text{(S8.4)}$$

$$\sum_{i' \in (\mathbf{I}_{j'} \cap \mathbf{I}_s^C)} \sum_{n+1 \le n' \le n+1+\Delta n} w_{i'j'(n+1)n'} \ge \sum_{i \in (\mathbf{I}_j \cap \mathbf{I}_s^P)} \left[\sum_{n - \Delta n \le n' \le n} w_{ijn'n} + ys_{ijn} \right] + zI_{jj'(n+1)} - 1$$

$$\forall s \in \mathbf{S}^{in}, j \in \mathbf{J}_s^P, j' \in \mathbf{CJ0}_j, j \ne j', j' \in \mathbf{J}_s^C, n < N \quad \text{(S8.5)}$$

$$zI_{jj'n} \ge zD_{jj'n} \qquad \forall j, j' \in \mathbf{CJ}_j^F, j \ne j', n > 1 \quad \text{(S8.6)}$$

$$2 \cdot zI_{jj'n} \le \sum_{i \in \mathbf{I}_j} \sum_{n-1-\Delta n \le n' \le n-1} w_{ijn'(n-1)} + \sum_{i \in (\mathbf{I}_j \cap \mathbf{I}^P)} ys_{ij(n-1)} + \sum_{i' \in \mathbf{I}_{j'}} \sum_{n \le n' \le n + \Delta n} w_{i'j'nn'}$$

$$\forall j, j' \in \mathbf{CJ}_j, j \ne j', \sum_{s \in (\mathbf{S}^R \cup \mathbf{S}^P), s:(j,j' \in \mathbf{J}_s)} 1 = 0, n > 1 \quad \text{(S8.7)}$$

Capacity constraints

$$B_{ij}^{\min} \cdot w_{ijnn'} \le b_{ijnn'} \le B_{ij}^{\max} \cdot w_{ijnn'} \qquad \forall j, i \in \mathbf{I}_j, n \le n' \le n + \Delta n \quad \text{(S8.8)}$$

Material balance constraints

$$ST_{sn}^{M2} = ST_{s(n-1)}^{M2} + \sum_{j} \sum_{i \in (\mathbf{I}_j \cap \mathbf{I}_s^P)} \sum_{n-1-\Delta n \le n' \le n-1} \rho_{si} \cdot b_{ijn'(n-1)} + \sum_{j} \sum_{i \in (\mathbf{I}_j \cap \mathbf{I}_s^P)} \rho_{si} \cdot bs_{ij(n-1)}$$

$$+ \sum_{j} \sum_{i \in (\mathbf{I}_j \cap \mathbf{I}_s^C)} \sum_{n \le n' \le n+\Delta n} \rho_{si} \cdot b_{ijnn'} - \sum_{j} \sum_{i \in (\mathbf{I}_j \cap \mathbf{I}_s^P)} \rho_{si} \cdot bs_{ijn}$$

$$\forall s, n > 1 \quad \text{(S8.9)}$$

$$ST_{sn}^{M2} = ST0_s + \sum_{j} \sum_{i \in (\mathbf{I}_j \cap \mathbf{I}_s^{\mathrm{P}})} \rho_{si} \cdot bs0_{ij}$$

$$+ \sum_{j} \sum_{i \in (\mathbf{I}_j \cap \mathbf{I}_s^{\mathrm{C}})} \sum_{n \le n' \le n+\Delta n} \rho_{si} \cdot b_{ijnn'} - \sum_{j} \sum_{i \in (\mathbf{I}_j \cap \mathbf{I}_s^{\mathrm{P}})} \rho_{si} \cdot bs_{ijn} \quad \forall s, n = 1 \quad \text{(S8.10)}$$

Duration constraints

$$T_{jn}^{\mathrm{f}} \ge T_{jn}^{\mathrm{s}} + \sum_{i \in \mathbf{I}_j} \sum_{n \le n' \le n+\Delta n} \left(\alpha_{ij} \cdot w_{ijnn'} + \beta_{ij} \cdot b_{ijnn'}\right) \quad \forall j, n \quad \text{(S8.11)}$$

$$T_{jn}^{\mathrm{f}} \le T_{jn}^{\mathrm{s}} + M \cdot \left(\sum_{i \in \mathbf{I}_j} \sum_{n \le n' \le n+\Delta n} w_{ijnn'} + \sum_{i \in (\mathbf{I}_j \cap \mathbf{I}^P)} ys_{ijn} \right) \quad \forall j, n \quad \text{(S8.12)}$$

Sequencing constraints for different tasks in the same unit

$$T_{j(n+1)}^{\mathrm{s}} \ge T_{jn}^{\mathrm{f}} \quad \forall j, n < N \quad \text{(S8.13)}$$

$$T_{sjn} \le T_{sj(n+1)} \quad \forall s \in \mathbf{S}^{in}, j \in \mathbf{J}_s^P, n < N-1 \quad \text{(S8.14)}$$

$$T_{sjn} \ge T_{jn}^{\mathrm{f}} - M \cdot \left[1 - \sum_{i \in (\mathbf{I}_j \cap \mathbf{I}_s^P)} \left(\sum_{n-\Delta n \le n' \le n} w_{ijn'n} + ys_{ijn} \right) \right]$$

$$\forall s \in \mathbf{S}^{in}, j \in \mathbf{J}_s^P, n < N \quad \text{(S8.15)}$$

$$T_{sjn} \le T_{j(n+1)}^{\mathrm{s}} + M \left[1 - \sum_{i \in (\mathbf{I}_j \cap \mathbf{I}_s^P)} \left(\sum_{n-\Delta n \le n' \le n} w_{ijn'n} + ys_{ijn} \right) \right]$$

$$\forall s \in \mathbf{S}^{in}, j \in \mathbf{J}_s^P, n < N \quad \text{(S8.16)}$$

Material transfer

Indirect material transfer

$$T_{jn}^{\mathrm{f}} \leq T_{j'(n+1)}^{\mathrm{s}} + M \cdot \left[1 - zI_{jj'(n+1)}\right] \qquad \forall j, j' \in \mathbf{CJ}_j, j \neq j', n < N \qquad \text{(S8.17)}$$

$$\sum_{j'} \sum_{i' \in \left(\mathbf{I}_s^C \cap \mathbf{I}_{j'}\right)} \left(-\rho_{si'} \cdot \sum_{n \leq n' \leq n+\Delta n} b_{i'j'nn'}\right) \leq ST_{s(n-1)}^{M2} + \sum_j \sum_{j'} \sum_{i \in \left(\mathbf{I}_s^P \cap \mathbf{I}_j\right)} \sum_{i' \in \left(\mathbf{I}_s^C \cap \mathbf{I}_{j'}\right)} bTi_{iji'j'n}$$

$$\forall s \in \mathbf{S}^{in}, n > 1 \qquad \text{(S8.18)}$$

$$\sum_{j'} \sum_{i' \in \left(\mathbf{I}_s^C \cap \mathbf{I}_{j'}\right)} \left(-\rho_{si'} \cdot \sum_{n \leq n' \leq n+\Delta n} b_{i'j'nn'}\right) \leq ST0_s + \sum_j \sum_{j'} \sum_{i \in \left(\mathbf{I}_s^P \cap \mathbf{I}_j\right)} \sum_{i' \in \left(\mathbf{I}_s^C \cap \mathbf{I}_{j'}\right)} bTi_{iji'j'n}$$

$$\forall s \in (\mathbf{S}^{in} \cap \mathbf{S}^B), n = 1 \qquad \text{(S8.19)}$$

$$\rho_{si} \cdot \left[\sum_{n-1-\Delta n \leq n' \leq n-1} b_{ijn'(n-1)} + bs_{ij(n-1)}\right] \geq \sum_{j'} \sum_{i' \in \left(\mathbf{I}_{j'} \cap \mathbf{I}_s^C\right)} bTi_{iji'j'n} + \rho_{si} \cdot bs_{ijn}$$

$$\forall s \in \mathbf{S}^{in}, j, i \in \left(\mathbf{I}_j \cap \mathbf{I}_s^P\right), n > 1 \qquad \text{(S8.20)}$$

$$\rho_{si} \cdot bs0_{ij} \geq \sum_{j'} \sum_{i' \in \left(\mathbf{I}_{j'} \cap \mathbf{I}_s^C\right)} bTi_{iji'j'n} + \rho_{si} \cdot bs_{ijn}$$

$$\forall s \in \mathbf{S}^{in}, j, i \in \left(\mathbf{I}_j \cap \mathbf{I}_s^P\right), n = 1, bs0_{ij} > 0 \qquad \text{(S8.21)}$$

$$-\rho_{si'} \cdot \sum_{n \leq n' \leq n+\Delta n} b_{i'j'nn'} \geq \sum_j \sum_{i \in \left(\mathbf{I}_j \cap \mathbf{I}_s^P\right)} bTi_{iji'j'n}$$

$$\forall s \in \mathbf{S}^{in}, j', i' \in \left(\mathbf{I}_{j'} \cap \mathbf{I}_s^C\right), n > 1 \qquad \text{(S8.22)}$$

$$-\rho_{si'} \cdot \sum_{n \leq n' \leq n+\Delta n} b_{i'j'nn'} \geq \sum_j \sum_{i \in \left(\mathbf{I}_j \cap \mathbf{I}_s^P\right)} bTi_{iji'j'n}$$

$$\forall s \in (\mathbf{S}^{in} \cap \mathbf{S}^B), j', i' \in \left(\mathbf{I}_{j'} \cap \mathbf{I}_s^C\right), n = 1 \qquad \text{(S8.23)}$$

$$\sum_{i \in \mathbf{I}_j} \sum_{i' \in \mathbf{I}_{j'}} bTi_{iji'j'n} \leq \min\left[B_j^{\max}, B_{j'}^{\max}\right] \cdot zI_{jj'n} \qquad \forall j, j' \in \mathbf{CJ}_j, j \neq j', n > 1 \qquad \text{(S8.24)}$$

$$\sum_{i \in \mathbf{I}_j} \sum_{i' \in \mathbf{I}_{j'}} bTi_{iji'j'n} \leq \min\left[B_j^{\max}, B_{j'}^{\max}\right] \cdot zI_{jj'n}$$

$$\forall j, j' \in \mathbf{CJ}_j, j \neq j', n = 1, \sum_{s \in \mathbf{S}^{FIS}} \sum_{i \in \left(\mathbf{I}_j \cap \mathbf{I}_s^P\right)} \sum_{i' \in \left(\mathbf{I}_{j'} \cap \mathbf{I}_s^C\right)} bs0_{ij} > 0 \quad \text{(S8.25)}$$

$$T_{sjn} \leq T_{j'(n+2)}^{\mathrm{s}} + M \cdot \left[1 - \sum_{i' \in \left(\mathbf{I}_{j'} \cap \mathbf{I}_s^C\right)} \sum_{n+2 \leq n' \leq n+2+\Delta n} w_{i'j'(n+2)n'}\right]$$

$$\forall s \in \mathbf{S}^{in}, j \neq j', j \in \mathbf{J}_s^P, j' \in \mathbf{J}_s^C, n < N - 1 \quad \text{(S8.26)}$$

Direct material transfer

$$T_{j'n}^{\mathrm{f}} \leq T_{jn}^{\mathrm{f}} + M \cdot \left[1 - zD_{jj'(n+1)}\right] \qquad \forall j, j' \in \mathbf{CJ}_j^F, j \neq j', n < N \quad \text{(S8.27)}$$

$$\sum_j \sum_{i \in \left(\mathbf{I}_j \cap \mathbf{I}_s^P\right)} \left[\rho_{si} \cdot \sum_{n-1-\Delta n \leq n' \leq n-1} b_{ijn'(n-1)}\right] + \sum_j \sum_{i \in \left(\mathbf{I}_j \cap \mathbf{I}_s^P\right)} \rho_{si} \cdot bs_{ij(n-1)} + ST_{s(n-1)}^{M2}$$

$$\leq ST_s^{\max} + \sum_j \sum_{j'} \sum_{i \in \left(\mathbf{I}_j \cap \mathbf{I}_s^P\right)} \sum_{i' \in \left(\mathbf{I}_{j'} \cap \mathbf{I}_s^C\right)} bTd_{iji'j'n} + \sum_j \sum_{i \in \left(\mathbf{I}_j \cap \mathbf{I}_s^P\right)} \rho_{si} \cdot bs_{ijn}$$

$$\forall s \in \mathbf{S}^{FIS}, n > 1 \quad \text{(S8.28)}$$

$$\sum_j \sum_{i \in \left(\mathbf{I}_j \cap \mathbf{I}_s^P\right)} \rho_{sij} \cdot bs0_{ij} + ST0_s$$

$$\leq ST_s^{\max} + \sum_j \sum_{j'} \sum_{i \in \left(\mathbf{I}_j \cap \mathbf{I}_s^P\right)} \sum_{i' \in \left(\mathbf{I}_{j'} \cap \mathbf{I}_s^C\right)} bTd_{iji'j'n} + \sum_j \sum_{i \in \left(\mathbf{I}_j \cap \mathbf{I}_s^P\right)} \rho_{sij} \cdot bs_{ijn}$$

$$\forall s \in \left(\mathbf{S}^{FIS} \cap \mathbf{S}^B\right), n = 1 \quad \text{(S8.29)}$$

$$\rho_{si} \cdot \left[\sum_{n-1-\Delta n \leq n' \leq n-1} b_{ijn'(n-1)} + bs_{ij(n-1)}\right] \geq \sum_{j'} \sum_{i' \in \left(\mathbf{I}_{j'} \cap \mathbf{I}_s^C\right)} bTd_{iji'j'n} + \rho_{si} \cdot bs_{ijn}$$

$$\forall s \in \mathbf{S}^{FIS}, j, i \in \left(\mathbf{I}_j \cap \mathbf{I}_s^P\right), n > 1 \quad \text{(S8.30)}$$

$$\rho_{si} \cdot bs0_{ij} \geq \sum_{j'} \sum_{i' \in \left(\mathbf{I}_s^C \cap \mathbf{I}_{j'}\right)} bTd_{iji'j'n} + \rho_{si} \cdot bs_{ijn}$$

$$\forall s \in \mathbf{S}^{FIS}, j, i \in \left(\mathbf{I}_j \cap \mathbf{I}_s^P\right), n = 1, bs0_{ij} > 0 \quad \text{(S8.31)}$$

$$-\rho_{si'} \cdot \sum_{n \le n' \le n+\Delta n} b_{i'j'nn'} \ge \sum_{j} \sum_{i \in \left(\mathbf{I}_s^P \cap \mathbf{I}_j\right)} bTd_{iji'j'n}$$

$$\forall s \in \mathbf{S}^{FIS}, j', i' \in \left(\mathbf{I}_{j'} \cap \mathbf{I}_s^C\right), n > 1 \quad \text{(S8.32)}$$

$$-\rho_{si'} \cdot \sum_{n \le n' \le n+\Delta n} b_{i'j'nn'} \ge \sum_{j} \sum_{i \in \left(\mathbf{I}_s^P \cap \mathbf{I}_j\right)} bTd_{iji'j'n}$$

$$\forall s \in (\mathbf{S}^{FIS} \cap \mathbf{S}^{B}), j', i' \in \left(\mathbf{I}_{j'} \cap \mathbf{I}_s^C\right), n = 1 \quad \text{(S8.33)}$$

$$\sum_{i \in \mathbf{I}_j} \sum_{i' \in \mathbf{I}_{j'}} bTd_{iji'j'n} \le \min\left[B_j^{\max}, B_{j'}^{\max}\right] \cdot zD_{jj'n} \qquad \forall j, j' \in \mathbf{CJ}_j^F, j \ne j', n > 1 \qquad \text{(S8.34)}$$

$$\sum_{i \in \mathbf{I}_j} \sum_{i' \in \mathbf{I}_{j'}} bTd_{iji'j'n} \le \min\left[B_j^{\max}, B_{j'}^{\max}\right] \cdot zD_{jj'n}$$

$$\forall j, j' \in \mathbf{CJ}_j^F, j \ne j', n = 1, \sum_{s \in \mathbf{S}^{FIS}} \sum_{i \in \left(\mathbf{I}_j \cap \mathbf{I}_s^P\right)} \sum_{i' \in \left(\mathbf{I}_{j'} \cap \mathbf{I}_s^C\right)} bs0_{ij} > 0 \qquad \text{(S8.35)}$$

$$T_{sjn} \ge T_{j'(n-1)}^{\mathrm{f}} - M \cdot \left[1 - \sum_{i' \in \left(\mathbf{I}_{j'} \cap \mathbf{I}_s^C\right)} \sum_{n \le n' \le n+\Delta n} w_{i'j'nn'}\right]$$

$$\forall s \in \mathbf{S}^{FIS}, j \ne j', j \in \mathbf{J}_s^P, j' \in \mathbf{J}_s^C, 1 < n < N \quad \text{(S8.36)}$$

Allowing processing units to store materials

$$bs_{ijn} \le B_{ij}^{\max} \cdot ys_{ijn} \qquad \forall j, i \in \left(\mathbf{I}_j \cap \mathbf{I}^P\right), n \qquad \text{(S8.37)}$$

Allocation constraints

$$\sum_{i \in \left(\mathbf{I}_j \cap \mathbf{I}^P\right)} ys_{ijn} \le 1 - \sum_{i \in \mathbf{I}_j} \sum_{n-\Delta n \le n' \le n} \sum_{n \le n'' \le n'+\Delta n} w_{ijn'n''} \qquad \forall j, n \qquad \text{(S8.38)}$$

Tightening constraints for minimization of makespan

$$\sum_{i \in \mathbf{I}_j} \sum_{n} \sum_{n \le n' \le n+\Delta n} \left(\alpha_{ij} \cdot w_{ijnn'} + \beta_{ij} \cdot b_{ijnn'}\right) \le MS \qquad \forall j \qquad \text{(S8.39)}$$

Bounds and fixing

$$T_{jn}^{\mathrm{s}} \le H, T_{jn}^{\mathrm{f}} \le H \qquad \forall j, n \qquad \text{(S8.40,41)}$$

$$ST_{sn}^{M2} \le ST_s^{max} \qquad \forall s \in \mathbf{S}^{FIS}, n \in N \qquad \text{(S8.42)}$$

$$bs_{ijn} \leq bs0_{ij} \qquad \forall j, i \in (\mathbf{I}_j \cap \mathbf{I}^P), n = 1 \qquad \text{(S8.43)}$$

$$ys_{ijn} = 0, bs_{ijn} = 0 \qquad \forall i \notin \mathbf{I}^P, j, n \qquad \text{(S8.44,45)}$$

$$w_{ijnn'} = 0, b_{ijnn'} = 0 \qquad \forall i, j, n, n' < n \qquad \text{(S8.46)}$$

$$w_{ijnn'} = 0, b_{ijnn'} = 0 \qquad \forall j, i \notin \mathbf{I}_j, n, n' \qquad \text{(S8.47)}$$

$$bTd_{iji'j'n} = bTi_{iji'j'n} = zI_{jj'n} = zD_{jj'n} = ys_{ijn} = bs_{ijn} = 0$$

$$\forall i, i', j, j', n = 1, bs0_{ij} = 0 \qquad \text{(S8.48)}$$

$$b_{ijnn'}, bs_{ijn}, bTi_{iji'j'n}, bTd_{iji'j'n}, MS, ST_{sn}^{M2}, T_{sjn}, T_{jn}^{\mathrm{s}}, T_{jn}^{\mathrm{f}} \geq 0 \qquad \text{(S8.49)}$$

$$w_{ijnn'}, ys_{ijn}, zD_{jj'n}, zI_{jj'n} \in \{0,1\} \qquad \text{(S8.50)}$$

The mathematical model **M3** consists of constraints (S8.1), (S8.4) - (S8.38) and (S8.40)-(S8.50) for maximization of profit, and (S8.2) - (S8.50) for minimization of makespan.

S9. Extended Model M1 (EM1)

In this section, the model **M1** is extended to handle multipurpose batch plants with complicated features, including various fraction of production for tasks, zero wait and no intermediate storage policies.

Objective functions

Maximization of profit

$$z = \sum_{s \in \mathbf{S}^P} \left[p_s \cdot \sum_j \left(\sum_{i \in (\mathbf{I}_j \cap \mathbf{I}_s^P \setminus \mathbf{I}^V)} \sum_n \sum_{n \le n' \le n + \Delta n} (\rho_{si} \cdot b_{ijnn'}) + \sum_{i \in (\mathbf{I}_j \cap \mathbf{I}_s^P \cap \mathbf{I}^V)} \sum_n \sum_{n \le n' \le n + \Delta n} bv_{sijnn'} \right) \right] \quad \text{(S9.1)}$$

Minimization of makespan

$$T_{jn}^{\mathrm{f}} \le MS \qquad \forall j, n = N \quad \text{(S9.2)}$$

$$ST0_s + \sum_j \left(\sum_{i \in (\mathbf{I}_j \cap \mathbf{I}_s^P \setminus \mathbf{I}^V)} \sum_n \sum_{n \le n' \le n + \Delta n} (\rho_{si} \cdot b_{ijnn'}) + \sum_{i \in (\mathbf{I}_j \cap \mathbf{I}_s^P \cap \mathbf{I}^V)} \sum_n \sum_{n \le n' \le n + \Delta n} bv_{sijnn'} \right) \ge D_s \qquad \forall s \in \mathbf{S}^P \quad \text{(S9.3)}$$

Tightening constraints

$$\sum_{i \in (\mathbf{I}_j \cap \mathbf{I}_s^P)} \left(\sum_{n - \Delta n \le n' \le n} w_{ijn'n} + ys_{ijn} \right) \ge \sum_{i' \in (\mathbf{I}_{j'} \cap \mathbf{I}_s^C)} \sum_{n+1 \le n' \le n+1+\Delta n} w_{i'j'(n+1)n'} + zI_{jj'(n+1)} - 1$$
$$\forall s \in (\mathbf{S}^{FIS} \cup \mathbf{S}^{UIS}), j \in \mathbf{J}_s^P, j' \in \mathbf{CJ0}_j, j \ne j', j' \in \mathbf{J}_s^C, n < N \quad \text{(S9.4)}$$

$$\sum_{i' \in (\mathbf{I}_{j'} \cap \mathbf{I}_s^C)} \sum_{n+1 \le n' \le n+1+\Delta n} w_{i'j'(n+1)n'} \ge \sum_{i \in (\mathbf{I}_j \cap \mathbf{I}_s^P)} \left[\sum_{n - \Delta n \le n' \le n} w_{ijn'n} + ys_{ijn} \right] + zI_{jj'(n+1)} - 1$$
$$\forall s \in (\mathbf{S}^{FIS} \cup \mathbf{S}^{UIS}), j \in \mathbf{J}_s^P, j' \in \mathbf{CJ0}_j, j \ne j', j' \in \mathbf{J}_s^C, n < N \quad \text{(S9.5)}$$

$$\sum_{i \in (\mathbf{I}_j \cap \mathbf{I}_s^P)} \left(\sum_{n - \Delta n \le n' \le n} w_{ijn'n} + ys_{ijn} \right) \ge \sum_{i' \in (\mathbf{I}_{j'} \cap \mathbf{I}_s^C)} \sum_{n+1 \le n' \le n+1+\Delta n} w_{i'j'(n+1)n'} + zD_{jj'(n+1)} - 1$$
$$\forall s \in (\mathbf{S}^{ZW} \cup \mathbf{S}^{NIS}), j \in \mathbf{J}_s^P, j' \in \mathbf{CJ0}_j, j \ne j', j' \in \mathbf{J}_s^C, n < N \quad \text{(S9.6)}$$

$$\sum_{i' \in (\mathbf{I}_{j'} \cap \mathbf{I}_s^C)} \sum_{n+1 \le n' \le n+1+\Delta n} w_{i'j'(n+1)n'} \ge \sum_{i \in (\mathbf{I}_j \cap \mathbf{I}_s^P)} \left[\sum_{n - \Delta n \le n' \le n} w_{ijn'n} + ys_{ijn} \right] + zD_{jj'(n+1)} - 1$$
$$\forall s \in (\mathbf{S}^{ZW} \cup \mathbf{S}^{NIS}), j \in \mathbf{J}_s^P, j' \in \mathbf{CJ0}_j, j \ne j', j' \in \mathbf{J}_s^C, n < N \quad \text{(S9.7)}$$

$$zI_{jj'n} \geq zD_{jj'n} + \sum_{i\in(\mathbf{I}_j\cap\mathbf{I}^{PF})} \sum_{n-1-\Delta n\leq n'\leq n-1} \left(w_{ijn'(n-1)} + ys_{ij(n-1)}\right) - 1$$

$$\forall j,j' \in \mathbf{CJ}_j^F, j \neq j', n > 1 \quad \text{(S9.8)}$$

$$2\cdot zI_{jj'n} \leq \sum_{i\in\mathbf{I}_j} \sum_{n-1-\Delta n\leq n'\leq n-1} w_{ijn'(n-1)} + \sum_{i\in(\mathbf{I}_j\cap\mathbf{I}^P)} ys_{ij(n-1)} + \sum_{i'\in\mathbf{I}_{j'}} \sum_{n\leq n'\leq n+\Delta n} w_{i'j'nn'}$$

$$\forall j,j' \in (\mathbf{CJ}_j^F \cup \mathbf{CJ}_j^U), j \neq j', \sum_{s\in(\mathbf{S}^R\cup\mathbf{S}^P),s:(j,j'\in\mathbf{J}_s)} 1 = 0, n > 1 \quad \text{(S9.9)}$$

$$2\cdot zD_{jj'n} \leq \sum_{i\in\mathbf{I}_j} \sum_{n-1-\Delta n\leq n'\leq n-1} w_{ijn'(n-1)} + \sum_{i\in(\mathbf{I}_j\cap\mathbf{I}^P)} ys_{ij(n-1)} + \sum_{i'\in\mathbf{I}_{j'}} \sum_{n\leq n'\leq n+\Delta n} w_{i'j'nn'}$$

$$\forall j,j' \in (\mathbf{CJ}_j^{NIS} \cup \mathbf{CJ}_j^{ZW}), j \neq j', \sum_{s\in(\mathbf{S}^R\cup\mathbf{S}^P),s:(j,j'\in\mathbf{J}_s)} 1 = 0, n > 1 \quad \text{(S9.10)}$$

$$\sum_{j'\in\mathbf{J}_s^C,j'\neq j} zD_{jj'n} + ys_{ijn} + \sum_{i'\in\left(\mathbf{I}_j\cap\mathbf{I}_s^C\right)} \sum_{n\leq n'\leq n+\Delta n} w_{i'jnn'} \geq \sum_{n-1-\Delta n\leq n'\leq n-1} w_{ijn'(n-1)} + ys_{ij(n-1)}$$

$$\forall s \in (\mathbf{S}^{ZW} \cup \mathbf{S}^{NIS}), j, i \in \left(\mathbf{I}_j \cap \mathbf{I}_s^P\right), n > 1 \quad \text{(S9.11)}$$

$$\sum_{j\in\mathbf{J}_s^P,j\neq j'} zD_{jj'n} + \sum_{i\in\left(\mathbf{I}_{j'}\cap\mathbf{I}_s^P\right)} \left(\sum_{n-1-\Delta n\leq n'\leq n-1} w_{ij'n'(n-1)} + ys_{ij'(n-1)}\right)$$

$$\geq \sum_{n\leq n'\leq n+\Delta n} w_{i'j'nn'} \quad \forall s \in (\mathbf{S}^{ZW} \cup \mathbf{S}^{NIS}), j', i' \in \left(\mathbf{I}_{j'} \cap \mathbf{I}_s^C\right), n > 1 \quad \text{(S9.12)}$$

Capacity constraints

$$B_{ij}^{\min}\cdot w_{ijnn'} \leq b_{ijnn'} \leq B_{ij}^{\max}\cdot w_{ijnn'} \qquad \forall j, i \in \mathbf{I}_j, n \leq n' \leq n + \Delta n \qquad \text{(S9.13)}$$

Variable fraction for production

$$b_{ijnn'}\cdot \rho_{si}^{min} \leq bv_{sijnn'} \leq b_{ijnn'}\cdot \rho_{si}^{max}$$

$$\forall s, j, i \in \left(\mathbf{I}^V \cap \mathbf{I}_j \cap \mathbf{I}_s^P\right), n, n \leq n' \leq n + \Delta n \quad \text{(S9.14)}$$

$$\sum_{s:i\in\mathbf{I}_s^P} bv_{sijnn'} = b_{ijnn'} \qquad \forall j, i \in \left(\mathbf{I}^V \cap \mathbf{I}_j\right), n, n \leq n' \leq n + \Delta n \qquad \text{(S9.15)}$$

$$bs_{ijn}\cdot \rho_{si}^{min} \leq bsv_{sijn} \leq bs_{ijn}\cdot \rho_{si}^{max} \qquad \forall s, j, i \in \left(\mathbf{I}^V \cap \mathbf{I}_j \cap \mathbf{I}^P \cap \mathbf{I}_s^P\right), n > 1 \qquad \text{(S9.16)}$$

$$bs_{ijn}\cdot \rho_{si}^{min} \leq bsv_{sijn} \leq bs_{ijn}\cdot \rho_{si}^{max}$$

$$\forall s, j, i \in \left(\mathbf{I}^V \cap \mathbf{I}_j \cap \mathbf{I}^P \cap \mathbf{I}_s^P\right), n = 1, bs0_{ij} > 0 \quad \text{(S9.17)}$$

$$\sum_{s\in\mathbf{S}_i^P} bsv_{sijn} = bs_{ijn} \qquad \forall j, i \in \left(\mathbf{I}^V \cap \mathbf{I}_j \cap \mathbf{I}^P\right), n > 1 \qquad \text{(S9.18)}$$

$$\sum_{s\in\mathbf{S}_i^P} bsv_{sijn} = bs_{ijn} \qquad \forall j, i \in \left(\mathbf{I}^V \cap \mathbf{I}_j \cap \mathbf{I}^P\right), n = 1, bs0_{ij} > 0 \tag{S9.19}$$

Material balance constraints

$$\begin{aligned} ST_{sn}^{M1} = ST_{s(n-1)}^{M1} &+ \sum_j \sum_{i\in\left(\mathbf{I}_j\cap\mathbf{I}_s^P\setminus\mathbf{I}^V\right)} \sum_{n-1-\Delta n\le n'\le n-1} \rho_{si}\cdot b_{ijn'(n-1)} \\ &+ \sum_j \sum_{i\in\left(\mathbf{I}_j\cap\mathbf{I}_s^P\cap\mathbf{I}^V\right)} \sum_{n-1-\Delta n\le n'\le n-1} bv_{sijn'(n-1)} \\ &+ \sum_j \sum_{i\in\left(\mathbf{I}_j\cap\mathbf{I}_s^C\right)} \sum_{n\le n'\le n+\Delta n} \rho_{si}\cdot b_{ijnn'} \qquad \forall s, n > 1 \end{aligned} \tag{S9.20}$$

$$\begin{aligned} ST_{sn}^{M1} = ST0_s &+ \sum_j \sum_{i\in\left(\mathbf{I}_j\cap\mathbf{I}_s^P\setminus\mathbf{I}^V\right)} \rho_{si}\cdot bs0_{ij} + \sum_j \sum_{i\in\left(\mathbf{I}_j\cap\mathbf{I}_s^P\cap\mathbf{I}^V\right)} bsv0_{sij} \\ &+ \sum_j \sum_{i\in\left(\mathbf{I}_j\cap\mathbf{I}_s^C\right)} \sum_{n\le n'\le n+\Delta n} \rho_{si}\cdot b_{ijnn'} \qquad \forall s, n = 1 \end{aligned} \tag{S9.21}$$

$$\sum_j \sum_{i\in\left(\mathbf{I}_j\cap\mathbf{I}_s^P\cap\mathbf{I}^P\setminus\mathbf{I}^V\right)} \rho_{si}\cdot bs_{ijn} + \sum_j \sum_{i\in\left(\mathbf{I}_j\cap\mathbf{I}_s^P\cap\mathbf{I}^P\cap\mathbf{I}^V\right)} bsv_{sijn} \le ST_{sn}^{M1} \qquad \forall s \in \left(\mathbf{S}^{FIS}\cup\mathbf{S}^{NIS}\right), n \tag{S9.22}$$

Duration constraints

$$T_{jn}^{\mathrm{f}} \ge T_{jn}^{\mathrm{s}} + \sum_{i\in\mathbf{I}_j} \sum_{n\le n'\le n+\Delta n} \left(\alpha_{ij}\cdot w_{ijnn'} + \beta_{ij}\cdot b_{ijnn'}\right) \qquad \forall j, n \tag{S9.23}$$

$$\begin{aligned} T_{jn}^{f} \le T_{jn}^{s} &+ \sum_{i\in\mathbf{I}_j} \sum_{n\le n'\le n+\Delta n} \left(a_{ij}\cdot w_{ijnn'} + \beta_{ij}\cdot b_{ijnn'}\right) + M \\ &\cdot \left[1 - \sum_{i\in\left(\mathbf{I}_j\cap\mathbf{I}^{Pzw}\right)} \sum_{n\le n'\le n+\Delta n} w_{ijnn'}\right] \qquad \forall j \in \mathbf{J}^{Pzw}, n \end{aligned} \tag{S9.24}$$

Sequence constraints for different tasks in the same unit

$$T_{j(n+1)}^{\mathrm{s}} \ge T_{jn}^{\mathrm{f}} \qquad \forall j, n < N \tag{S9.25}$$

$$T_{sjn} \le T_{sj(n+1)} \qquad \forall s \in \left(\mathbf{S}^{FIS}\cup\mathbf{S}^{UIS}\right), j \in \mathbf{J}_s^P, n < N-1 \tag{S9.26}$$

$$T_{sjn} \ge T_{jn}^{\mathrm{f}} - M\cdot\left[1 - \sum_{i\in\left(\mathbf{I}_j\cap\mathbf{I}_s^P\right)} \sum_{n-\Delta n\le n'\le n} w_{ijn'n}\right] \forall s \in \left(\mathbf{S}^{FIS}\cup\mathbf{S}^{UIS}\right), j \in \mathbf{J}_s^P, n < N \tag{S9.27}$$

$$T_{sjn} \le T_{j(n+1)}^{\mathrm{s}} + M\cdot\left[1 - \sum_{i\in\left(\mathbf{I}_j\cap\mathbf{I}_s^P\right)} \left(\sum_{n-\Delta n\le n'\le n} w_{ijn'n} + ys_{ijn}\right)\right] \qquad \forall s \in \left(\mathbf{S}^{FIS}\cup\mathbf{S}^{UIS}\right), j \in \mathbf{J}_s^P, n < N \tag{S9.28}$$

Material transfer

Indirect material transfer for NIS and UIS

$$T_{jn}^{\mathrm{f}} \le T_{j'(n+1)}^{\mathrm{s}} + M \cdot \left[1 - zI_{jj'(n+1)}\right] \qquad \forall j,j' \in (\mathbf{CJ}_j^F \cup \mathbf{CJ}_j^U), j \ne j', n < N \qquad \text{(S9.29)}$$

$$\sum_{j'} \sum_{i' \in \left(\mathbf{I}_s^C \cap \mathbf{I}_{j'}\right)} \left(-\rho_{si'} \cdot \sum_{n \le n' \le n+\Delta n} b_{i'j'nn'}\right) \le ST_{s(n-1)}^{M1} + \sum_j \sum_{j'} \sum_{i \in \left(\mathbf{I}_s^P \cap \mathbf{I}_j\right)} \sum_{i' \in \left(\mathbf{I}_s^C \cap \mathbf{I}_{j'}\right)} bTi_{iji'j'n}$$

$$\forall s \in (\mathbf{S}^{FIS} \cup \mathbf{S}^{UIS}), n > 1 \qquad \text{(S9.30)}$$

$$\sum_{j'} \sum_{i' \in \left(\mathbf{I}_s^C \cap \mathbf{I}_{j'}\right)} \left(-\rho_{si'} \cdot \sum_{n \le n' \le n+\Delta n} b_{i'j'nn'}\right) \le ST0_s + \sum_j \sum_{j'} \sum_{i \in \left(\mathbf{I}_s^P \cap \mathbf{I}_j\right)} \sum_{i' \in \left(\mathbf{I}_s^C \cap \mathbf{I}_{j'}\right)} bTi_{iji'j'n}$$

$$\forall s \in (\mathbf{S}^{FIS} \cup \mathbf{S}^{UIS} \cup \mathbf{S}^B), n = 1 \qquad \text{(S9.31)}$$

$$\sum_{i' \notin \mathbf{I}^V : i' = i} \sum_{n-1-\Delta n \le n' \le n-1} \rho_{si'} \cdot b_{i'jn'(n-1)} + \sum_{i' \in \mathbf{I}^V : i' = i} \sum_{n-1-\Delta n \le n' \le n-1} bv_{si'jn'(n-1)}$$

$$\ge \sum_{j'} \sum_{i' \in \left(\mathbf{I}_{j'} \cap \mathbf{I}_s^C\right)} bTi_{iji'j'n} \quad \forall s \in (\mathbf{S}^{FIS} \cup \mathbf{S}^{UIS}), j, i \in \left(\mathbf{I}_j \cap \mathbf{I}_s^P\right), n > 1 \qquad \text{(S9.32)}$$

$$-\rho_{si'} \cdot \sum_{n \le n' \le n+\Delta n} b_{i'j'nn'} \ge \sum_j \sum_{i \in \left(\mathbf{I}_j \cap \mathbf{I}_s^P\right)} bTi_{iji'j'n}$$

$$\forall s \in (\mathbf{S}^{FIS} \cup \mathbf{S}^{UIS}), j', i' \in \left(\mathbf{I}_{j'} \cap \mathbf{I}_s^C\right), n > 1 \qquad \text{(S9.33)}$$

$$-\rho_{si'} \cdot \sum_{n \le n' \le n+\Delta n} b_{i'j'nn'} \ge \sum_j \sum_{i \in \left(\mathbf{I}_j \cap \mathbf{I}_s^P\right)} bTi_{iji'j'n}$$

$$\forall s \in ((\mathbf{S}^{FIS} \cup \mathbf{S}^{UIS}) \cap \mathbf{S}^B), j', i' \in \left(\mathbf{I}_{j'} \cap \mathbf{I}_s^C\right), n = 1 \qquad \text{(S9.34)}$$

$$\sum_{i \in \mathbf{I}_j} \sum_{i' \in \mathbf{I}_{j'}} bTi_{iji'j'n} \le \min\left[B_j^{\max}, B_{j'}^{\max}\right] \cdot zI_{jj'n} \quad \forall j,j' \in \left(\mathbf{CJ}_j^F \cup \mathbf{CJ}_j^U\right), j \ne j', n > 1 \qquad \text{(S9.35)}$$

$$\sum_{i \in \mathbf{I}_j} \sum_{i' \in \mathbf{I}_{j'}} bTi_{iji'j'n} \le \min\left[B_j^{\max}, B_{j'}^{\max}\right] \cdot zI_{jj'n}$$

$$\forall j,j' \in (\mathbf{CJ}_j^F \cup \mathbf{CJ}_j^U), j \ne j', n = 1, \sum\nolimits_{s \in \mathbf{S}^{FIS}} \sum\nolimits_{i \in \left(\mathbf{I}_j \cap \mathbf{I}_s^P\right)} \sum\nolimits_{i' \in \left(\mathbf{I}_{j'} \cap \mathbf{I}_s^C\right)} bs0_{ij} > 0 \qquad \text{(S9.36)}$$

$$T_{sjn} \le T_{j'(n+2)}^{\mathrm{s}} + M \cdot \left[1 - \sum_{i' \in \left(\mathbf{I}_{j'} \cap \mathbf{I}_s^C\right)} \sum_{n+2 \le n' \le n+2+\Delta n} w_{i'j'(n+2)n'}\right]$$

$$\forall s \in (\mathbf{S}^{FIS} \cup \mathbf{S}^{UIS}), j \ne j', j \in \mathbf{J}_s^P, j' \in \mathbf{J}_s^C, n < N - 1 \qquad \text{(S9.37)}$$

Direct material transfer for FIS, NIS and ZW

$$T_{j'n}^{\mathrm{f}} \le T_{jn}^{\mathrm{f}} + M \cdot \left[1 - zD_{jj'(n+1)}\right] \qquad \forall j,j' \in (\mathbf{CJ}_j^F \cup \mathbf{CJ}_j^{NIS}), j \ne j', n < N \qquad \text{(S9.38)}$$

$$T^{\mathrm{s}}_{j'(n+1)} \leq T^{\mathrm{f}}_{jn} + M \cdot \left[2 - zD_{jj'(n+1)} - \sum_{i \in (\mathbf{I}_j \cap \mathbf{I}^{Pzw})} \sum_{n-\Delta n \leq n' \leq n} w_{ijn'n}\right]$$

$$\forall j, j' \in \mathbf{CJ}^{ZW}_j, j \neq j', n < N \quad \text{(S9.39)}$$

$$T^{\mathrm{s}}_{j'(n+1)} \leq T^{\mathrm{f}}_{jn} + M \cdot \left[2 - \sum_{i' \in (\mathbf{I}_{j'} \cap \mathbf{I}^C_s)} \sum_{n+1 \leq n' \leq n+1+\Delta n} w_{i'j'(n+1)n'} - \sum_{i \in (\mathbf{I}_j \cap \mathbf{I}^P_s)} \sum_{n-\Delta n \leq n' \leq n} w_{ijn'n}\right]$$

$$\forall s \in \mathbf{S}^{ZW}, j \in \mathbf{J}^C_s, j' \in \mathbf{J}^P_s, j = j', n < N \quad \text{(S9.40)}$$

$$T^{\mathrm{f}}_{jn} \leq T^{s}_{j'(n+1)} + M \cdot \left[1 - zD_{jj'(n+1)}\right] \qquad \forall j, j' \in (\mathbf{CJ}^{NIS}_j \cup \mathbf{CJ}^{ZW}_j), j \neq j', n < N \quad \text{(S9.41)}$$

$$\sum_j \sum_{i \in (\mathbf{I}_j \cap \mathbf{I}^P_s \setminus \mathbf{I}^V)} \left[\rho_{si} \cdot \sum_{n-1-\Delta n \leq n' \leq n-1} b_{ijn'(n-1)}\right] + \sum_j \sum_{i \in (\mathbf{I}_j \cap \mathbf{I}^P_s \cap \mathbf{I}^V)} \sum_{n-1-\Delta n \leq n' \leq n-1} bv_{sijn'(n-1)}$$
$$+ ST^{M1}_{s(n-1)}$$
$$\leq ST^{\max}_s + \sum_j \sum_{j'} \sum_{i \in (\mathbf{I}_j \cap \mathbf{I}^P_s)} \sum_{i' \in (\mathbf{I}_{j'} \cap \mathbf{I}^C_s)} bTd_{iji'j'n} + \sum_j \sum_{i \in (\mathbf{I}_j \cap \mathbf{I}^P_s \setminus \mathbf{I}^V)} \rho_{si} \cdot bs_{ijn}$$
$$+ \sum_j \sum_{i \in (\mathbf{I}_j \cap \mathbf{I}^P_s \cap \mathbf{I}^V)} bsv_{sijn} \qquad \forall s \in \mathbf{S}^{FIS}, n > 1 \quad \text{(S9.42)}$$

$$\sum_j \sum_{i \in (\mathbf{I}_j \cap \mathbf{I}^P_s \setminus \mathbf{I}^V)} \rho_{sij} \cdot bs0_{ij} + \sum_j \sum_{i \in (\mathbf{I}_j \cap \mathbf{I}^P_s \cap \mathbf{I}^V)} bsv0_{sij} + ST0_s$$
$$\leq ST^{\max}_s + \sum_j \sum_{j'} \sum_{i \in (\mathbf{I}_j \cap \mathbf{I}^P_s)} \sum_{i' \in (\mathbf{I}_{j'} \cap \mathbf{I}^C_s)} bTd_{iji'j'n} \sum_j \sum_{i \in (\mathbf{I}_j \cap \mathbf{I}^P_s \setminus \mathbf{I}^V)} \rho_{si} \cdot bs_{ijn}$$
$$+ \sum_j \sum_{i \in (\mathbf{I}_j \cap \mathbf{I}^P_s \cap \mathbf{I}^V)} bsv_{sijn} \qquad \forall s \in (\mathbf{S}^{FIS} \cap \mathbf{S}^B), n = 1 \quad \text{(S9.43)}$$

$$\sum_{i' \notin \mathbf{I}^V : i' = i} \left[\rho_{si'} \cdot \left(\sum_{n-1-\Delta n \leq n' \leq n-1} b_{i'jn'(n-1)} + bs_{i'j(n-1)}\right)\right]$$
$$+ \sum_{i' \in \mathbf{I}^V : i' = i} \left[\sum_{n-1-\Delta n \leq n' \leq n-1} bv_{si'jn'(n-1)} + bsv_{si'j(n-1)}\right]$$
$$\geq \sum_{j'} \sum_{i' \in (\mathbf{I}^C_s \cap \mathbf{I}_{j'})} bTd_{iji'j'n} + \sum_{i' \notin \mathbf{I}^V : i' = i} \left(\rho_{si'} \cdot bs_{i'jn}\right) + \sum_{i' \in \mathbf{I}^V : i' = i} bsv_{si'jn}$$

$$\forall s \in \mathbf{S}^{FIS}, j, i \in \left(\mathbf{I}_j \cap \mathbf{I}^P_s\right), n > 1 \quad \text{(S9.44)}$$

$$\sum_{i'\notin \mathbf{I}^V:i'=i}(\rho_{si'}\cdot bs0_{i'j})+\sum_{i'\in \mathbf{I}^V:i'=i}bsv0_{si'j}$$

$$\geq\sum_{j'}\sum_{i'\in\left(\mathbf{I}_s^C\cap\mathbf{I}_{j'}\right)}bTd_{iji'j'n}+\sum_{i'\notin \mathbf{I}^V:i'=i}\left(\rho_{si'}\cdot bs_{i'jn}\right)+\sum_{i'\in \mathbf{I}^V:i'=i}bsv_{si'jn}$$

$$\forall s\in \mathbf{S}^{FIS},j,i\in\left(\mathbf{I}_j\cap\mathbf{I}_s^P\right),n=1,bs0_{ij}>0 \quad \text{(S9.45)}$$

$$-\rho_{si'}\cdot\sum_{n\leq n'\leq n+\Delta n}b_{i'j'nn'}\geq\sum_{j}\sum_{i\in\left(\mathbf{I}_s^P\cap\mathbf{I}_j\right)}bTd_{iji'j'n}$$

$$\forall s\in \mathbf{S}^{FIS},j',i'\in\left(\mathbf{I}_{j'}\cap\mathbf{I}_s^C\right),n>1 \quad \text{(S9.46)}$$

$$-\rho_{si'}\cdot\sum_{n\leq n'\leq n+\Delta n}b_{i'j'nn'}\geq\sum_{j}\sum_{i\in\left(\mathbf{I}_s^P\cap\mathbf{I}_j\right)}bTd_{iji'j'n}$$

$$\forall s\in (\mathbf{S}^{FIS}\cap\mathbf{S}^B),j',i'\in\left(\mathbf{I}_{j'}\cap\mathbf{I}_s^C\right),n=1 \quad \text{(S9.47)}$$

$$\sum_{i'\notin \mathbf{I}^V:i'=i}\left[\rho_{si'}\cdot\left(\sum_{n-1-\Delta n\leq n'\leq n-1}b_{i'jn'(n-1)}+bs_{i'j(n-1)}\right)\right]$$

$$+\sum_{i'\in \mathbf{I}^V:i'=i}\left[\sum_{n-1-\Delta n\leq n'\leq n-1}bv_{si'jn'(n-1)}+bsv_{si'j(n-1)}\right]$$

$$=\sum_{j'}\sum_{i'\in\left(\mathbf{I}_s^C\cap\mathbf{I}_{j'}\right)}bTd_{iji'j'n}+\sum_{i'\notin \mathbf{I}^V:i'=i}\left(\rho_{si'}\cdot bs_{i'jn}\right)+\sum_{i'\in \mathbf{I}^V:i'=i}bsv_{si'jn}$$

$$\forall s\in (\mathbf{S}^{ZW}\cup\mathbf{S}^{NIS}),j,i\in\left(\mathbf{I}_j\cap\mathbf{I}_s^P\right),n>1 \quad \text{(S9.48)}$$

$$\sum_{i'\notin \mathbf{I}^V:i'=i}\left[\rho_{si'}\cdot bs0_{i'j}\right]+\sum_{i'\in \mathbf{I}^V:i'=i}bsv0_{si'j}$$

$$=\sum_{j'}\sum_{i'\in\left(\mathbf{I}_s^C\cap\mathbf{I}_{j'}\right)}bTd_{iji'j'n}+\sum_{i'\notin \mathbf{I}^V:i'=i}\left(\rho_{si'}\cdot bs_{i'jn}\right)+\sum_{i'\in \mathbf{I}^V:i'=i}bsv_{si'jn}$$

$$\forall s\in \mathbf{S}^{NIS},j,i\in\left(\mathbf{I}_j\cap\mathbf{I}_s^P\right),n=1,bs0_{ij}\geq 0 \quad \text{(S9.49)}$$

$$-\rho_{si'}\cdot\sum_{n\leq n'\leq n+\Delta n}b_{i'j'nn'}=\sum_{j}\sum_{i\in\left(\mathbf{I}_s^P\cap\mathbf{I}_j\right)}bTd_{iji'j'n}$$

$$\forall s\in (\mathbf{S}^{ZW}\cup\mathbf{S}^{NIS}),j',i'\in\left(\mathbf{I}_{j'}\cap\mathbf{I}_s^C\right),n>1 \quad \text{(S9.50)}$$

$$-\rho_{si'}\cdot\sum_{n\leq n'\leq n+\Delta n}b_{i'j'nn'}=\sum_{j}\sum_{i\in\left(\mathbf{I}_s^P\cap\mathbf{I}_j\right)}bTd_{iji'j'n}$$

$$\forall s\in (\mathbf{S}^{NIS}\cap\mathbf{S}^B),j',i'\in\left(\mathbf{I}_{j'}\cap\mathbf{I}_s^C\right),n=1 \quad \text{(S9.51)}$$

$$\sum_{i\in\mathbf{I}_j}\sum_{i'\in\mathbf{I}_{j'}}bTd_{iji'j'n}\leq\min\left[B_j^{\max},B_{j'}^{\max}\right]\cdot zD_{jj'n}$$

$$\forall j, j' \in \left(\mathbf{CJ}_j^F \cup \mathbf{CJ}_j^{ZW} \cup \mathbf{CJ}_j^{NIS}\right), j \neq j', n > 1 \quad \text{(S9.52)}$$

$$\sum_{i \in \mathbf{I}_j} \sum_{i' \in \mathbf{I}_{j'}} bTd_{iji'j'n} \leq \min\left[B_j^{\max}, B_{j'}^{\max}\right] \cdot zD_{jj'n}$$

$$\forall j, j' \in (\mathbf{CJ}_j^F \cup \mathbf{CJ}_j^{NIS}), j \neq j', n = 1, \sum_{s \in \mathbf{S}^{FIS}} \sum_{i \in (\mathbf{I}_j \cap \mathbf{I}_s^P)} \sum_{i' \in \left(\mathbf{I}_{j'} \cap \mathbf{I}_s^C\right)} bs0_{ij} > 0 \quad \text{(S9.53)}$$

Sequence constraints for FIS storage policy

$$T_{sjn} \geq T_{j'(n-1)}^{\mathrm{f}} - M \cdot \left[1 - \sum_{i' \in \left(\mathbf{I}_{j'} \cap \mathbf{I}_s^C\right)} \sum_{n \leq n' \leq n + \Delta n} w_{i'j'nn'}\right]$$

$$\forall s \in \mathbf{S}^{FIS}, j \neq j', j \in \mathbf{J}_s^P, j' \in \mathbf{J}_s^C, 1 < n < N \quad \text{(S9.54)}$$

Allowing processing units to store materials

$$bs_{ijn} \leq B_{ij}^{\max} \cdot ys_{ijn} \qquad \forall j, i \in \left(\mathbf{I}_j \cap \mathbf{I}^P\right), n \quad \text{(S9.55)}$$

$$bsv_{sijn} + \sum_{n - \Delta n \leq n' \leq n} bv_{sijn'n} \geq bsv_{sij(n+1)} \qquad \forall s, j, i \in \left(\mathbf{I}^V \cap \mathbf{I}_j \cap \mathbf{I}^P \cap \mathbf{I}_s^P\right), n < N \quad \text{(S9.56)}$$

Allocation constraints

$$\sum_{i \in (\mathbf{I}_j \cap \mathbf{I}^P)} ys_{ijn} \leq 1 - \sum_{i \in \mathbf{I}_j} \sum_{n - \Delta n \leq n' \leq n} \sum_{n \leq n'' \leq n' + \Delta n} w_{ijn'n''} \qquad \forall j, n \quad \text{(S9.57)}$$

Tightening constraint for minimization of makespan

$$\sum_{i \in \mathbf{I}_j} \sum_{n} \sum_{n \leq n' \leq n + \Delta n} (\alpha_{ij} \cdot w_{ijnn'} + \beta_{ij} \cdot b_{ijnn'}) \leq MS \qquad \forall\, j \quad \text{(S9.58)}$$

Last event for ZW

$$\sum_{j} \sum_{i \in \left(\mathbf{I}_j \cap \mathbf{I}_s^P\right)} \sum_{n - \Delta n \leq n' \leq n} w_{ijn'n} = 0 \qquad \forall s \in \mathbf{S}^{ZW}, n = N \quad \text{(S9.59)}$$

$$\sum_{j} \sum_{i \in \left(\mathbf{I}_j \cap \mathbf{I}_s^P\right)} \sum_{n - \Delta n \leq n' \leq n} b_{ijn'n} = 0 \qquad \forall s \in \mathbf{S}^{ZW}, n = N \quad \text{(S9.60)}$$

Bounds and fixing

$$T_{jn}^{\mathrm{s}} \leq H, T_{jn}^{\mathrm{f}} \leq H \qquad \forall j, n \quad \text{(S9.61,62)}$$

$$ST_{sn}^{M1} \leq ST_s^{max} + \sum_{j \in \mathbf{J}_s^P} \left[\max_{i \in \left(\mathbf{I}_j \cap \mathbf{I}_s^P\right)} \left(\rho_{si} \cdot B_{ij}^{\max}\right)\right] \qquad \forall s \in \mathbf{S}^{FIS}, n \quad \text{(S9.63)}$$

$$ST_{sn}^{M1} \leq 0 \qquad \forall s \in \mathbf{S}^{ZW}, n \quad \text{(S9.64)}$$

$$ST_{sn}^{M1} \leq \sum_{j \in \mathbf{J}_s^P} \left[\max_{i \in \left(\mathbf{I}_j \cap \mathbf{I}_s^P\right)} \left(\rho_{si} \cdot B_{ij}^{\max}\right)\right] \qquad \forall s \in \mathbf{S}^{NIS}, n \quad \text{(S9.65)}$$

$$bs_{ijn} \leq bs0_{ij} \qquad \forall j, i \in (\mathbf{I}_j \cap \mathbf{I}^P), n = 1 \qquad (S9.66)$$

$$bsv_{sijn} \leq bsv0_{sij} \qquad \forall j, i \in (\mathbf{I}^V \cap \mathbf{I}_j \cap \mathbf{I}^P), n = 1 \qquad (S9.67)$$

$$ys_{ijn} = 0, bs_{ijn} = 0, bsv_{sijn} = 0 \qquad \forall i \notin \mathbf{I}^P, j, s, n \qquad (S9.68)$$

$$w_{ijnn'} = 0, b_{ijnn'} = 0 \qquad \forall i, j, n, n' < n \qquad (S9.69)$$

$$w_{ijnn'} = 0, b_{ijnn'} = 0 \qquad \forall j, i \notin \mathbf{I}_j, n, n' \qquad (S9.70)$$

$$bTd_{iji'j'n} = bTi_{iji'j'n} = zI_{jj'n} = zD_{jj'n} = ys_{ijn} = bs_{ijn} = 0$$

$$\forall i, i', j, j', n = 1, bs0_{ij} = 0 \qquad (S9.71)$$

$$t_{sjn} = 0 \qquad \forall s \in (\mathbf{S}^{zw} \cup \mathbf{S}^{NIS}), j, n \qquad (S9.72)$$

$$zD_{jj'n} = 0 \qquad \forall j, j', \sum_{s \in (\mathbf{S}^{FIS} \cup \mathbf{S}^{NIS} \cup \mathbf{S}^{ZW})} \sum_{i \in (\mathbf{I}_j \cap \mathbf{I}_s^P)} \sum_{i' \in (\mathbf{I}_{j'} \cap \mathbf{I}_s^C)} \rho_{si} = 0, n \qquad (S9.73)$$

$$bTd_{iji'j'n} = 0 \qquad \forall j, i \in \mathbf{I}_j, j', i' \in \mathbf{I}_{j'}, \sum_{s \in ((\mathbf{S}^{FIS} \cup \mathbf{S}^{NIS} \cup \mathbf{S}^{ZW}) \cap \mathbf{S}_i^P \cap \mathbf{S}_{i'}^C)} \rho_{si} = 0, n \qquad (S9.74)$$

$$zI_{jj'n} = 0 \qquad \forall j, j', \sum_{s \in (\mathbf{S}^{FIS} \cup \mathbf{S}^{UIS})} \sum_{i \in (\mathbf{I}_j \cap \mathbf{I}_s^P)} \sum_{i' \in (\mathbf{I}_{j'} \cap \mathbf{I}_s^C)} \rho_{si} = 0, n \qquad (S9.75)$$

$$bTi_{iji'j'n} = 0 \qquad \forall s \in (\mathbf{S}^{NIS} \cup \mathbf{S}^{ZW}), j, j', i \in (\mathbf{I}_j \cap \mathbf{I}_s^P), i' \in (\mathbf{I}_{j'} \cap \mathbf{I}_s^C), n \qquad (S9.76)$$

$$bs_{ijn} = ys_{ijn} = bsv_{sijn} = 0 \qquad \forall s \in \mathbf{S}^{ZW}, j, i \in (\mathbf{I}_j \cap \mathbf{I}_s^P), n \qquad (S9.77)$$

$$b_{ijnn'}, bs_{ijn}, bTi_{iji'j'n}, bTd_{iji'j'n}, MS, ST_{sn}^{M1}, T_{sjn}, T_{jn}^{\mathrm{s}}, T_{jn}^{\mathrm{f}}, bsv_{sijn}, bv_{sijnn'} \geq 0 \qquad (S9.78)$$

$$w_{ijnn'}, ys_{ijn}, zD_{jj'n}, zI_{jj'n} \in \{0,1\} \qquad (S9.79)$$

The mathematical model **EM1** consists of constraints (S9.1), (S9.4) - (S9.57) and (S9.59) - (S9.79) for maximization of profit, and (S9.2) - (S9.79) for minimization of makespan.

S10. Differences between T_{sn}^{M2} and T_{sjn}

The difference between T_{sn}^{M2} in model **M2** and T_{sjn} in model **M3** are illustrated using a solution provided in Figure S16. In the production process, state S1 is produced by task T1 and consumed by task T2, which can be performed on unit J2 and J3 with maximum capacities of 40 and 60 mu, respectively. Another state S2 produced by task T2 can be used by a task T3 processed on unit J4 with maximum unit capacity of 50 mu.

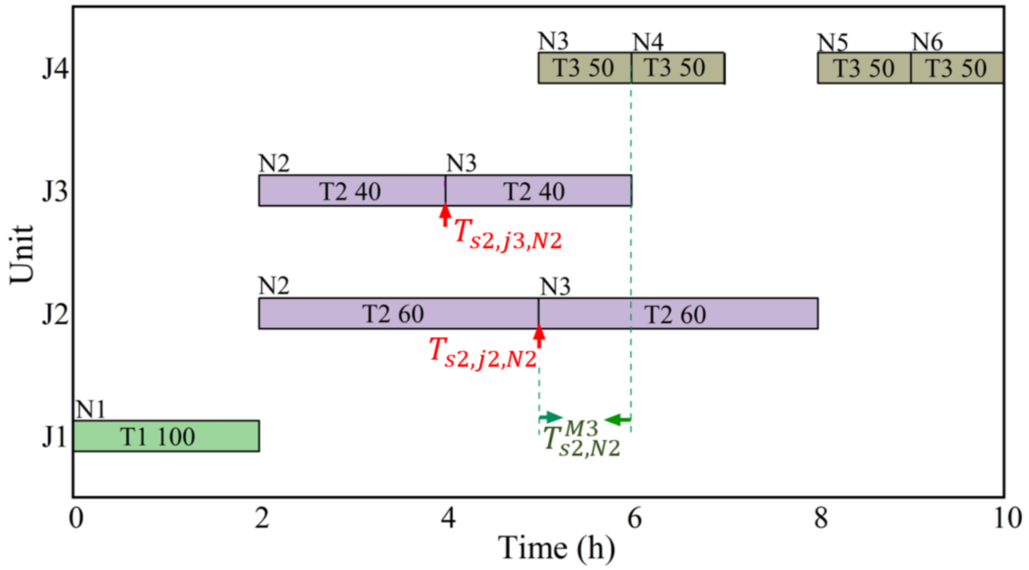

Figure S16. An example solution to illustrate the differences between T_{sn}^{M2} in model **M2** and T_{sjn} in model **M3**

As shown in Figure S16, for state S2 at event point N2, the variable T_{sn}^{M2} in **M2** is not smaller than its related variables T_{sjn} in **M3** on both unit J2 and J3 (i.e. $T_{s2,N2}^{M2} \geq T_{S2,J2,N2}$ and $T_{s2,N2}^{M2} \geq T_{S2,J3,N2}$). This is handled by eq. 60 in **M2** and eq. S8.15 in **M3**.
